# ARR22 overexpression can suppress plant Two-Component Regulatory Systems

**DOI:** 10.1371/journal.pone.0212056

**Published:** 2019-02-11

**Authors:** Niklas Wallmeroth, Daniel Jeschke, Daniel Slane, Janine Nägele, Manikandan Veerabagu, Virtudes Mira-Rodado, Kenneth Wayne Berendzen

**Affiliations:** 1 Department of Plant Physiology at the Center for Plant Molecular Biology (ZMBP), University of Tübingen, Tübingen, Germany; 2 Max-Planck Institute for Developmental Biology, Tübingen, Germany; 3 Department of the Central Facilities at Center for Plant Molecular Biology (ZMBP), University of Tübingen, Tübingen, Germany; Instituto de Biologia Molecular y Celular de Plantas, SPAIN

## Abstract

In plants, several developmental processes are co-coordinated by cytokinins via phosphorylation dependent processes of the Two-Component System (TCS). An outstanding challenge is to track phosphorelay flow from cytokinin perception to its molecular outputs, of which gene activation plays a major role. To address this issue, a kinetic-based reporter system was expounded to track TCS phosphorelay activity *in vivo* that can distinguish between basal and cytokinin dependent effects of overexpressed TCS members. The TCS phosphorelay can be positively activated by cytokinin and inhibited by pharmaceuticals or naturally interfering components. In this case we took advantage of the phosphohistidine-phosphatase Arabidopsis Response Regulator (ARR) 22 and investigated its phosphocompetition with other TCS members in regulating promoters of *ARR5* and *WUS* in *Arabidopsis thaliana* cell culture protoplasts. In congruency with the proposed function of ARR22, overexpression of ARR22 blocked the activation of all B-type ARRs in this study in a TCS dependent manner. Furthermore, this effect could not be mimicked by A-type response regulator overexpression or compensated by AHP overexpression. Compared to other reporter assays, ours mimicked effects previously observed only in transgenic plants for all of the TCS proteins studied, suggesting that it is possible to expose phosphocompetition. Thus, our approach can be used to investigate gene signaling networks involving the TCS by leveraging ARR22 as a TCS inhibitor along with B-type ARR overexpression.

## Introduction

Cytokinins were identified for their role in cytokinesis/cell division and are well-studied alongside with their alter-ego, auxins [1]. Due to the ubiquitous presence of cytokinin and auxin in different processes, it is extremely challenging, yet not entirely impossible, to peer directly into molecular signaling processes. Clever reporter techniques, like the DR5 and TCS reporter systems, as well as various studies that measure cytokinins and auxins directly or indirectly have given us insight about the interplay between auxin/cytokinin regulatory feedback loops that control plant stem cell pools [2]. Interestingly, cytokinins are synthesized and transported throughout the plant [3], such that the involvement of plant TCS in many processes is highly likely. Cytokinin signaling is found to be involved in auxin [4], ethylene [5], sugar and phytochrome B signaling [6], the circadian clock [7], lateral root formation [8], light [9], abscisic acid and cold [10], drought and freezing tolerance [11], meristem [12–14], female gametophyte development [15] and stomatal regulation [16]. Cross-talk studies have revealed intricate signaling pathways between auxin, cytokinin and ethylene [17].

The two-component phosphorelay system (TCS) is a signaling mechanism present in many organisms. Cytokinin signaling in plants is a well-described multi-step phosphorelay system (MSP) functioning as an H->D->H->D phosphorelay [3, 18]. It involves TCS phosphorylation-dependent interactions [19, 20] some of which are channeled into gene activation [3]. The classical TCS first observed in bacteria consists of two conserved proteins: namely a sensor histidine kinase (SK or HK) and a response regulator (RR). The HK responds to a ligand resulting in an H->D phosphorelay onto the Receiver domain (REC) of the RR, modulating RR activity [3, 21]. Plants contain hybrid-HKs with REC domains co-present with their HK domains [3, 22]. While the overwhelming majority of RRs have REC domains adjunct with transcriptional output domains (OUTs), there are also RRs that do not have OUTs and comprise the class of Single Domain RRs (SD-RRs) [23]. In plants, RRs with OUTs are called B-type (or type-B) RRs and those without A- and C-type (or type-A, -C) RRs [3]. For simplicity, we will refer to *Arabidopsis thaliana* response regulator (ARR) A-type RRs, B-type RRs or C-type RRs as A-type, B-type or C-types in this work.

Cytokinin in *A*. *thaliana* is perceived by three hybrid-histidine kinases, AHK2, AHK3 and AHK4 via their CHASE domain leading to their autophosphorylation on a conserved His residue in the Dimerization and Histidine phosphotransfer domain (DHp) whereupon the phosphoryl group is relayed onto a conserved Asp of the AHKs’ REC domain [3]. This phosphate residue, or TCS~P for short, is then relayed onto the conserved His residue of *A*. *thaliana* histidine phosphotransfer proteins (AHPs) which shuttle freely between the cytoplasmic and nuclear compartments irrespective of their TCS phosphorylation state [24]. Thereafter, TCS~P from AHPs is relayed onto the conserved Asp residue of an A-, B- or C-type RR, modulating their outputs [3, 6, 25–27]. For A-types, this determines how they interact with other proteins [6, 20, 28] and for B-types, TCS phosphorylation is thought to release the inhibitory function of the REC domain on the OUT domain [29, 30] enabling DNA binding and subsequent target-gene activation [3, 29]. B-type activation is therefore dependent on the phosphorelay and the availability of TCS~P, that is, AHPs phosphorylated on their conserved His. *A-types* are rapidly induced by the addition of exogenous cytokinin (e.g. *ARR5*, [31]) via a common core target sequence 5’-RGATY-3’and this transcriptional response is directly mediated by B-types [30, 32–35].

Of the eleven B-types in *A*. *thaliana*, 5 of the 7 subfamily B-1 members mediate cytokinin signaling, namely ARR1, ARR2, ARR10, ARR11 and ARR12 [36]. *ARR2* is known to be expressed ubiquitously in *A*. *thaliana*, albeit to different degrees [37–39] and not surprisingly it is involved in many aspect of plant development and regulation. ARR2 shows both positive and negative synergy with ARR1, ARR10 and ARR12 in root cytokinin signal transduction [36], is involved in senescence [40] and stomatal regulation [16], is also implicated in ethylene crosstalk [37], plant immunity [41] as well as positive feedback in the cytokinin/auxin feedback loops in the shoot meristem [14]. ARR2 is also involved in the onset of the endocycle in the root independently of ARR1 and ARR12 [42], yet ARR2 regulates *SHORT HYPOCOTYL 2*, along with ARR1 and ARR12 [42]. Overexpression of wild-type ARR2 has been shown to occasionally cause cytokinin hypersensitive responses [37, 43]. Experiments with transgenic plants have shown that ARR2 needs to transfer TCS~P on its conserved Asp residue in order to exert its overexpression effects *in planta* [40] and that phosphor-mimicry by converting D to E caused severe pleiotropic aberrations [37].

The *A*. *thaliana* C-types ARR22 and ARR24 have TCS-active REC domains which are more similar to the AHK REC domains [26, 44]. Interestingly, no physiological function has so far been ascribable to either [26, 45, 46] although ARR22 is a functional RR expressed at the chalaza/funiculus junction in *A*. *thaliana* [45, 46]. So far, every study conducted could not expose any effect for loss of *ARR22* at the phenotypic, molecular or metabolic levels [11, 26, 45, 46]. The transcription of *ARR22*, like B-types, is not regulated by cytokinin [26, 47, 48]. Overexpression of ARR22 however has been shown to result in dwarfed plants and poor developed root systems phenocopying the *wooden leg* (*cre1/wol/ahk4*) mutant [26]. Even minute amounts of mis-expressed ARR22 led to pleiotropic developmental disturbances of variable penetrance [46]. These effects have been traced to dominant negative effects ARR22 exerts on TCS signaling [26, 45, 46]. ARR22 has been shown to have a high TCS~P autodephoshorylation rate *in vitro* and is thought to function as a phosphohistidine-phosphatase [26, 45, 46, 49] as it has an autodephosphorylation rate [26] that is more rapid than that observed for A-types ARR3 and ARR4 [6, 27], the B-type ARR11 [50] or AHPs [6, 27, 50]. Its catalytic ability requires the conserved Asp in the RR domain, and mutation of D->N or D->E results in complete loss of function *in vitro* [26] and more importantly, also *in vivo* [11, 26, 45, 46, 49]. Unlike A-types that function within and outside the TCS phosphorelay via interaction with other proteins dependent on their TCS phosphorylation state [6, 20, 28], mutation of the conserved phosphorylated Asp in ARR22 has not caused any auxiliary physiological phenotypes [26, 45, 46].

In this study, we explore TCS~P flow by examining the overexpression phosphocompetition effects of ARR22 alongside other TCS signaling pathway components utilizing our previously developed *in vivo ARR5p*::*LUCm*^*3*^ firefly luciferase (fLUC) reporter system [49]. *ARR5* is a well-characterized A-type gene known to be induced by cytokinin by being directly bound and activated by various B-types [2, 32, 34, 51–54]. Four novel complementary metrics were derived to interpret the fLUC-derived reporter activity. In contrast to an *in vitro* system, the *in vivo* system is the sum of the phosphorelay and partially undescribed interaction networks. Our data show that ARR22 overexpression can outcompete overexpression effects of B-types and AHPs, and is more potent at hampering the phosphorelay compared to A-type overexpression when excess B-type is present in the cell.

## Results and discussion

### Experimental set-up and data extraction

**Fig 1:**
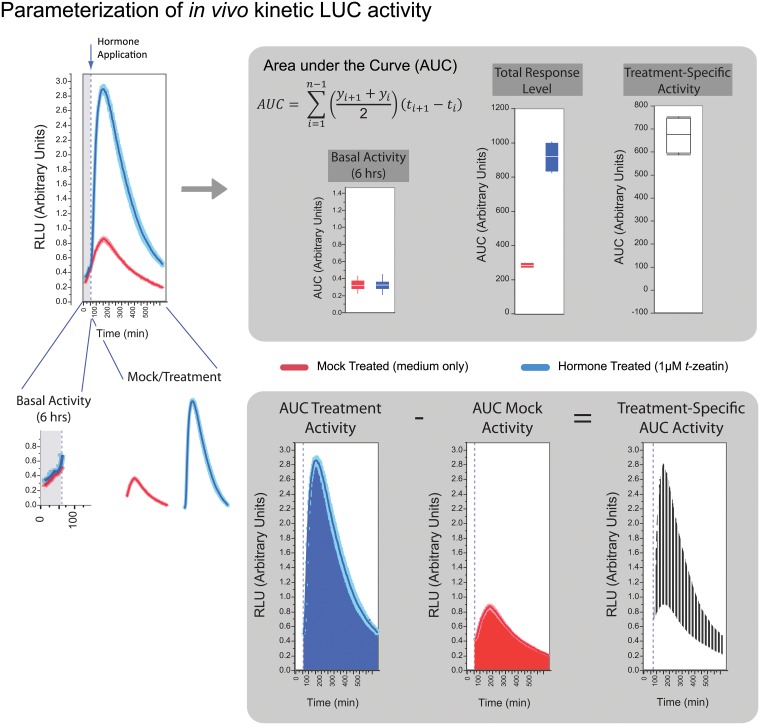
Graphical summary of the parameters extracted from the light emission curves. Transcriptional activity of a *promoter*::*fLUC* construct was quantified in a plate reader every 5 minutes as light emission from oxidative decarboxylation of D-luciferin by firefly luciferase (fLUC). The light emission is represented in Relative Light Units (RLU). The total luciferase activity was estimated by calculating the Area under the Curve (AUC). The AUC from the first hour, captures the Basal Activity, which is promoter-reporter expression before stimulation. The Basal Activity values enable controlling transfection and technical variance as well as assessing any treatment-independent, effector specific effects. After one hour, hormone or only medium was added to each respective half and LUC emission monitored for 10 more hours. The Mock and Treated sample Total Response Levels correspond to the AUC excluding Basal Activity. After blocking and ranking, the AUC of Treated minus Mock total response levels was calculated in a pair-wise manner deriving the Treatment-Specific Activity. The dark contour lines represent the mean of 4 to 6 replicates, red being mock and blue being *t*-zeatin treated. See the Methods for more details.

**Fig 2:**
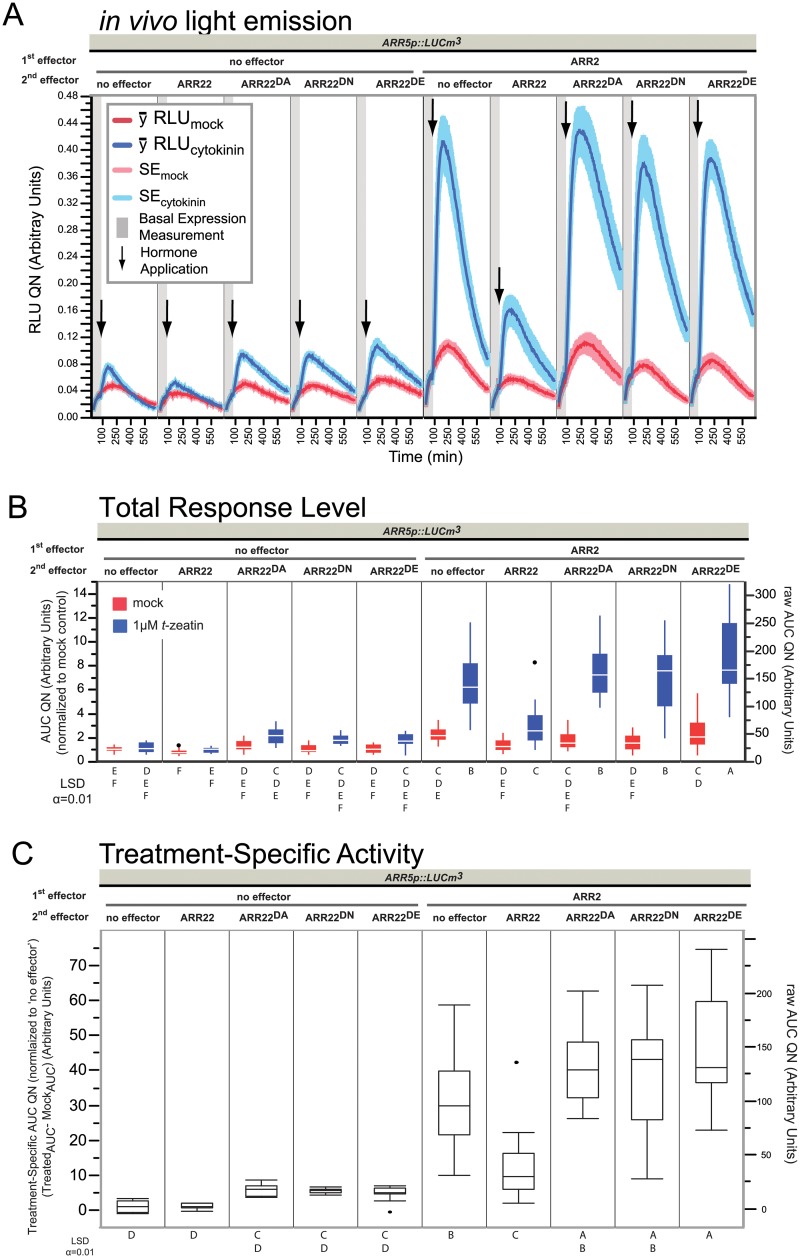
Reporter gene activity directly reflects phosphorelay action *in vivo*. (A) *in vivo* light emission curves obtained over 11 hours. Data is composed of four independent experiments, each containing 4 replicates per sample type. The experiments were combined after quantile normalization (QN, see Methods, S26 Fig). One replicate of this dataset with only ARR22 variants using a different metric was published in [49]. (B) AUC calculated after excluding the first hour, that is, beginning after treatment. (C) After ranking (see Methods), the AUC_cyt_−AUC_mock_ was calculated for all four sets, which gives the area corresponding to the space above the mock curves and bordered by the cytokinin treated curves. Significance classes were determined using Fischer’s Least Significant Difference test, α = 0.01. Letters not connected by a letter are significantly different. 2 μg plasmid was used for each effector in 30 μL transfections at 6.6x10^6^ protoplasts mL^-1^.

TCS input and output activity can be monitored by using a promoter-reporter system that is tuned to the TCS. This means that one can study phosphocompetition *in vivo* by monitoring the gene transcription of cytokinin-responsive genes. In this case, a reporter construct encoding the non-coding, upstream intergenic region of *ARR5* was cloned upstream of firefly Luciferase *LUCm3*. We previously showed that overexpression of the C-type ARR22 prevented the activation of our LUC reporter [49] consistent with that of transgenic studies [26]. We have expanded that methodology by deriving four metrics to capture the amount of cytokinin dependent and independent activity. We continually monitored the transcriptional activity of *ARR5p*::*LUCm*^*3*^ in the same cells over the experimental time period by measuring the LUC activity in Relative Light Units (RLU) and calculated the Area under the Curve (AUC) (Fig 1). The AUC takes into account the time between measurements and gives numerical values for testing differences via statistical procedures. After transfection, the cells were incubated for six hours before adding the fLUC substrate D-luciferin. Thereafter the light emission was monitored from living cells in a microplate reader every five minutes for one hour. This captured both basal overexpression effects and replicate efficacy before experimental treatments (see Fig 1: Basal Activity). Thereafter, a treatment was applied as mock (medium only) or cytokinin (to a final concentration 1 μM *t*-zeatin in medium) directly to the protoplasts and continually monitored every five minutes for ten more hours. Instead of taking a peak expression approach (see [49]), the total transcriptional activity over the measurement interval includes all kinetic and subtle differences that are present in the dataset, called the Total Response Level (Fig 1: Total Response Level). From this, treatment specific effects due only to cytokinin are derived by subtracting the mock AUC from the treatment derived AUC and is termed Treatment Specific Activity (Fig 1: Treatment Specific Activity), giving a more exact amount activity ascribable to treatment effects only, here the cytokinin *t*-zeatin. For example from Fig 2B, if we took the ratio of only ARR2-overepxression (ARR2-ox) to that of the promoter alone at the Total Response Level, we obtain a circa 3-fold induction level (~150/~50 = ~3). Yet, the quantification of the Treatment Specific Activity reveals that actually approximately 30-fold more cytokinin-specific activity (~90/~3 = ~30) was produced over that of the promoter alone, whereby the cytokinin Treatment Specific Activity of the promoter alone was set to 1 (Fig 2C). To simplify these types of comparisons, the experimental graphs retain the raw AUC scale on the right-side axis as well as the same values referenced to the promoter alone mock control on the left-side axis. In general transfection variance could be adequately controlled allowing representative experiments to be shown (see Methods). Where overall transfection levels still varied, but the biological readout was similar, the data could be normalized using the method of quantile normalization (QN). Where doubt was still present in transfection variance, *pCF203* encoding *GREEN FLUORESCENT PROTEIN* (*GFP*) was co-transfected and the GFP intensity was quantified and the RLU emission rescaled according to the GFP emission level. Where needed, these techniques were combined. Comparative and exploratory data are described in the Methods.

### ARR22 can block the transactivation and cytokinin-dependent activation of ARR2

**Fig 3:**
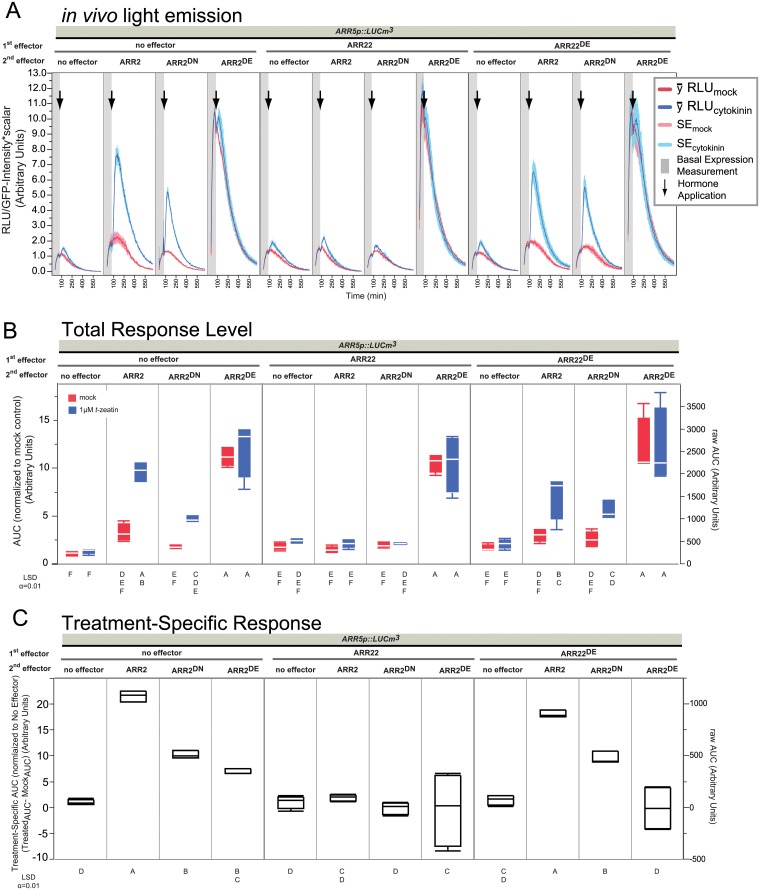
Transactivation potential of various mutations of the phosphorylated Asp in ARR2. Panels are the same as in Fig 2 with the following changes: Data is shown for one experiment, each containing 4 replicates per sample type. RLU is relative to the GFP emission level (see Methods, S27 Fig). 2 μg plasmid was used for each effector in 60 μL transfections at 3.5x10^6^ protoplasts mL^-1^.

**Fig 4:**
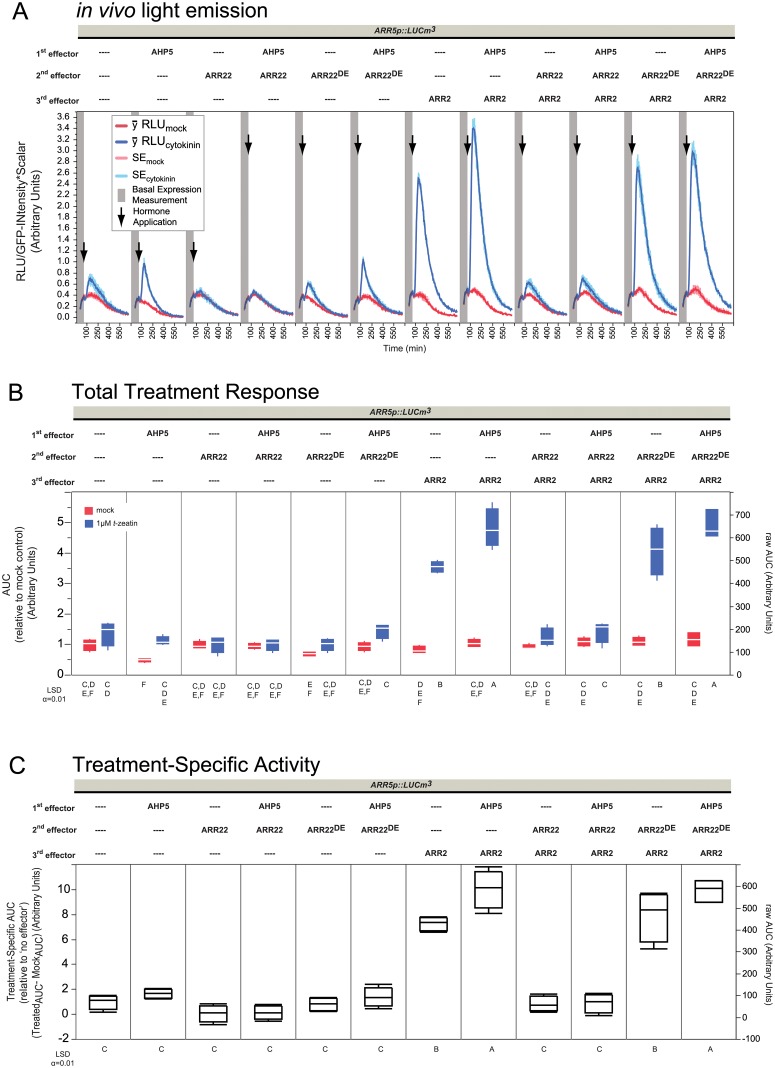
Overexpression challenge of AHP5 against ARR22. Panels are the same as in Fig 2 with the following changes: Data is shown for one experiment, each containing 4 replicates per sample type. 2 μg plasmid was used for each effector in 60 μL transfections at 3.5x10^6^ protoplasts mL^-1^.

**Fig 5:**
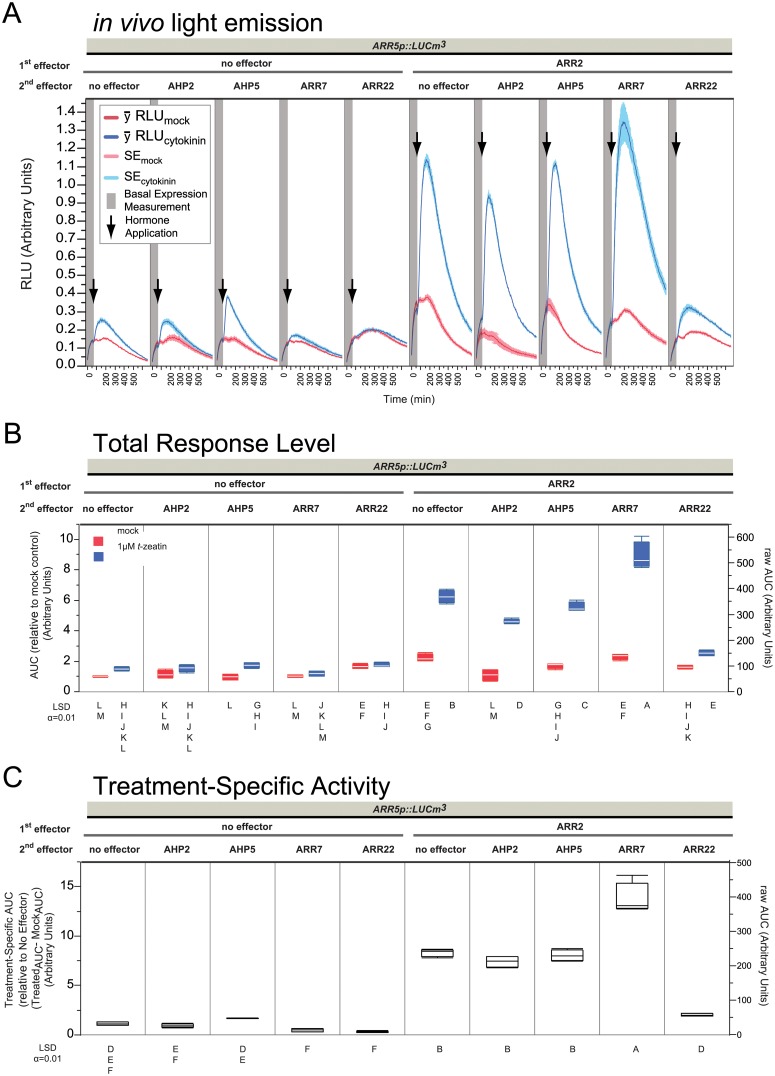
Overexpression comparison of AHPs, A-types, and ARR22 against ARR2 on *ARR5p*. Panels are the same as in Fig 2 with the following changes: Data is shown for one experiment, each containing 4 replicates per sample type. 2 μg plasmid was used for each effector in 30 μL transfections at 6.6x10^6^ protoplasts mL^-1^.

**Fig 6:**
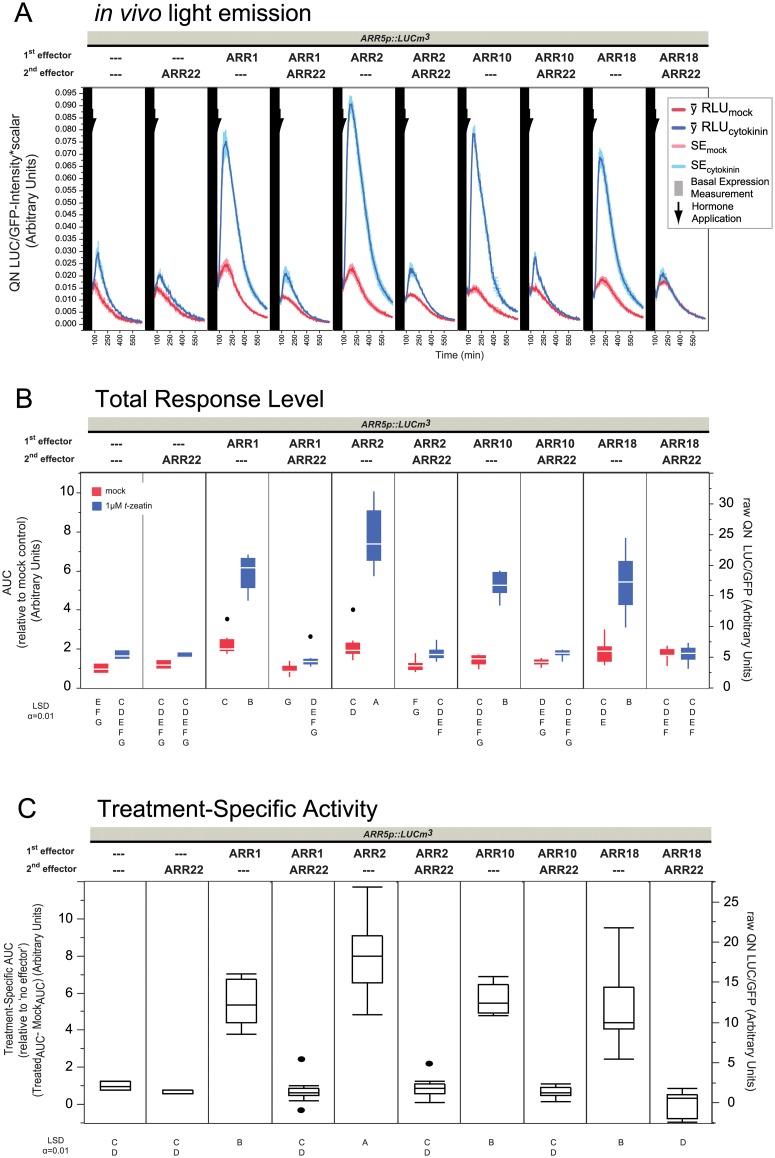
Action of ARR22 is upstream of B-types. Panels are the same as in Fig 2 with the following changes: Data is composed of four independent experiments, each containing 4 replicates per sample type. RLU is relative to the GFP emission. The experiments were combined after quantile normalization (QN, see Methods). 2 μg was used for each effector in 60 μL transfections at 3.5x10^6^ protoplasts mL^-1^.

**Fig 7:**
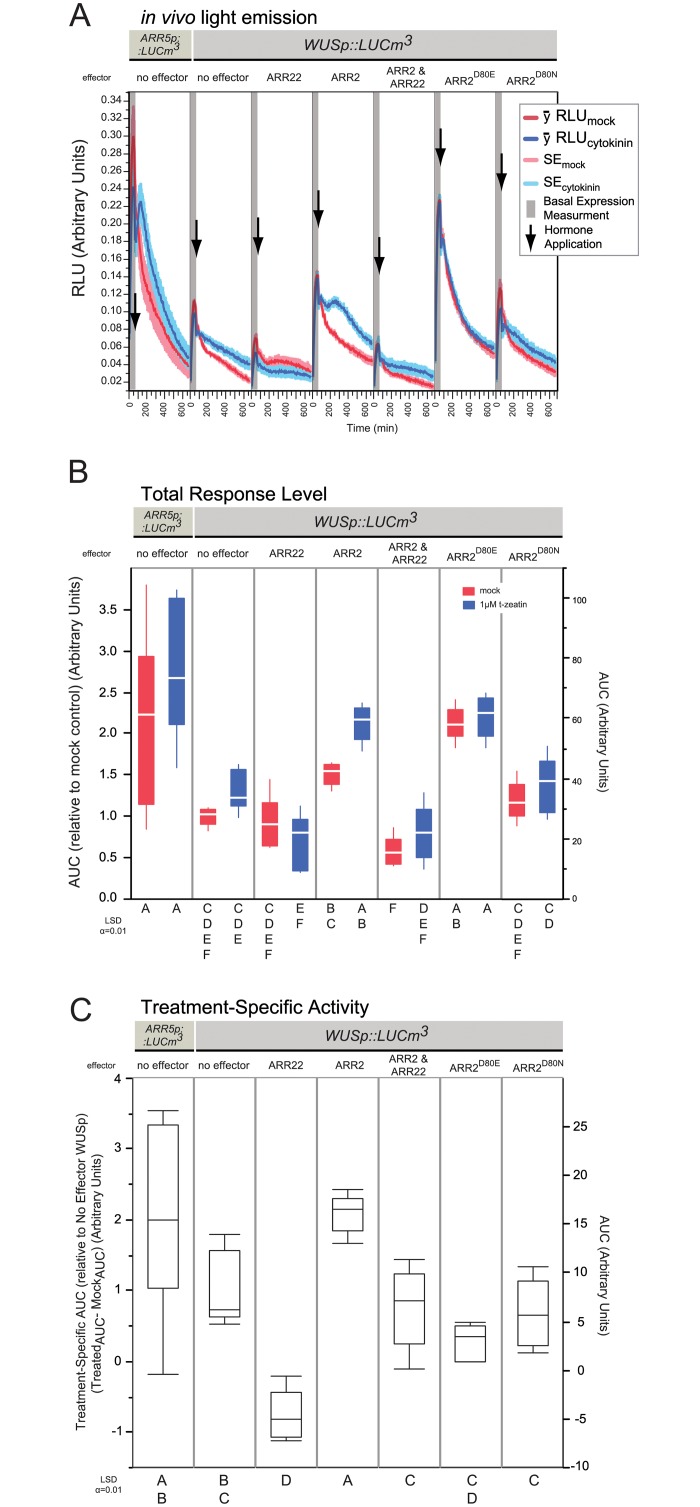
Transactivation of ARR2 on *WUS*. Panels are the same as in Fig 2 with the following changes: Data is composed of one experiment, containing 6 replicates per sample type. 2 μg was used for each effector in 30 μL transfections at 6.6x10^6^ protoplasts mL^-1^.

In our previous report, overexpression of ARR22^WT^ hampered the cytokinin response of *ARR5p*::*LUCm3* and this was dependent on the ability of ARR22^WT^ to accept TCS-phosphate [49]. In cytokinin treatments of whole plants or leaf material, *ARR5* has a peak expression level 20–40 minutes after cytokinin treatment [31], whereas here, the firefly LUC reporter gene *ARR5p*::*mLUCm*^*3*^ had a peak emission level shift of about 1 hour after cytokinin treatment, which we presume reflects the lag in protein synthesis (Fig 2A). Conceptually, ARR22^WT^ is proposed to effectively dephosphorylate AHPs before B-types have a chance to obtain TCS~P from them [26, 45, 46, 49], therefore we asked the question what happens when we overexpress a B-type ARR, like ARR2, simultaneously along with ARR22^WT^. To do this, ARR22^WT^ versions with mutations of its conserved phosphorylatable Asp74 to Ala, Asn or Glu (ARR22^D74N,A,E^) were transfected alone or along with ARR2 (Fig 2). As can be seen in Fig 2, two immediate observations can be made: one, overexpression of ARR2 resulted in a major increase in the cytokinin responsiveness of the reporter and two, ARR22^WT^ was able to not only block the endogenous B-types but also overexpressed ARR2 (Fig 2A: ARR2 with ARR22^WT^ compared to ARR2 alone), with both proteins stable under these conditions (S1 Fig). Consistent with previous results [26, 45, 46, 49], this effect of ARR22^WT^ was dependent on the conserved Asp of the REC domain as mutation to any other residue prevented the dominant-negative phosphocompetition effects (Fig 2A). The Total Response Level (Fig 2B) thus follows and supports the curve data (Fig 2A) providing the desired statistical measure. Even though cytokinin-dependent activity was present and measureable (Fig 2A and 2C), quantification of the exogenous effect of cytokinin on the promoter alone at this stringent level did not show a mathematically significant difference (Fig 2B: promoter alone vs. promoter alone + *t*-zeatin) as the data are not noise free. We felt it was important to leave the biological and experimental noise in our results and therefore a stringent statistical quantification cutoff α = 0.01 was chosen in order to focus on the major effects, considering that the reporter clearly and reproducibly responded to cytokinin and dramatically, in a cytokinin-dependent manner with additional B-type ARR2 (see Figs 2–7).

Quantification of the curve data by the AUC metrics (Fig 2B and 2C, S2 Fig) supports the visual interpretation of Fig 2A. ARR2-ox has repeatedly been reported to be transactive on its own, although the fold-levels vary from one experimental set up to another [33, 37, 40]. We typically observed that ARR2-ox was transactive with ~2-fold higher level already at the Basal Level (S2 Fig). This was also preserved at the Total Treatment Response in the mock treated samples (Fig 2B: ARR2-ox alone, mock). However, we observe that ARR2 is principally TCS dependent as it was more transactive when the TCS was activated by cytokinin, in this case ~30-fold over the promoter alone (Fig 2C). Both mock and cytokinin induction effects of ARR2-ox were blocked by ARR22^WT^-ox (Fig 2B and 2C) whereas ARR22^D74N,A,E^ variants led to the same or only slightly higher Treatment Specific Activity (Fig 2C). This indicates that ARR22 does not negatively regulate the TCS independently of its phosphohistidine phosphatase function, consistent with transgenic studies over- and mis-expressing ARR22 [26, 45, 46]. Additionally, differences between ARR22^WT^ and its variants were not due to basal effects (S2 Fig). Interestingly ARR2 basal transactivity also might be TCS dependent as co-expression with ARR22^WT^ reduced ARR2-ox mock levels to that of the promoter alone (Fig 2B). Thus from our data, the TCS dependency of ARR2 is consistent with those studies which did not perturb development or response to cytokinin when ARR2 was overexpressed [37, 40] and is in contrast to other studies which did [33, 43, 55]. Furthermore, the high cytokinin response observed when ARR2 was overexpressed indicates that the endogenous B-type levels must be limiting and that there is more AHP~P likely present than endogenous B-types to receive it. Likewise, overexpression of ARR22^WT^ can outcompete an overexpressed B-type ARR2 for TCS~P, as we observed a reduction of ~30-fold (ARR2 alone) to ~10-fold (ARR2 + ARR22^WT^) treatment specific activity. Thus ARR22^WT^-ox likely hampers ARR2-ox from obtaining TCS~P from AHPs due to its phosphohistidine phosphatase effect which is lost when the conserved phosphorylatable Asp is mutated (this work, [26, 45, 46, 49]). However, in order to make this statement, one has to confirm that ARR2 activation is TCS dependent. Our data indicates that although ARR2 is transactive as previously observed [30, 33], it is clearly much more dependent on the TCS for its full-activation in a cytokinin-dependent manner. Nevertheless, Hwang & Sheen, 2001 proposed that ARR2 was not (strictly) TCS dependent as a mutation in the phosphorelay-Asp residue, ARR2^D80N^, only led to a moderately reduced and not inert ARR2 variant. Transgenic studies using ARR2^D80N^ however suggested the opposite, as ARR2^D80N^ plants *did not* lead to a cytokinin-dependent delay of leaf senescence compared to the overexpression of the wild-type protein [40]. Therefore based on the previous results and the current understanding of RR function, we set-up a phosphocompetition experiment of ARR22 with ARR2^D80N^ to explore if ARR2 is TCS dependent or not.

### Transactivity of the non-phosphorylatable ARR2 variant ARR2^D80N^ is TCS dependent

The B-type REC domain exerts a negative regulation on the DNA binding and transactivation ability [30]. This negative regulatory function is modulated by the phosphorylation status of a conserved Asp (D) in the catalytic acidic pocket of the REC domain ([3, 21, 56], S3 Fig). D->E substitutions in ARR2 result in a constitutive active protein [37] whereby D->N leads to a defective, but not null-protein [33, 40]. Neither ARR2^D80E^ nor ARR2^D80N^ is phosphorylated in a TCS dependent manner [37, 40], demonstrating that Asp80 is the critical Asp for ARR2. These two mutations are routinely used to demonstrate the TCS dependency of ARR2 [33, 37, 40, 55] and therefore the corresponding point mutations were also introduced into ARR2. These cDNA variants of *ARR2* were transfected along with *ARR5p*::*LUCm3* and challenged by co-expression with ARR22 or its inactive variant ARR22^D74E^ (see Fig 3).

ARR2-ox led to a much stronger reporter activity in the LUC emission curves (Fig 3A) and when quantified at the total levels (Fig 3B) is consistent with that shown in Fig 2. In comparison, this dataset produced a 20-fold more cytokinin specific activity over that of the promoter alone (Fig 3C) and this was counteracted by ARR22^WT^ co-overexpression but not ARR22^D74E^ (Fig 3). Before treatment, ARR2^D80E^-ox was ~10x over mock (S4 Fig), demonstrating that the D->E substitution releases REC inhibition on transactivation capability, congruent with previous observations [33, 37, 40, 57]. This high level of expression was maintained during mock and cytokinin treatments (Fig 3B) and was principally independent of cytokinin, as ARR2^D80E^ only produced about 5-fold more cytokinin-specific activity when expressed alone (Fig 3C). Thus, overexpression of ARR2^D80E^ resulted in high LUC activity which nearly maximized during the basal measurement, and then stayed high, decaying slowly over time (Fig 3A). To investigate why the ARR2^D80E^ light emission curves continually decayed over time, the light emission pattern of ARR2^D80E^ was compared to that of a known constitutive promoter *UBI4/2* [58]. As shown in S5 Fig, the expression profile of ARR2^D80E^ is similar to that of *UBI4/2*::*LUCm3* and indicative of constitutive (high-transcriptional rate) promoter activity. The variant ARR2^D80E^ theoretically should be able to bypass ARR22 as ARR2^D80E^ *does not need* to obtain TCS~P from the AHP~P pool for its activity. This was confirmed since neither overexpression of ARR22^WT^, ARR22^D74E^ nor ARR22^D74A^ were able to prevent the 10-fold transcriptional output of ARR2^D80E^ (Fig 3B, S6 Fig). The cytokinin-dependent activity of ARR2^D80E^ when overexpressed alone is likely due to the endogenous B-types present in the cell. We conclude that this cytokinin-dependent activity can be occasionally observed for ARR2^D80E^-ox from time to time but varies (Fig 3, S5–S7 Figs). More importantly, ARR2^D80E^ led to high constitutive activity in all conditions confirming that ARR2^D80E^ is able to bypass the TCS activation requirement.

In contrast to observations in previous studies on the *ARR6* promoter in mesophyll protoplasts [33, 40], in our assays ARR2^D80N^-ox generally showed comparable levels to that of the promoter alone at the basal and mock levels (S4 Fig, Fig 3B). All of the ARR2 REC mutants were detectable by immunoblot analysis (S8 Fig). In the presence of cytokinin ARR2^D80N^-ox resulted in a significant enhancement of cytokinin response that was weaker and dampened quicker than that of ARR2 (Fig 3A). This is analogous with previous reports where the D->N substitution led to reduced protein activity seen in transactivity or cytokinin-dependent inductive responses [33, 40, 57]. ARR2^D80N^ overexpression led to circa 9x more cytokinin specific-activity over the promoter alone, which corresponds to almost 50% of the ARR2^WT^ protein (Fig 3C). Unlike ARR2^D80E^ however, the cytokinin dependency was inhibited by ARR22^WT^ (Fig 3) but not variant ARR22^D74E^, which was completely inert, even allowing the same specific-activity as that of ARR2^D80N^ alone (Fig 3C).

There are three conserved negatively charged amino acids of the REC domain that comprise the catalytic acidic pocket, with two of those being absolutely conserved Asp residues ([56], S3 Fig). Mutation of the two absolutely conserved Asp to a non-phosphorylatable residue prevents aspartyl-phosphate formation, whereas the third residue of the acidic pocket must be an Asp or Glu [30, 56]. That overexpression of ARR22 blocks the cytokinin induction of ARR2^D80N^ (Fig 3) suggests that either ARR2^D80N^ is still phosphorylatable or that it might interact with a component targeted by ARR22 needed for ARR2 to be transcriptionally active. In that regard, it is interesting that another B-type variant ARR18^D70N^ is also weakly transactive [57]. Based on work in bacteria, disturbance of the catalytic pocket does *not* always result in a completely dysfunctional response regulator protein [59, 60]. It is known that mutation of the Asp phosphorylation site can also led to aberrant phosphorylation and not always a null mutant, even if Asn or Glu are not phosphorylated [19, 56, 59]. Furthermore, constitutive active versions of the bacterial RRs CheY mutation E9Q and PhoB D13K were not mutations of the actual phosphorylated Asp [59, 60]. The acidic pocket of ARR2 is conserved with CheY and PhoB (S3 Fig) and such complex effects involving ARR2^D80N^ could also be occurring. Either way, these comparisons of ARR2 D80 variants clearly indicate that ARR2 is activated in a TCS-dependent manner. And, as suggested before, that ARR22 functions within the TCS in a dominant-negative fashion preventing ARR2 TCS-dependent activation. Taken together we can establish that ARR2 and ARR22 work in a TCS-dependent manner with respect to *ARR5p*::*LUCm3* activation (Figs 2 and 3).

### Overexpression of AHP5 cannot outcompete ARR22 overexpression

In the previous experiments we established that ARR22^WT^-ox is likely able to deplete AHP~Ps even when ARR2 is overexpressed, reducing the output partially (Fig 2C) or completely (Fig 3C). We postulated that if ARR22^WT^ is controlling the rate-limiting access to AHP~Ps, perhaps overexpression of an AHP would be able to counterbalance ARR22^WT^ effects. AHP1, 2, 3, and 5 are found in the cytoplasm and the nucleus (AHP4 was reported only cytoplasmic [61]) and shuttle freely between the two compartments irrespective of their TCS phosphorylation state [24]. ARR22 is also located in cytoplasm and nucleus [26, 46] where it was also observed to interact with AHP2, 3, and 5 [46]. As mentioned, ARR22 is able to remove ~P from AHP5 *in vitro* [26] and likely *in vivo* [26, 45, 46, 49]. Therefore, a competition experiment was designed combining AHP5 with ARR22 or ARR22^D74E^ and ARR2 (Fig 4).

As shown before, ARR2-ox led to a large boost to cytokinin responsiveness of the reporter (Fig 4A) quantified here to about 7x more specific-activity (Fig 4C) fitting with the slightly lower overall basal levels compared to the previous experiments (S2, S4 and S9 Figs). Nevertheless the biological responses are consistent with the experiments shown before (Figs 2 and 3). Also as already described, ARR22^WT^ prevented ARR2 cytokinin-responsiveness and ARR22^D74E^ did not (Fig 4). Overexpression of AHP5 fostered a bit more reporter output at the endogenous B-type levels (Fig 4A) even being significantly different at the total response level due to an overall lower output at the mock level (Fig 4B) but not at the treatment specific-activity level, even though it was slightly higher than the promoter alone (Fig 4B and 4C). Since AHP5 was stably expressed (S10 Fig), this indicates that AHP5 is generally not limiting with respect to the cytokinin-dependent promoter activity in contrast to that observed by the overexpression of B-type ARR2. Thus, endogenous levels of B-types must be much less compared to the availability of AHP~Ps during cytokinin signaling. This positive effect of AHP5 under cytokinin treatment was preserved in the presence of ARR2 in these experiments (Fig 4B); here, co-overexpression of AHP5 and ARR2 significantly increased the cytokinin specific-activity (Fig 4C). Nevertheless, overexpression of ARR22^WT^ prevented ARR2 cytokinin-responsiveness at both the endogenous B-type level and when ARR2 was also co-overexpressed with AHP5, reducing the cytokinin specific-activity to that of the promoter alone (Fig 4C). Thus, although AHP5 had a slight positive effect on the reporter activity, its overexpression was not able to compensate for the dominant-negative phosphohistidine phosphatase activity of ARR22^WT^. As observed in the previous experiments, ARR22^D74E^ did not alter the overexpression effects of AHP5, ARR2 or their co-expression (Fig 4) reaffirming that ARR22 functions strictly on TCS signaling.

### Overexpression of neither A-types nor AHPs have the same rate-limiting effect as ARR22 on ectopic B-type levels

Previous studies showed that overexpression of AHP2 or AHP5 did not affect an *ARR6*::*LUC* reporter in mesophyll protoplasts irrespective of cytokinin [33] even though ectopic expression of AHP2 in plants resulted in cytokinin hypersensitivity [62]. Therefore a comparative phosphocompetition experiment was conducted in order to ascertain the effects of AHP2-ox in our biological system. Based on what is known about the TCS, A-types negatively regulate the TCS and their genes are induced by B-types as a negative-feedback mechanism [20, 53, 63]. This negative feedback is partially dependent on their ability to pick up TCS~P from AHPs as mutation of their conserved phosphorylatable Asp to another residue abolishes phosphorelay function *in vitro* [19] and *in vivo* [19, 20]. Therefore we asked if A-type overexpression could prevent ARR2 TCS-dependent activation as that observed for ARR22.

Inspection of Fig 5A reveals comparable results to those previously described for ARR2-ox and ARR22^WT^-ox in this dataset. ARR2-ox led to about 2-fold more activity at the basal (S11 Fig) and mock levels (Fig 5B) whereby cytokinin treatment ushered in ~7.5x more specific activity (Fig 5C). Likewise, ARR22^WT^ prevented cytokinin responses at endogenous B-type and ARR2-ox levels (Fig 5A–5C). At the endogenous B-type levels, overexpression of AHP5 led to a visible increase in reporter activity (Fig 5A) of which significant differences could be ascertained at the total response level (Fig 5B) but not at the treatment specific level (Fig 5C). This is similar to that observed in the previous experiment (Fig 4). In contrast, in conjunction with ARR2, AHP5-ox and AHP2-ox lowered the basal and mock expression effects of ARR2-ox in Fig 5B. This effect was not observed in Fig 4, likely due to the lower overall basal expression in Fig 4 since both proteins were stably expressed (S10 Fig). More importantly, neither AHP2 nor AHP5 affected ARR2 specific activity (Fig 5C), indicating that AHP2 is not limiting and that AHP5 can cause cytokinin hypersensitivity under the right conditions (Fig 4), yet even when this effect is minor compared to overexpressing ARR2 with respect to gene activation.

A-type dominant-negative effects on the TCS due to overexpression are typically only observed when exogenous cytokinin is applied, and thus, without applying cytokinin appear like wild-type both phenotypically and at the molecular level [19, 20, 63]. By this accord, overexpression of A-types is predicted to block or dampen the reporter gene *ARR5p*::*LUCm3* in a cytokinin dependent manner. While ARR7-ox lowered the promoter activity non-significantly at the basal and specific activity levels (S11 Fig, Fig 5C), it clearly prevented cytokinin induction at the endogenous levels similar to ARR22 (Fig 5A: ARR7-ox alone). Quantification of the AUC values confirmed a loss of induction at the total response level (Fig 5B) and the treatment specific response (Fig 5C), significantly for the former. Inspection at the basal levels (S11 Fig) suggests that without cytokinin, ARR7 was able to inhibit ARR2-ox to about 50% of its transactivity. Unlike ARR22^WT^-ox however, under cytokinin treatment, overexpression of ARR7 with ARR2 was not able to prevent ARR2 from obtaining TCS~P and in this case resulted in surprisingly more treatment-specific activity (Fig 5C). This prompted testing two other A-types: ARR4 and ARR15. In addition, the conserved phosphorylatable Asp95 of ARR4 was mutated to ARR4^D95E^ or ARR4^D95N^ (S12 Fig).

As observed for ARR7, when expressed alone overexpression of ARR4 and ARR15 also prevented cytokinin induction of *ARR5p*::*LUCm3* (Fig 5, S12 Fig). Still, while ARR4, ARR7 and ARR15 could block the endogenous driven reporter activity, they were not able to prevent ARR2-ox effects (S12 Fig) even though all proteins could be confirmed by immunoblotting (S13 Fig). The positive-induction response of ARR7-ox alongside ARR2-ox (Fig 5) was not reproducible (S12 Fig) and in general there was more variability in the A-types samples co-transfected with ARR2 such that negative, positive and neutral effects were observed (S12 Fig). A-types are regulated in very complex manners [3] such that it is possible that minor environmental differences compounded output effects when ARR2 was co-overexpressed. The mutations ARR4^D95E^ and ARR4^D95N^ abolished the ability of ARR4 to inhibit the endogenous cytokinin induction, yet had no dominant negative effect on ARR2-ox (S12 Fig). Remarkably, the reporter activity for ARR4^D95N^ and ARR4^D95E^ overexpression is very similar to that observed for ARR7^D85N^ transgenic plants [19], whereby the D mutations led to a reduced, but steady and sustained induction level over the measurement timespan compared to the promoter alone (S12 Fig). This demonstrated that our experimental conditions match results obtained with A-type ectopic expression in plants [19, 20] previously thought not possible. One interpretation is that A-types can hold certain concentration of B-types at a limiting equilibrium, and also implies that some B-types are still able to obtain TCS~P even when A-types are present. When cytokinin is given exogenously and drives the TCS in the forward direction, an excess of A-types can prevent the excess TCS signal from reaching endogenous level B-types, seen here as a loss of cytokinin-dependent reporter activity. In conclusion, none of the A-types were able to prevent significant amounts of TCS~P from reaching overexpressed ARR2 like that observed for ARR22^WT^-ox.

### Various B-types are equally perturbed by ARR22 overexpression

ARR22 therefore functions upstream of ARR2 at the AHP/A-type phosphorelay level (Figs 4 and 5, S12 Fig) and its dominant-negative effect could not be compensated for by AHP5-ox or AHP2-ox (Figs 4 and 5). Furthermore, A-types were only able to block TCS information flow dictated by the B-type concentration levels (Fig 5) since we observed that the ARR2 level was principally the rate-limiting step in gene activation and not the concentration of the AHPs or A-types (Figs 2–5). Taken together, ARR22 is therefore most likely preventing B-types from receiving the phosphorelay signal via AHPs at a rate significantly faster than that of A-types. If this is so, ARR22-ox should generally knock out the TCS and also prevent other B-types in a TCS-dependent manner. Alternatively, the effect of ARR22 could be directed somehow only to specific B-types, even indirectly through AHP specificity. Therefore three additional B1-subfamily B-types ARR1, ARR10 and ARR18 were cloned into to the same effector vector and challenged against ARR22 (Fig 6). All of these B-types have been shown to target *ARR5* and/or *ARR6* in various studies [33, 37, 51, 57, 64] and all can interact with at least 2 of the 3 AHPs known to interact with ARR22 (summarized in S14 Fig as a Cytoscape graph).

Even though basal levels (S15 Fig) were a bit lower, the experimental output is comparable to the previous experiments as judged by inspecting the promoter alone, ARR2, ARR22^WT^ and ARR2 & ARR22^WT^ (Fig 6). All of the B-types were stably expressed (S16 Fig). Similarly as described in previous experiments, ARR2 overexpression led to ~8x more specific activity (Fig 6C), matching the levels obtained in Figs 4 and 5. As seen in the total response levels, ARR2 led to ~8x more specific activity compared to ~5x for ARR1 (Fig 6A), reminiscent of ARR2 being more transactive and/or responsive to cytokinin than ARR1 in general [30, 33]. Thus, the higher transactivity of ARR2 and ARR1 was confirmed in this study at the total response mock level (Fig 6B) [30, 33]. ARR10-ox and ARR18-ox on the other hand did not perturb the mock levels much (Fig 6B). These results are consistent with a report that found ARR1 and ARR2 more transactive than ARR10 in mesophyll protoplasts [33]. ARR10 and ARR18 showed comparable specific activity as that of ARR1 (Fig 6C), which is analogous to the same study in mesophyll protoplasts, where ARR10 was shown to have low transactivity but high cytokinin responsivity [33]. Inspection of the literature showed that ARR18 led to more cytokinin-responsive activity than ARR2 on an *ARR5p*::GUS reporter assay [57] yet less than ARR1 when tagged as c-myc-ARR18, and yet greater than ARR1 when ARR18 was not epitope tagged [64]. Thus differences in protein output and stability therefore can be traced to epitope tagging of B-types [43, 55, 64, 65] and apparently, the position and the type of epitope tag (or lack thereof) might affect B-type signaling capacity. That said, in our experiments, all of these B-types were able to activate the *ARR5p*::*LUCm3* reporter in a cytokinin-dependent manner (Fig 6) confirming the hypothesis that endogenous B-type levels are limiting.

Co-overexpression of ARR22^WT^ with ARR1, ARR2, ARR10 and ARR18 blocked their cytokinin-dependent induction effects (Fig 6). ARR22^WT^ typically did not absolutely eliminate all of the cytokinin-dependent activity when expressed alone in combination with the ARR2 or the other B-types (ARR18 being the exception) which could be explained as coming from the phosphorelay mediated by the AHPs AHP1 and AHP4 that are not targeted by ARR22, or just that the B-types are still able to find a phosphorylated AHP before ARR22 does. As ARR22^D74E^ (S17 Fig) did not exert the dominant-negative effect of ARR22^WT^ on these B-types, we can conclude that the phosphohistidine phosphatase activity of ARR22 is sufficiently rapid to severely dampen gene output that is dependent on the TCS. In conclusion, it appears that over/mis-expression of ARR22 in any environment is likely to lead to a severe and non-discriminatory reduction of B-type output.

### Combined ARR2 and ARR22 overexpression can determine TCS dependency of difficult targets

The various mutant variants of ARR2 and ARR22 can be used to determine if a promoter is a target of the cytokinin-mediated TCS signaling pathway or not. Therefore a recently identified, notoriously difficult target, *WUSCHEL* (*WUS*) was tested with this method. B-types ARR1, ARR2, ARR10 and ARR12 have recently been shown to directly bind to *WUS in vivo* [12–14] fulfilling a long standing prediction [66] concerning the complex regulation of the meristem’s organizing center [1, 66–68]. Therefore, the intergenic promoter region (-1741 to +1) of *WUS* was cloned upstream of *LUCm3* and co-transfected against the ARR22 and ARR2 variants: ARR2, ARR22, ARR2^D80N^ and ARR2^D80E^ (Fig 7). During these experiments, *WUSp*::*LUCm3* typically required a slightly longer incubation of 8 hours to provide an informative response range compared to the six hours required for the *ARR5p*::*LUCm3* promoter. In addition, the *ARR5p*::*LUCm3* promoter alone was also transfected alongside the *WUS* set to provide a known-target reference; relative values are referenced to the *WUSp*::*LUCm3* promoter alone.

As before, a weak induction of *ARR5p*::*LUCm3* by cytokinin was observed (Fig 7A and 7B), indicating that the experimental conditions are comparable to those previously described. The *WUSp*::*LUCm3* promoter occasionally showed very marginal, slightly higher expression when treated with cytokinin. It is known that *WUS* is only weakly induced by cytokinin [2, 13] and we could occasionally detect it here, about 1.25-fold (Fig 7B). ARR2-ox led to higher total response under mock and a boost in transcriptional activity upon the addition of cytokinin of *WUSp*::*LUCm3* (Fig 7A and 7B) resulting in a two-fold increase in cytokinin specific activity for *WUSp*::*LUCm3* (Fig 7C). Interestingly, the emission peak of ARR2-ox on *WUSp*::*LUCm3* was reproducibly delayed with respect to that of *ARR5p*::*LUCm3*, here 300 min compared to *ARR5p*::*LUCm3* at 175 min (Fig 7A and previous Figs). This suggests that the transcriptional complexes involving the two promoters are likely different. This was the first indication that careful inspection of the kinetic information may be exploitable in the future.

Moreover, ARR2-ox effects were nullified by the co-transfection of ARR22 implicating that a functional phosphorelay is responsible for ARR2 in activating *WUSp*::*LUCm3*. Likewise, overexpression of the constitutive active ARR2^D80E^ variant led to high *WUSp*::*LUCm3* reporter activity at the basal (S18 Fig) and total response levels independent of cytokinin (Fig 7B). The disabled ARR2^D80N^ variant was essentially inert under all conditions (Fig 7). As observed before, the ARR2 variants were stably expressed and showed no obvious differences during these experiments either as judged by immunoblot analysis (S19 Fig). In conclusion, we observe that the *WUSp*::*LUCm3* promoter responded similar to that of *ARR5*::*LUCm3* to the ARR2 variants (Fig 3) and ARR22 (Figs 2 and 3) co-overexpression with the exception of the peak emission timing. Taken together, this provides confirmatory evidence that *WUS* responds to cytokinin signaling in a TCS-dependent manner and that this response is, at least partially, classically mediated through B-type RRs such as ARR2, consistent with the recent reports of B-types affecting *WUS* expression [12–14].

### FACS-RT-qPCR corroboration analysis on the endogenous genes *ARR5*, *ARR7*, and *WUS*

A FACS-RT-qPCR experiment was conducted to corroborate that the cloned promoters *pARR5* and *pWUS* accurately captured the native regulatory output in our particular cell culture protoplasts. To do this, cells were transfected with *effector-GFP* fusions and GFP fluorescent cells were collected by Fluorescence activated cell sorting (FACS), which is a technique to isolate particular cells one at a time from a heterogeneous mixture based on their fluorescence profile. After collecting the GFP positive cells, RNA was reverse transcribed (RT) and quantified by quantitative-PCR (qPCR). qPCR primers were designed to monitor endogenous *ARR2*, *ARR1*, *ARR5*, *ARR7*, *WUS*, *ACTIN 2* (*ACT2*) and *ELONGATION FACTOR 2* (*EF2)*. As mentioned, *ARR5* and *ARR7* are known B-type targets [51]; *ARR1* and *ARR2* are reported to be unaffected by cytokinin treatment [48]. *EF2* served as the normalization reference control, with *ACT2* serving as the error reference control [69]. qPCR primers for *WUS* were designed to capture the 5’ or 3’ end of the gene. Additionally, where possible, two different C-terminal GFP fusions to wild-type ARR2 and ARR2^D80E^ were constructed (see Methods). Free-GFP was transfected to test the nominal response of the cells to cytokinin. Unlike the transfection experiments, the FACS experiments were incubated overnight (~16hrs) before experimental begin due to machine-time access. On each day, independent transfection samples for each construct were pooled and then split, one composing mock and the other *t*-zeatin treated samples. Cytokinin treated cells were incubated for 30 min before beginning each sort, which took on average 20 min comprising the peak expression level for most *A-type* genes [31]. The qPCR data are presented in Cq values, median normalized to reference *EF2*, and therefore stay in base2 (Ct, Cq changes).

**Fig 8:**
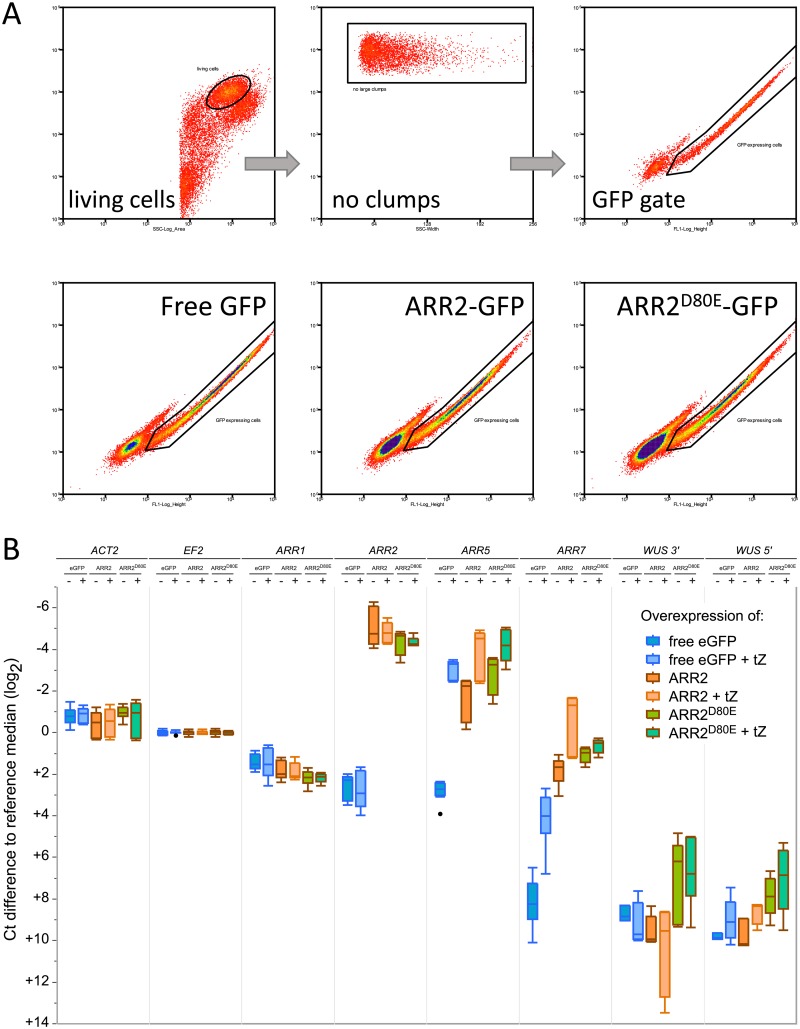
Endogenous gene regulation of *ARR5*, *ARR7* and *WUS* in response to ARR2 variants overexpression in cell culture protoplasts. (A) Overexpression of eGFP, ARR2-eGFP, ARR2^D80E^-eGFP were captured by FACS 16 hrs after transfection including cytokinin stimulus. (B) Ct(Cq) values of all samples were normalized to the median of *EF2* with differences shown in Ct(Cq) with respect to *EF2* (log_2_). Smaller values indicate a higher transcript rate. ‘+ tZ’ indicates 1 μM *t*-zeatin; target gene for qPCR is indicated as the principle column header. The data presented here are composed of three, complete experimental replicates. 4 μg was used for each effector per 60 μL at 3.5x10^6^ protoplasts mL^-1^; a total of eight 60 μL transfections was pooled and split for each experiment into mock and treated samples.

Although it was clear that the GFP fusions were expressed based on the FACS data, the localization of free-eGFP, ARR2-GFP and ARR2^D80E^-GFP was also examined by conventional epifluorescence microscopy. For all ARR2 variant fusions, the majority of the GFP signal was observed in the nucleus although some very weak GFP emission was also observed in the cytoplasm for ARR2^WT^ (S20 Fig). Inspection of Fig 8 confirmed that neither *ARR1*, *ARR2* nor *ACT2* was affected by cytokinin treatment. Endogenous *ARR5* and *ARR7* were induced roughly 5 to 6 fold by cytokinin very similar to that observed in studies with intact plants [26, 36, 47]. *ARR7* followed the same pattern as endogenous *ARR5* except that a more dramatic difference was observed for *ARR7* compared to *ARR5*, traceable to the overall much lower basal expression level of *ARR7* (+9 Ct relative to *EF2*) compared to *ARR5* (+3 Ct relative to *EF2*).

Overexpression of ARR2-GFP led to transactivation of both *ARR5* and *ARR7* without cytokinin, either matching or exceeding their cytokinin-inducible levels (Fig 8). This is different compared to the LUC assays without exogenous cytokinin, as ARR2-ox either matched or exceeded the mock levels (see Basal and Total Response Levels from Figs 2–7) and led to 7 to 30 times more specific activity the presence of exogenous cytokinin (compare Fig 8 with Figs 2–6). ARR2^D80E^-GFP-ox also drove *ARR5* and *ARR7* to levels either matching or exceeding their cytokinin-inducible levels (Fig 8) whereby ARR2^D80E^-GFP led to slightly higher levels than ARR2^WT^-GFP (Fig 8) consistent with its constitutive active confirmation (Fig 3, S6 Fig). Thus the high level of induction for endogenous *ARR5* and *ARR7* due to ARR2^WT^-GFP-ox appears to have left very little room for further induction by cytokinin, resulting in a modest boost of only 2 Ct more activity with *t*-zeatin (Fig 8). Similarly, ARR2^D80E^-ox led to a slightly higher expression than ARR2^WT^-GFP and a smaller (1 Ct) positive effect in the presence of cytokinin (Fig 8), similar to that observed during the LUC assays (Fig 3, S6 Fig). Thus, one can conclude that the cloned promoter region of *ARR5* behaved analogously to the native gene in the same system, with the caveat that without cytokinin, overexpression of wild-type ARR2 drove endogenous *ARR5* and *ARR7* to higher levels compared to the LUC assays, resulting in less cytokinin inducibility.

*WUS* on the other hand gave a slightly different answer. Endogenous *WUS* was actually not expressed in the GFP and ARR2-GFP overexpression samples, with or without cytokinin: the Ct was ≥ 36 Ct cycles, where the water control also started to give noisy results and many technical replicates produced no value at all (S1 Table). Thus, the normalized log expression level of ≥ +10(log_2_) was more indicative of “absence of transcript”. Consequently, endogenous *WUS* was not induced by ARR2 overexpression with or without cytokinin (Fig 8). With hindsight, this is no surprise as the *WUS* chromatin environment keeps the gene transcriptionally silent in cell culture [68] unless placed on Shoot-Induction-Medium [12–14, 70, 71]. ARR2^D80E^-GFP-ox was able to activate endogenous *WUS* by bringing the cycle numbers up from the non-noise range (≥ 36 Ct) to clearly present (to 33 Ct) (S1 Table) which corresponds to ~ +8(log_2_), an increase of 2(log_2_). As *WUS* was only transcribed in the presences of constitutive-active ARR2^D80E^ we conclude that it is highly likely that this constitutive-active version of ARR2 was able to eventually open the chromatin around the endogenous WUS enabling some transcription to take place.

Altogether, the FACS-RT-qPCR and the LUC reporter assay indicate that the cloned promoter regions and ARR2 overexpression reflect the protein networks that are natively present in the cell culture protoplasts. In comparison between the two approaches, the high dynamic response gained by the reporter assay suggests that it gives us a better ability to track the TCS phosphorelay activity compared to the endogenous readout. Overnight LUC assays lost their dynamic response when compared to shorter incubation times (S21 Fig), but performing a FACS-RT-qPCR 6 hours after transfection yielded data with identical results to the 16 hour incubations (S29 Fig). Differences between the endogenous *ARR5* and the reporter could be due to differences in the vector packing compared to the native chromatin, changes in the three-dimensional structure of the vector, unknown regulatory regions 3’ of the 5’ intergenic space. Alternatively, only a few copies of the endogenous *ARR5* promoter region are present per cell, whereas an unsaid number of *ARR5* promoter molecules are present in the transfection experiment, quite possibly diluting out the endogenous B-types over the number of promoter targets. The endogenous *ARR5* responded both to cytokinin treatment, and overexpression of ARR2 or ARR2^D80E^ corroborating the LUC assay generated data. That the endogenous *WUS* was weakly activated by ARR2^D80E^ suggests that the constitutive active form of ARR2 was eventually able to open the chromatin allowing transcription of the *WUS* gene. This might explain some of the strange, inexplicable phenotypic effects previously observed in transgenic plants expressing ARR2^D80E^ [37]. Likewise, ARR2 was able to activate the cloned reporter *WUSp*::*LUCm3* in an open chromatin context in a cytokinin dependent manner (Fig 7) demonstrating the utility and sensitivity of the LUC assay for identifying TCS targets.

## Conclusions

Multiple negative regulatory modes of the TCS have been discovered and proposed which fall into two major mechanisms that are not mutually exclusive. One is the mitigation and regulation of the amount of TCS~P within the system (phosphocompetition) and the other is regulation of the pathway(s) via direct and/or indirect interactions of TCS members with other components in the cell [3, 20]. The cytokinin-dependent phosphorelay works in the forward direction with respect to RR activation, and in particular with respect to B-types, gene activation. AHPs function as redundant positive regulators with the exception of AHP6 a natural H->N variant [3]; HKs AHK2, AHK3 and AHK4 positively, whereby AHK4 also functions bidirectionally, dependent on the absence or presence of cytokinin [72]. A-types are negative regulators of the TCS [20, 73]. In addition, negative feedback loops have been discovered as in the transcription induction of cytokinin degrading enzymes *CYTOKININ OXIDASE 4* and *CYTOKININ OXIDASE 5* via ARR1 [34]. Collectively, negative feedback regulation inhibits TCS information flow, which is the availability of TCS~P, presumably in the form of phosphorylated AHPs, but not exclusively.

Genome-wide analysis of ARR22-ox *in planta* revealed a drastic, blanket-wide attenuation of cytokinin response of genes from a myriad of protein classes [26]. ARR22 has so far not exhibited any auxiliary interaction phenotypes when its conserved phosphorylatable Asp is mutated and is thus thought to function strictly within the phosphorelay [11, 26, 45, 46]. In contrast, A-types function within and outside of the TCS phosphorelay via phosphorylation-determined interactions with other proteins [6, 20, 28]. The observations made here are consistent with this interpretation, as ARR22^D74E^ did not exert the dominant-negative effect of ARR22^WT^ on these B-types, leaving us to conclude that the phosphohistidine phosphatase activity of ARR22 is the most likely explanation. Small differences in the kinetics between ARR22, ARR22^D74N^, ARR22^D74E^, and ARR22^D74A^ can be observed from time to time (Fig 2A and 2C, S6 and S26 Figs, [49]). In particular, slightly more (and almost significant) specific activity was observed under cytokinin treatment (Fig 2, [49]) for ARR22^D74N^, ARR22^D74E^, and ARR22^D74A^ similar to what was observed for ARR7^D85N^ in transgenic plants [19]. In general however, these differences are minor compared to the dominant-negative effect that ARR22^WT^ exerted on the *ARR5p*::*LUCm3* reporter alone or when B-type proteins ARR2, ARR1, ARR10 or ARR18 were overabundant (see Figs 2–7). ARR22 therefore can be used to effectively block the phosphorelay output *in vivo* and provides a succinct way to determine gene targets in combination with B-type overexpression.

We can expand a proposed TCS phosphocompetition model [20] to gain a better interpretation of the B-type reporter output and possibly extrapolate that to the native systems *in planta* when we drive individual components to abnormally high levels via ectopic expression. The expanded model of TCS phosphocompetition can be broken up into four, non-exclusive components: 1) the TCS~phosphate sink/dilution model in which overexpression removes the available amount of AHP~P available to B-types as TCS~P is shunted to AHPs, A/C-types; 2) A protein lockup/sequester model in which AHPs, phosphorylated or not, are bound more frequently to the overexpressed A/C-types; 3) TCS~P signal decay due to auto-dephosphorylation in which TCS~P is irrevocably removed from the system; 4) squelching of phosphorylated A-types with phosphorylated B-types preventing interaction with other components. This last point was proposed by Lee et al., 2008 [19], but so far there is no evidence that this is possible; this option will not be considered.

A-types are negative regulators of the TCS [20, 73] and as a consequence, their rapid induction by cytokinin is predicted to result in creating a TCS~P sink and promoting signal decay by removal of TCS~P. Both A-types and B-types are thought to permit reverse phosphotransfer *in vivo* to and fro AHPs based on *in vitro* reaction kinetics [20, 27, 50]. A-types and B-types could very well contribute to TCS~P signal decay in plants, similar to that observed in other organisms where each RR has its own unique autodephosphorylation rate [56]. ARR22 has an autodephosphorylation rate [26] more rapid than A-types ARR3, ARR4 [6, 27] which are in turn more rapid than B-type ARR11 [50], whereby reverse phosphotransfer was never observed *in vitro* for ARR22 [26]. AHPs have a much, much slower autodephosphorylation rate compared to A-types or B-types *in vitro* [6, 27, 50] implying that loss of TCS~P is not likely to occur via AHPs and AHPs are not likely major drivers of TCS~P signal decay (Figs 4 and 5, [24, 33]).

If AHPs were limiting then additional AHP would enable faster phosphorelay to RRs. Unlike some experiments [62], AHP2-ox did not affect the expression of the *ARR5p*::*LUCm3* promoter alone (Fig 4) or *ARR6p*::*LUC* [33]. In contrast, both AHP5- and AHP2-ox lowered the basal and mock overexpression effects of ARR2 but could not affect cytokinin stimulation in ARR2-ox in any way (Figs 4 and 5). On the other hand, AHP5 led to a slight, typically non-significant, boost in reporter activity under the right conditions (compare Fig 4 with Fig 5). It could be argued that AHPs could work both positively and negatively with regards to phosphocompetition models. The interaction of AHPs with AHKs, A- and B-types is actually independent of an AHP’s phosphorylation state [74]. If we assume that the total TCS~P throughput is limited by AHK activity and that only a limited amount of an overexpressed AHP pool can be phosphorylated, then the protein lockup model predicts that AHP overexpression could sequester A-types, preventing an A-type from encountering and removing a phosphoryl group from a phosphorylated AHP. Likewise, the sink model proposes that an excess of AHP could simply sequester and dilute out the amount of AHP~P that B-types can encounter. Based on the slow autodephosphorylation rate of AHPs and that the phosphorelay to and from RRs is reversible and by conjecture very rapid, it would appear that AHPs do not pose a large hindrance to the phosphorelay when ARR2 was overabundant (Figs 4 and 5). By this accord, any positive or negative effects might be a result of favored or non-favored interactions within the TCS and not a result of phosphorelay kinetics.

Overexpression of A-types ARR7, ARR4, and ARR15 on the other hand, prevented cytokinin induction when expressed *alone*, consistent with A-types generating a TCS~P sink (Fig 5, S12 Fig). This was previously only observed in plants [19, 20]. In contrast, even though ARR4, ARR7, and ARR15 could partially repress the *basal* ARR2-ox effects, they were not able to outcompete cytokinin-dependent ARR2-ox effects (Fig 5B, S12 Fig), suggesting that what we observe is the potential reverse phosphotransfer to AHPs from the overexpressed A-types or release of AHP~Ps without phosphotransfer. A-type overexpression therefore is not effective enough to outcompete overexpressed B-types when the phosphorelay is strongly driven in the forward direction by exogenous cytokinin. There is evidence that the lockup/sequester model also makes sense for A-types. Overexpression of ARR7^D85N^ [19] did not exhibit a phenotype in plants and ARR5^D87A^-ox occasionally had a dominate negative effect [20], indicating that the unphosphorylated A-type proteins are not in an active conformation. Interestingly, ARR7^D85N^ resulted in a sustained activation of *ARR6* by cytokinin to which no effect was ascribed [19]. Our reporter system actually had output for ARR4^D95N^ and ARR4^D95E^ as that observed for transgenic ARR7^D85N^ plants ([19], S12 Fig) in that all lead to a slow, steady and sustained gene activation after the addition of cytokinin. It is theoretically possible that ARR4^D95N^ and ARR4^D95E^ are binding to phosphorylated AHPs but are not able to remove the phoshoryl group, sequestering AHP~P away from B-types and releasing AHP~P at a later time.

In summary, the cytokinin induction in our reporter assay followed a natural signal decay as observed with intact plant tissues [19, 31, 53]. Additionally, compared to other reporter assays, ours mimicked effects previously only observed in transgenic overexpression plants suggesting that we are looking at phosphocompetition in action. ARR22 is a functional RR of unknown function expressed at the chalaza/funiculus junction in *A*. *thaliana* that has dominant negative effects on TCS signaling when mis-expressed [45, 46]. This is unique to ARR22 as neither the overexpression of AHPs nor A-types have such a potent inhibitory effect when overexpressed (Figs 4 and 5, S12 Fig). All of the evidence points to ARR22 as working strictly within the phosphorelay as a phosphohistidine phosphatase (this work, [11, 26, 45, 46, 49]). It was proposed early on that its high phosphohistidine phosphatase activity could be leveraged in an inducible system to minder the phosphorelay [26, 46]. Such a study was recently conducted in 2013 using a dexamethasone inducible system [11] cleverly affirming mounting evidence that cytokinin negatively regulates cold and drought stress in *A*. *thaliana* [5, 10]. The potent inhibitory effect of ARR22 on the TCS also makes it an excellent tool for dissecting local phosphorelay-dependent effects when driven by tissue-specific inducible systems [11, 75, 76]. The combination of B-types with ARR22 and treatment specific stimuli should allow the identification of more direct target candidates of B-types, as shown here for *WUS*. It will be interesting to see if overexpression effects of ARR22 can also be transferred to other species or if ARR22 can only recognize plant specific HPts.

## Methods

### Transfection setup and LUC assay

Protoplasts and transfections were performed as previously described from an *A*. *thaliana* dark-grown cell culture cultivated at 23 ± 1°C [49]. Assays were transfected and treated simultaneously in a 96-well format using the 96-well manual pipette ‘Liquidator^96^’ (Steinbrenner) with the exception that the DNA was filled to 10 μL instead of 9 μL with ddH_2_O. Transfections and plate measurements were performed at 25 ± 1°C. Plasmid DNA was purified using Xtra-midi preparation kits (Macherey-Nagel) and kept at 4°C during storage. For every transfection, the total DNA load was kept absolutely the same to minimize competitive dilution effects. Six hours after transfection, the luciferase assay was started. 150 μL of each transfection was pipetted into two wells of a white ELISA plate. 15 μL of K3 with D-luciferin (K+ salt) was added to yield a final concentration of 200 μM using the Liquidator^96^ pipette. Luciferase activity was monitored *in vivo* with plate reader FluoroSkan Ascent FL (0.5 s integration, 5 min intervals for 600 min, PMT 812). Basal expression levels were measured for 60 min and then *t*-zeatin (in K3 Buffer) to a final concentration of 1 μM, or K3 Buffer alone, was added (also with the Liquidator^96^) to the treated or untreated samples, respectively. Linearity of the LUC emission was tested by transfecting *UBI4/2*::*LUCm3* and performing serial dilutions (S22 Fig).

The experiments were conducted 6 hours after transfection as this yielded the best signal/noise ratio. Waiting overnight resulted higher basal levels, but severely limited cytokinin responsiveness (S21 Fig) presumably due to accumulated protein that masked the inductive response. Saturation for the promoter alone was tested by varying the amount of reporter plasmid and the amount of D-luciferin, whereby here 3 μg *ARR5p*::*LUCm3* and 200 μM was saturating (S21 Fig). The reporter/effector relative concentration was tested to find a stable range of reporter activity. Transfection controls demonstrated that the method is extremely sensitive and amounts down to 125 ng of effector were detectable. In addition, we found that 0.5 to 2.0 μg ARR2 to 3 μg *ARR5p*::*LUCm3* showed stable and consistent treatment specific activity (S23 Fig). Either 1 μg or 2 μg effector DNA was used as indicated in each respective figure legend.

PEG-mediated transfection assays are known to carry the highest variance between batches [77]. To account for this, all comparative components were restricted to the same batch of cells and transfected at one sitting and evaluated from a single 96-well plate in a maximum of 12 blocks for reporter and effector combinations, including plus/minus cytokinin. In addition, at least two biological replicates were performed and from each biological replicate two technical replicates were made, yielding a minimum of four measurements (examples in S24 Fig). Protoplasts were randomly distributed to wells to ensure the random sampling hypothesis before the actual transfection event. Stable transfection efficacies across a plate were confirmed by cytometry (S25 Fig).

It was observed that the total relative output was reduced when the reporter plasmid pool was kept constant when challenged with increasing amounts of another plasmid (S23 Fig). We theorize, but did not exhaustively investigate here, that all plasmid/DNA species are uptaken as one pool (the DNA pool) and not as independent events, as such it is expected that the more other species is added then it will reduce the *relative* amount the others are uptaken. When effector combinations were used, the total DNA load was calculated and leveled with the empty effector vector in those samples with fewer combinations. Therefore, the total DNA amount (in μg) was always maintained to ensure an equal uptake probability. Therefore, 3 μg of *ARR5*::*LUCm3* was used with 2 μg or 1 μg for each effector plasmid and the empty effector cloning plasmid *pHBTL-3xHA* [78] was substituted for lacking effector plasmids ensuring an equal, total DNA load. For example for Fig 2, we have 3 μg of *ARR5*::*LUCm3* + 2 μg *ARR2* + 2 μg *ARR22* or 3 μg of *ARR5*::*LUCm3* + 2 μg *ARR2* + 2 μg *empty pHBTL-3xHA* plasmid or 3 μg of *ARR5*::*LUCm3* + 4 μg *empty pHBTL-3xHA* plasmid and so on. Overall, the transfection variance could be adequately measured and representative experiments are shown. Replicates that clearly failed based on visual inspection were excluded from the analyses (e.g. D-luciferin not added). We also explored other normalization techniques throughout this paper. Where overall transfection levels still varied, but the biological readout was similar but not the same, the data were normalized using the method of quantile normalization (QN) (Fig 2, see Math, S26 Fig). Where doubt was still present in transfection variance, *pCF203* encoding *eGFP* was co-transfected and the GFP intensity for the population was obtained by flow cytometry or in a microtiter fluorescence plate reader allowing the LUC emission levels to be rescaled according to the GFP emission level (e.g. S27 Fig). Where needed, these techniques were combined.

### Determination of GFP expression intensity in a population

Cells expressing GFP were identified by excitation with a 488 nm laser and comparing peak (FL1) to shoulder (FL2) emission with either an MoFlo XDP (BC; FL1 = 534/30, FL2 = 585/29) or CytoFLEX (BC; FL1 = 525/40, FL2 = 585/42) cytometer; percentage and mean intensities were calculated using the respective software for each machine, i.e. Summit 5.4 for XDP and CytExpert for CytoFLEX. The gating strategy was the same as described in Fig 8. Flow rates were kept to the absolute minimum. Alternatively, GFP expression was monitored in a TECAN Safire fluorescence plate readers using 488 nm ± 5 nm excitation and 515 ±12 nm emission with all other parameters at default levels; Z-gain and detector gain was taken from the brightest well. A comparison between the two methods is provided in (S28 Fig); a linear relationship to the number of cells and total mean emission was obtained for both methods.

### Math

Replicates were checked visually to ensure reproducibility and confirm proper experimental execution. Thereafter, experiments were discarded and repeated if the biological and technical replicates were significantly different before treatment. Experiments determined biologically reliable were evaluated and each experimental data set was performed 3 to 5 times or as needed in order to validate the observations; the most representative dataset was chosen for presentation or quantile normalized. The data were analyzed in JMP (SAS) after labeling with a homemade program (LUC_Organizer.jar, KWB; available upon request).

Significance tests were always performed with the non-normalized data using Fischer’s Least Significance Test (LSD) and an alpha level of 0.01 as indicated directly in the graph; significance classes not sharing a letter are significantly different at that level. Note, the LSD test uses the pooled variance for class comparisons. Plots were printed as PDFs from JMP graph output and organized (but not modified) in Adobe Illustrator. Box plots show the median (horizontal line) with the box ends representing the 75th and 25th quantiles; whiskers extend from the box ends to the outermost data point of either quantile side as 1.5*(interquartile range). Data points outside these ranges are outliers.

Specific Activity was calculated by ranking LUC activates and taking the rank-difference of treated to un-treated. Large variance in transfection rates between plates could be normalized by quantile normalization (QN) in R (http://www.r-project.org/) using the normalize.quantiles function of the preprocessCore Package. The raw and QN values used for Fig 1 are shown in S26 Fig. The Area under the Curve (AUC) was calculated as shown in Fig 1 directly in JMP (using a JMP formula) followed by exporting the cumulative value to a separate table for analysis. Alternatively, LUC activity/GFP-mean intensity was explored in Fig 3, S27 and S28 Figs as described elsewhere in the Methods and figure legends.

### Cloning

The full-length promoter, defined as from ATG of the respective coding region until the next upstream genic region. *ARR5p* (-2252 to +6) is previously described [49]. *WUSp* (-1741 to +1) was cloned into *pBT8-LUCm*^*3*^ via EcoRI and NcoI restriction sites; both are marked in S1 Text. Effector cDNAs for *ARR1*, *ARR2*, *ARR10*, *ARR18*, *AHP2*, *AHP5*, *ARR7*, *ARR4*^*DXX*^, *ARR15* and *ARR22*^*DXX*^ were obtained or cloned in *pENTR* (see S2 Table and Acknowledgements). cDNAs for *ARR4*^*DXX*^ were previously described [6]. Site-directed mutagenesis of *ARR2* cDNA had been used to generate ARR2^D80N^, ARR2^D80E^. cDNAs were cloned into *pHBTL-3xHA-GW* [78] by Gateway^™^ recombination. ARR2^DXX^ cDNAs were recombined into *pABind-GFP* [79] and *pUBQ10(pUGT1-GW)* for C-terminal GFP fusions.

### Immunoblotting

To confirm the protein expression of HA tagged proteins, cells were pooled and pelleted at 1000g for 5 min at RT after adding five to ten volumes of W5 [154 mM NaCl, 125 mM CaCl_2_ x 2 H_2_O, 5 mM KCl, 5 mM glucose, pH 5.9]. Glass beads (∼0.2 mm in diameter) were added, followed by 80 to 200 μl of 1x SDS-Laemmli buffer. The cells were vortexed for 2 min and then incubated at 65 °C for 10 min or 95°C for 1.5 min. Debris was removed by centrifugation at 13,000 rpm for 5 min and the supernatant transferred to a new Eppendorf tube. Proteins were then separated using standard SDS-PAGE on a 12% poly:bis-acrylamide (37.5:1) gel and transferred to a PVDF membrane. Membranes were blocked with 5% milk powder in TBS-T followed by incubation with monoclonal antibodies 1:1000 α-HA (rat 3F10; Roche) and 1:5000 α-Rat-AP (Sigma A-9654) or α-HA (mouse ab-24779; abcam) and α-mouse-AP (Bio-Rad Cat.170-6520). AP activity was monitored with NBT+BCIP. POD was detected with anti-HA-POD (1:1000, rat, Roche) and chemiluminescence was imaged with a PEQLAB Fusion Fx7 Imaging System.

### FACS-RT-qPCR

#### FACS

*ARR2-eGFP* and *ARR2*^*D80E*^*-eGFP* from two different vector clones (*pUGT1*, *pABindGFP*) were transfected separately and later pooled before FACS on a MoFLo XDP (Beckman Coulter). *eGFP* was expressed from plasmid *pCF203* [80]. GFP positive cells were identified by gating on cells that had a positive shift in the FL1 (534/30) vs. FL2 (585/29) split with a 560DLP to compare intrinsic autofluorescence from round-living cells identifiable by gating on FSC-LogArea vs SSC-LogArea. Thereafter the “living-round” cells region was used as a primary gate (see Fig 8). The MoFlo XDP was mounted with a 100 μm nozzle and standard PBS buffer as sheath. 3 x 10^4^ GFP positive cells were isolated by FACS (sample/sheath pressure around 30.5/30.0 psi) in Enrichment Mode (1 drop) sorted directly into 350 μL RLT-Lysis Buffer (Qiagen) containing 5 μL 14M β-Me, constantly kept on wet-ice (+4°C). Sorting took on average 20 minutes. Cells were either mock treated (water) or treated with *t*-zeatin to a final concentration of 1 μM *t*-zeatin; cytokinin treated cells were therefore collected from time points 30 min to 50 min after stimulation. Cells were vigorously mixed after sorting, spun down 1000g for 30 sec and kept on ice before RNA extraction. Primers are in S2 Table.

#### RT-qPCR

RNA was isolated using the Qiagen RNeasy Plant Mini Kit according to manufacturer’s instructions after FACS with the following changes: Directly starting with step 3, repeat step 5 by reapplying flow-through, instead of step 6, perform an on-column DNase digest. Finally RNA was eluted with 23 μL RNase-free water into 1.5 mL tube. cDNA was synthesized using the ThermoFisher RevertAid First Strand cDNA Synthesis Kit following the manufacturer’s instructions and stored at -20°C if necessary. For real-time qPCR, 4 μL of diluted cDNA (1:3) was used with 5 μL BIO RAD iScript^™^ Reverse Transcription SYBR green Supermi with 0.5 μL of each primer (10 μm) in a final volume of 10 μL. SYBR was detected every 3 sec with a Bio-Rad CFX96 TouchTM Real-Time PCR Detection System; Cq was determined as single threshold for each trace. The Cq values were then normalized to median expression of *EF2* (AT2G45030) and Cq differences (*Sample*–*EF2*, log_2_) were calculated for each technical replicate to the median *EF2* expression, thereby preserving this variance. Biological variance was obtained my making multiple runs of the same experimental combination.

## Supporting information

S1 Fig Immunoblot analysis of ARR2 with ARR22 variants. (PDF)

S2 Fig Basal activity AUC values for experiment Fig 2. (PDF)

S3 Fig Structural alignment of ARR2 REC domain with PhoB and CheY. A structural alignment was performed using PROMALS3D (PROfile Multiple Alignment with predicted Local Structures and 3D constraints) http://prodata.swmed.edu/promals3d/promals3d.php. ARR2 protein sequence was obtained from TAIR (www.arabidopsis.org) and aligned to the *E*. *coli* structures of PhoB (PDB: 1B00, https://www.rcsb.org/structure/1b00) and CheY (PDB: 2CHF, https://www.rcsb.org/structure/2chf). The conserved acidic pocket residues of the REC domain require a divalent cation (e.g. Mg^2+^, shown as a pink circle), are required to enable the phosphotransfer reaction (Bourret, 2010). The conserved Asp that is nominally phosphorylated is indicated with a ~P blue circle (Bourret, 2010). The structural alignment shows that the ARR2 REC domain is predicted to have the same structure as that of CheY, that is following the same five (αβ)_5_ structure hallmark of response regulators (Bourret, 2010).
Bourret RB. Receiver domain structure and function in response regulator proteins. Curr Opin Microbiol. 2010;13(2):142–9. Epub 2010/03/10.
(PDF)

S4 Fig Basal activity AUC values for experiment Fig 3. (PDF)

S5 Fig Overexpression subset experiment. Example raw fold changes seen with ARR2 variants ARR2^D80N^ and ARR2^D80E^ overexpression from a subset experiment; circumvention of ARR2^D80E^ over ARR22; comparison of a Parsley *ubiquitin* promoter *UBI2/4* to *ARR5p* under the influence of ARR2^D80E^. (A) Rough schematic of the core domains considered is this work. The point mutations introduced into the phosphoactive Asp in ARR2, Asp80, are shown as arrows. The GARP domain is the DNA binding domain (Hosoda et al., 2002). The rest of the protein has a large, uncharacterized output domain. (B) *in vivo* light emission curves obtained over 11 hours. Data is shown for one experiment, containing 4 replicates per sample type. (C) The mean relative expression level from the first hour after adding D-luciferin and before treatment with cytokinin. Ideally the samples dedicated for mock and for treatment at this point should have no major differences. (D) The total area under the curve was calculated after excluding the first hour, that is, beginning after treatment. (E) After ranking (see Methods), the AUC_cyt_−AUC_mock_ was calculated for all four sets, giving us the area corresponding to the space above the mock treatment and bordered by the cytokinin treated emission lines. (F) *in vivo* light emission curves obtained over 11 hours. Data is shown for one independent experiment, each containing 4 replicates per sample type. (G) *in vivo* light emission curves obtained over 11 hours. Data is shown for one independent experiment, each containing 4 replicates per sample type.
Hosoda, K., et al. (2002). Molecular structure of the GARP family of plant Myb-related DNA binding motifs of the Arabidopsis response regulators. The Plant cell 14:2015–2029.
(PDF)

S6 Fig ARR2^D80E^ overexpression effects on *ARR5p*::*LUCm3* is not affected by ARR22 overexpression, whether ARR22 has its phosphoactive Asp74 or not. Light emission curves from a separate transfection compared to Fig 3 are shown for ARR2^D80E^ along with ARR22^WT^, ARR22^D74A^ or ARR22^D74E^. Although some variance was seen, overexpression of ARR22 in any form did not block the constitutive-like effect of ARR2^D80E^.
(PDF)

S7 Fig Immunoblot analysis of ARR22 variants and ARR2^D80E^ for Fig 3 and S6 Fig. (PDF)

S8 Fig Immunoblot analysis of ARR2, ARR2^D80N^, ARR2^D80E^ used in experiment Fig 4. (PDF)

S9 Fig Basal activity AUC values for experiment Fig 4. (PDF)

S10 Fig Immunoblot analysis of AHP2 and AHP5. (PDF)

S11 Fig Basal activity AUC values for experiment Fig 5. (PDF)

S12 Fig Analysis of other A-types in comparison with ARR22 on ARR2 overexpression function. Light emission curves are shown from two independent experiments comparing three A-types ARR4, ARR7, and ARR15, with ARR2. The active TCS-Asp of ARR4 was mutated to Asn (ARR4^D95N^) or Glu (ARR4^D95E^). As explained in the main body text, all A-types were able to block the cytokinin induction when singularly expressed, but were not able to block ARR2 overexpression effects as observed with ARR22.
(PDF)

S13 Fig Immunoblot analysis of A-types ARR4, ARR4^D95E^, ARR4^D95N^, ARR7 and ARR15 used for experiment Fig 6. (PDF)

S14 Fig Published interactions AHP and ARRs using in this study. Published protein-protein interactions between the proteins used in this study were obtained from the bioBIND database and two graphs constructed with Cystoscope show the interaction method (A) and their corresponding studies (B). References with their Pubmed ID (PMID) are given in the Figure.
(PDF)

S15 Fig Basal activity AUC values for experiment Fig 6. (PDF)

S16 Fig Immunoblot analysis of various B-types ARR1, ARR2, ARR10, ARR18 used in this study. (PDF)

S17 Fig B-type overexpression effects on *ARR5p*::*LUCm3* are not affected by ARR22^D74E^-ox. Light emission curves are shown from co-transfection of the promoter-reporter along with ARR1, ARR2, ARR10 or ARR18 and ARR22^D74E^. Although some variance was seen, overexpression of ARR22^D74E^ in any form did not block the constitutive-like effect of B-type overexpression.
(PDF)

S18 Fig Basal activity AUC values for experiment Fig 7. (PDF)

S19 Fig Immunoblot analysis of ARR2 and variants used during the *WUS* experiments shown in Fig 7. Immunoblot analysis of ARR2 variants co-transfectred along with the *WUS* promoter. This immunoblot was run with other samples which are not relevant for the current manuscript and were removed from the image; all samples are from the same blot and experimental run.
(PDF)

S20 Fig Example epifluorescence analysis of GFP emission for free GFP, ARR2-eGFP and ARR2^D80E^-eGFP in transfected protoplasts used for experiments in Fig 8. (PDF)

S21 Fig Reporter assay limits for cloned promoter *ARR5p* comparing 6 hr with overnight incubation times. (A) Comparison of total light emission values with respect to the amount of reporter DNA. (B) Comparison of two different D-luciferin concentrations (C) Comparison of two different cytokinin concentrations (nM) and amount of reporter DNA. The assay conditions show that 3 μg reporter is saturating and that the substrate D-luciferin is not limiting. In addition, although overnight incubations led to higher levels of emission presumably due to accumulation of LUC protein the induction dynamism was lost. Shorter incubation times therefore resulted in better signal-to-noise ratios with respect to cytokinin treatments.
(PDF)

S22 Fig Testing LUC assay and cell load limits using *pUBI4/4*::*LUC*m^*3*^ and the linearity response of the assay. Cells were transfected with doubling amounts of plasmid DNA and assayed as described in the methods. Afterwards different amounts of cells from those transfections were distributed to microtiter wells and the *in vivo* emission was measured for one hour; two bio-replicates are shown. The sum of the emission is shown. The emission response doubled both in response to the number cells provided to the wells and also to the amount of DNA. This demonstrated that under these conditions and using a constitutive promoter we get a linear response in the number of cells emitting light due to the LUC reaction and is also linearly proportional to the DNA load.
(PDF)

S23 Fig Controlling transfection variance by controlling DNA load. One aspect that is a concern for all protoplasts transfection experiments is the issue of unequal DNA loading. We are able to show that inter-plate variance is uniform (see S25 Fig). However, we were still concerned that DNA loading effects, which are reported occasionally in the literature, are a concern. Therefore two experiments were set up: (A) a test of the dynamic range of the reporter readout as a function of varying amounts of effector yet still under constant DNA load (here ARR2); and (B) the overall effect of adding more and more plasmid, as many of these experiments demand several DNA species. (A) In the experiments the reporter plasmids were set at a fixed 3 μg, the effectors at 2 μg. Here the total “effector” DNA load was kept at 2 μg by using the same backbone vector (“empty = *pBHTL-3xHA*”) used for all of the effectors (see Methods) and as in all other experiments the reporter was 3 μg, yet the effector ARR2 was varied from 2 μg down to 125ng of DNA. In principle similar response curves were obtained when *3xHA-ARR2* was co-transfected at 0.5 to 2 μg, see in the emission curves, the total AUC and also in the specific activity. Below this level (250 ng, 125 ng) a loss in the dynamic response was observed. In conclusion, the data support the hypothesis that the overexpression of ARR2 is likely maxed out and that differences we observed were not due to insufficient loading. (B) Here, the effect of adding more and more DNA to a fixed amount of effector (ARR2, 2 μg) and reporter (3 μg) was explored. Basically, the tolerance was only 1 μg extra DNA, after which the total response range went down. Therefore, in any experiment where multiple plasmids were used, the largest combination set the total DNA load and this load was met with the empty effector backbone vector. All of the effectors were cloned into the same vector, *pBHTL-3xHA*, therefore differences in three-dimensional typology of the vector should not play a role in our experiments here.
(PDF)

S24 Fig Example pipetting schemes for four or six sample measurements. Each biological replicate is split into two technical replicates to catch subsampling errors. The biological replicates captures variance between transfections. Grey dots: where transfections occur in 96-deep well plates. Arrows show splitting into LUC microtiter plate. Green dot shows the position of transfection control (in this case using GFP and flow cytometry, see S25 Fig). Promoter-effector, bio-technical replicates, and mock or cytokinin treatments are shown as color blocks. 30 μL transfections make one mock and one treatment sample and 60 μL transfections yield two mock and two treatment samples.
(PDF)

S25 Fig Pipetting schemes for monitoring the transfection efficiency across a plate. Since the biological replicates are split, the remaining space on the plates could be used to monitor the transfection efficiency across the plate (green dots). An example sampling is shown here including the flow cytometric output. Here were see that on this particular day the GFP reference gave about 8% at any position across the plate. We conclude that the entire plate had an even transfection as expected. Of course the total transfection rates can vary depending on batch variation (see S26 Fig for an example); but we can be sure that per plate the total transfection rate will be similar using this method.
(PDF)

S26 Fig Datasets used for Fig 1. The raw data light emission curves (top) are followed by the quantile normalized datasets (middle). Notice that that the inter-plate variance was removed (bottom) without destroying the intra-plate variance (compare before and after quantile normalization). In this way, the total experimental variance was captured instead of using the “representative experiment” approach. However, we see that even when the total transfection levels varied, the representative approach would yield very similar results. Therefore, representative experiments were individually evaluated and are included in the S1 Dataset; all other data sets are available on request.
(PDF)

S27 Fig Comparison of the light emission curves with and without GFP emission as a scaling factor are overlaid for data given in Fig 3. Variance is shown as boxplots and the mean overlaid.
(PDF)

S28 Fig Comparison of measuring GFP emission levels with a microplate reader and a cytometer. (A) GFP emission obtained from empty cells, cells transfected with water only and cells transfected with varying amounts of plasmid encoding *GFP* (*pCF203*). Only cells transfected with GFP have emission above that of the plate autofluorescence indicating that the cellular autofluorescence does not contribute the emission signal. (B) Comparison of the GFP emission from those cells transfected with GFP in (A) between the microplate reader and a cytometer graphed against the number of cells expressing GFP quantified using the cytometer. A direct and linear correlation with the number of transfected with the emission intensity is obtained by both methods. C) Cells were transfected with 2μg *pCF203* and the diluted in K3 and distributed to various wells in a microtiter plate. We obtained a decent linear relationship with the number of cells and the GFP emission with a minimal of 75 μL of cells needed to have a signal securely above the plate autofluorescence. This indicates that under these conditions one can use this set up to monitor the transfection rate variance and the cell distribution variance by monitoring *in vivo* GFP emission. See Methods to obtain the settings for the microplate reader and the cytometer.
(PDF)

S29 Fig Analysis of *ARR5* and *ARR7* by FACS-RT-qPCR 6 hours after transfection. A FACS-RT-qPCR experiment was conducted 6 hours after transfection following the same procedure as for Fig 8 for genes *ARR1*, *ARR2*, *ARR5*, *ARR7* and *EF2*. As in Fig 8, effector plasmids encoding ARR2-eGFP, ARR2^D80E^-eGFP or free-eGFP were transfected into protoplasts, either mock (-) or treated with 1 μM *t*-zeatin (+). Each effector treated sample was normalized to the median of *EF2* with differences shown in Ct(Cq) with respect to *EF2* (log_2_). Smaller values indicate a higher transcript rate.
(PDF)

S1 Table Positive qPCR calls in Fig 8 is the number of replicates that had a measurable Ct value. A total of three biological replicates with each three technical replicates yield nine replicates. Observe that the positive qPCR calls for *WUS* was on average 4/9 with free eGFP or overexpression of ARR2, but was 8/9 when ARR2^D80E^ was overexpressed.
(PDF)

S2 Table Full list of primers used in this study. (XLSX)

S1 Text Sequences, TCS cis-element analysis, and cloning strategies of the promoter regions used in this study for *ARR5* and *WUS*. (PDF)

S1 Dataset Data underlying findings. (ZIP)
