# Supplementary figures and images for: ARR22 overexpression can suppress plant Two-Component Regulatory Systems
Source: PLoS One. 2019 Feb 11;14(2):e0212056. doi: 10.1371/journal.pone.0212056 (PMC6370222; doi:10.1371/journal.pone.0212056)

# Basal Expression Level

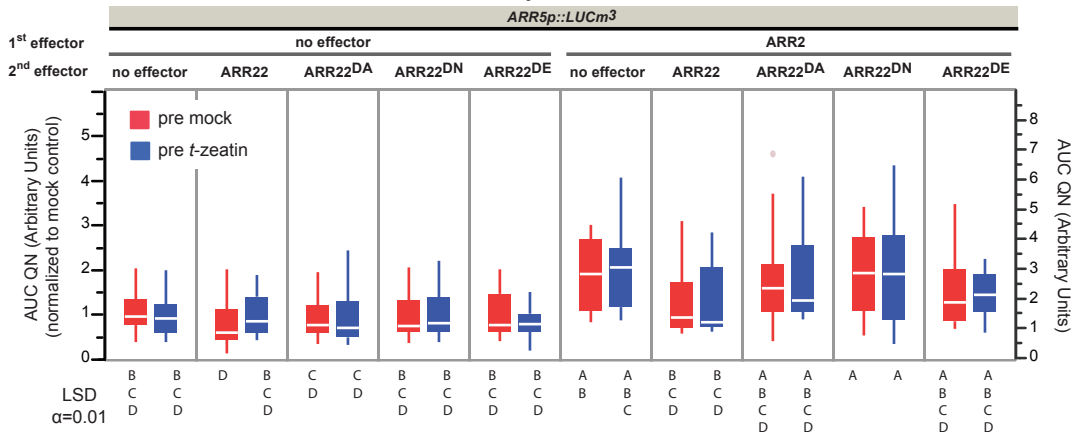

S2 Fig

Supplement: S2 Fig — (PDF) [file pone.0212056.s002.pdf]

# Basal Expression Level

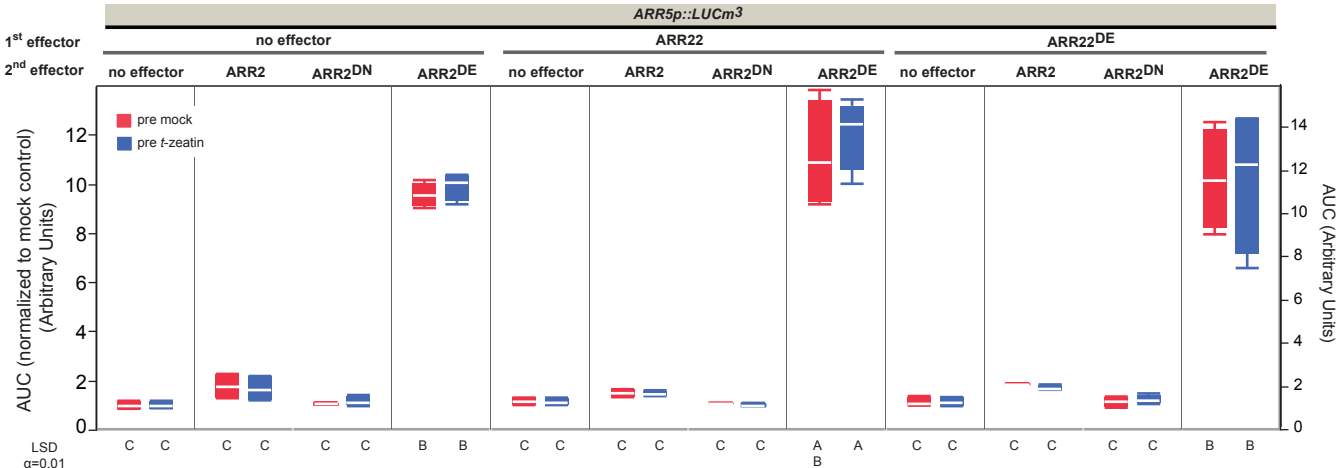

S4 Fig

Supplement: S4 Fig — (PDF) [file pone.0212056.s004.pdf]

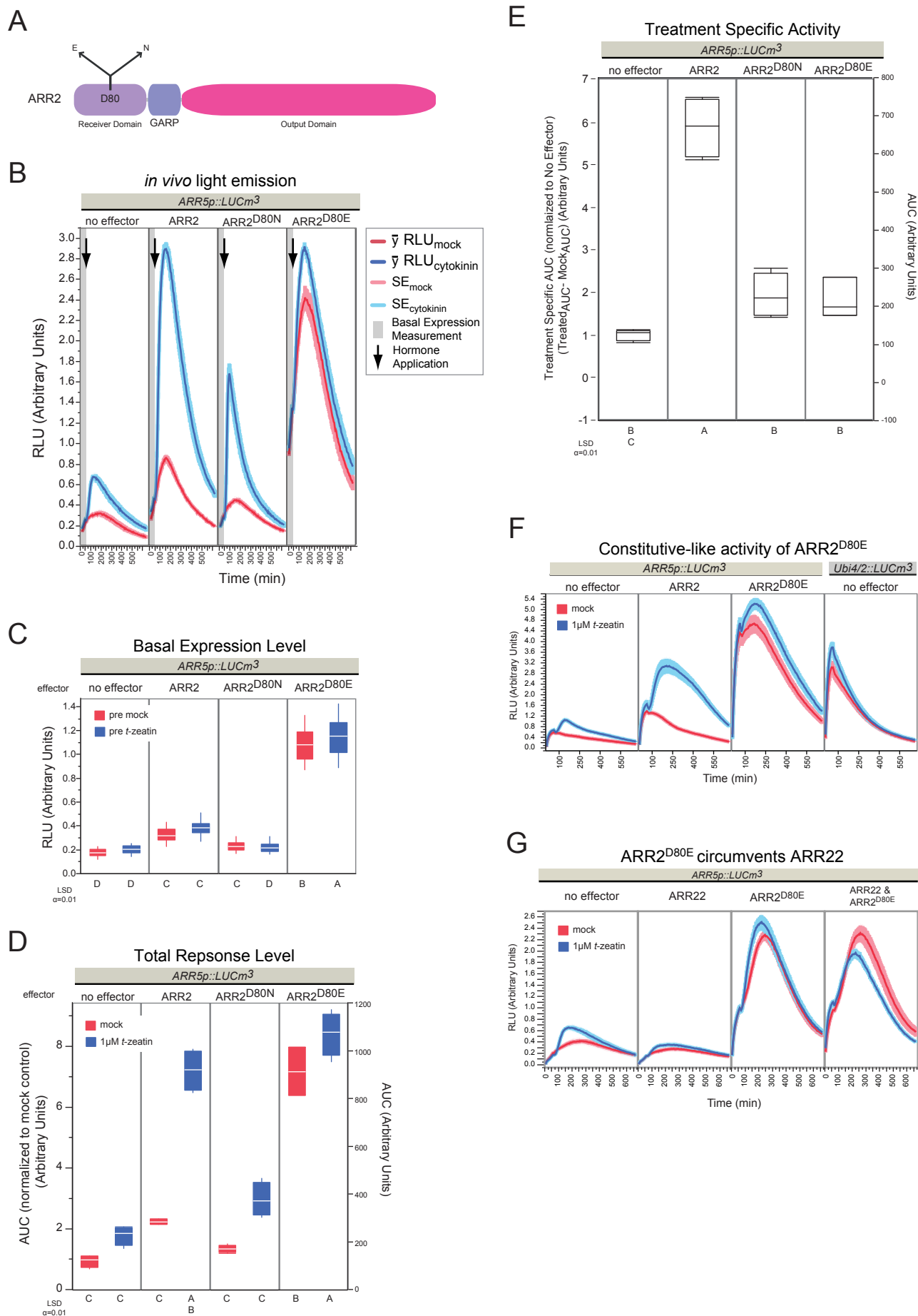

S5 Fig

Supplement: S5 Fig — Example raw fold changes seen with ARR2 variants ARR2D80N and ARR2D80E overexpression from a subset experiment; circumvention of ARR2D80E over ARR22; comparison of a Parsley ubiquitin promoter UBI2/4 to ARR5p under the influence of ARR2D80E. (A) Rough schematic of the core domains considered is this work. The point mutations introduced into the phosphoactive Asp in ARR2, Asp80, are shown as arrows. The GARP domain is the DNA binding domain (Hosoda et al., 2002). The rest of the protein has a large, uncharacterized output domain. (B) in vivo light emission curves obtained over 11 hours. Data is shown for one experiment, containing 4 replicates per sample type. (C) The mean relative expression level from the first hour after adding D-luciferin and before treatment with cytokinin. Ideally the samples dedicated for mock and for treatment at this point should have no major differences. (D) The total area under the curve was calculated after excluding the first hour, that is, beginning after treatment. (E) After ranking (see Methods), the AUCcyt−AUCmock was calculated for all four sets, giving us the area corresponding to the space above the mock treatment and bordered by the cytokinin treated emission lines. (F) in vivo light emission curves obtained over 11 hours. Data is shown for one independent experiment, each containing 4 replicates per sample type. (G) in vivo light emission curves obtained over 11 hours. Data is shown for one independent experiment, each containing 4 replicates per sample type. Hosoda, K., et al. (2002). Molecular structure of the GARP family of plant Myb-related DNA binding motifs of the Arabidopsis response regulators. The Plant cell 14:2015–2029. (PDF) [file pone.0212056.s005.pdf]

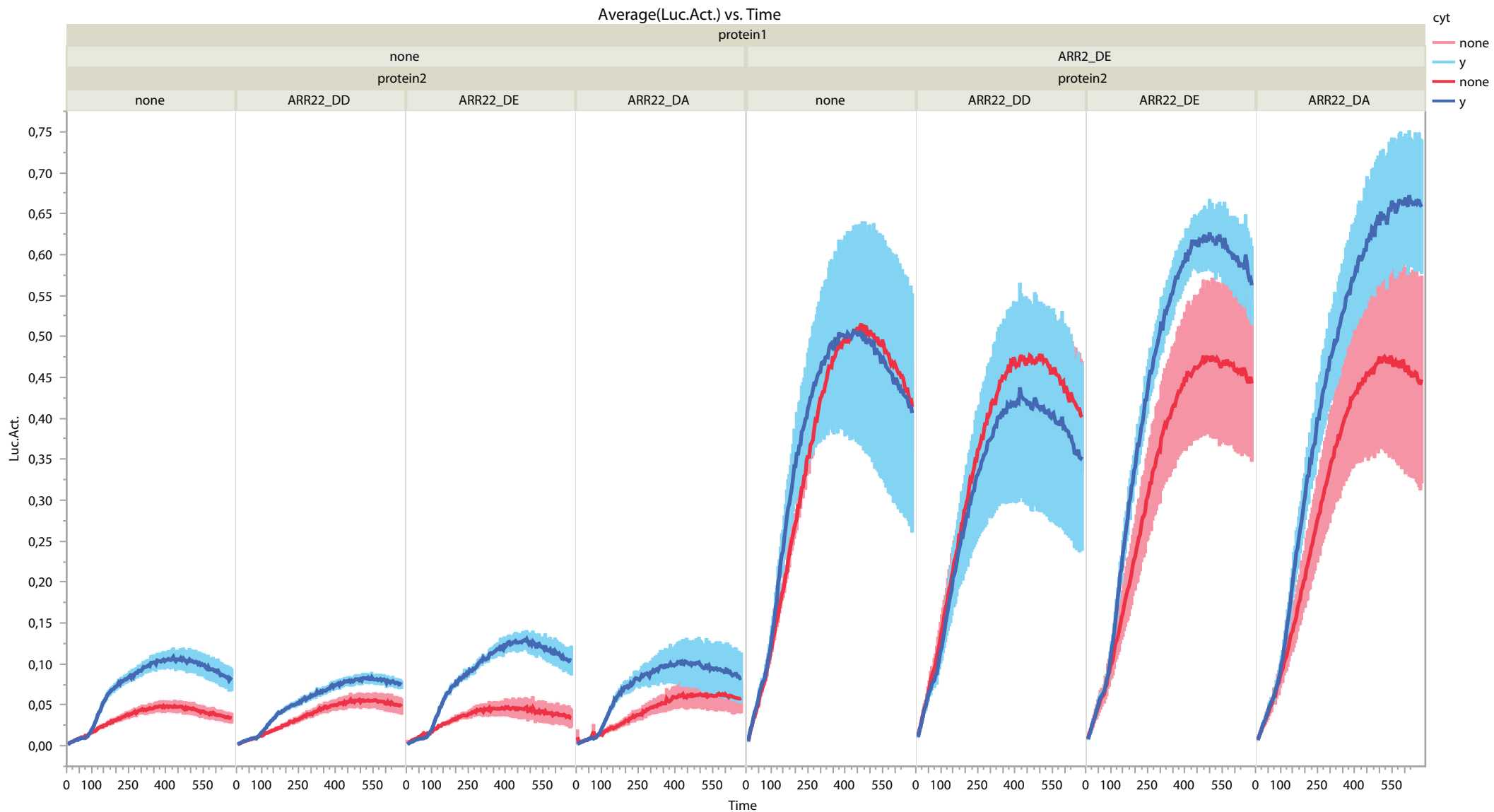

S6 Fig

Supplement: S6 Fig — Light emission curves from a separate transfection compared to Fig 3 are shown for ARR2D80E along with ARR22WT, ARR22D74A or ARR22D74E. Although some variance was seen, overexpression of ARR22 in any form did not block the constitutive-like effect of ARR2D80E. (PDF) [file pone.0212056.s006.pdf]

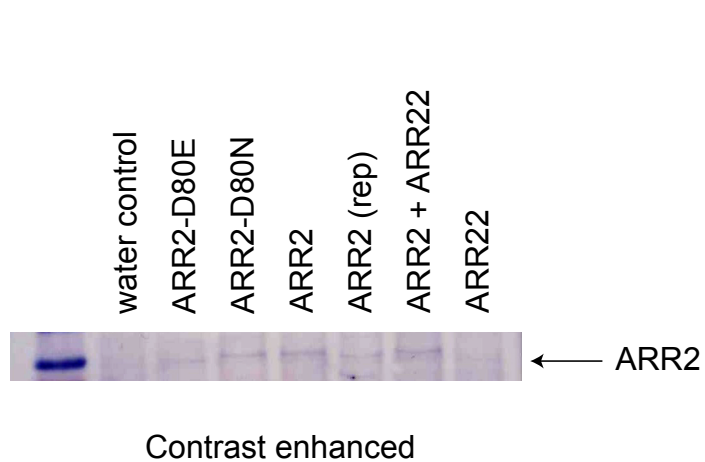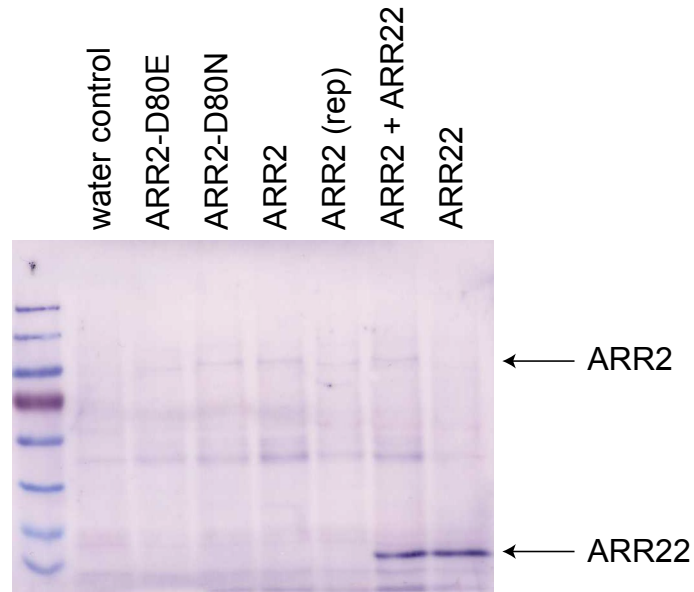

Specific Detection with  $\alpha$ -HA-rat/ $\alpha$ -rat-AP

S8 Fig

Supplement: S8 Fig — (PDF) [file pone.0212056.s008.pdf]

# Basal Expression Level

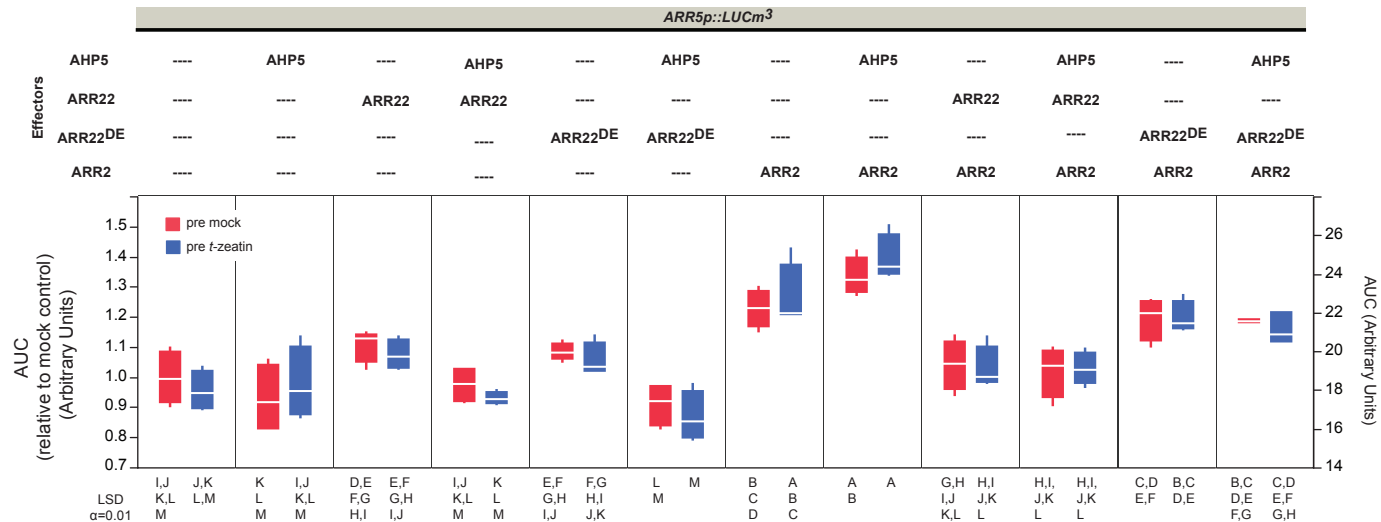

S9 Fig

Supplement: S9 Fig — (PDF) [file pone.0212056.s009.pdf]

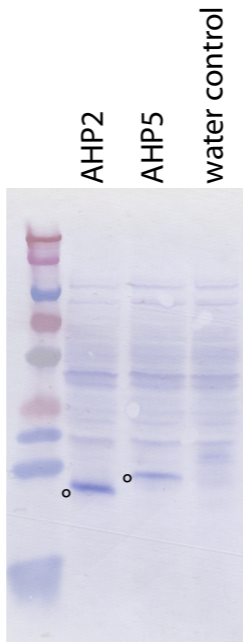

Primary antibody  $\alpha$ -HA (ab-24779), followed by  $\alpha$ -mouse-AP.  
° indicates the specific protein band.

Supplement: S10 Fig — (PDF) [file pone.0212056.s010.pdf]

# Basal Expression Level

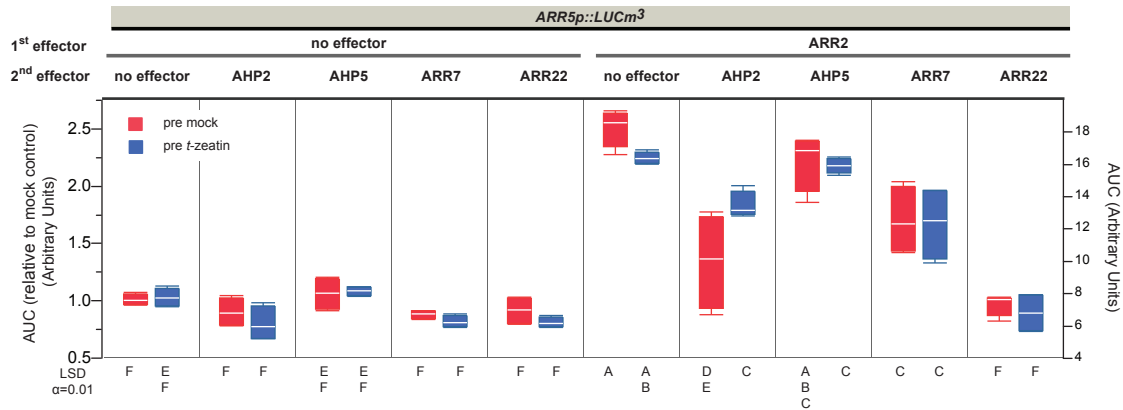

S11 Fig

Supplement: S11 Fig — (PDF) [file pone.0212056.s011.pdf]

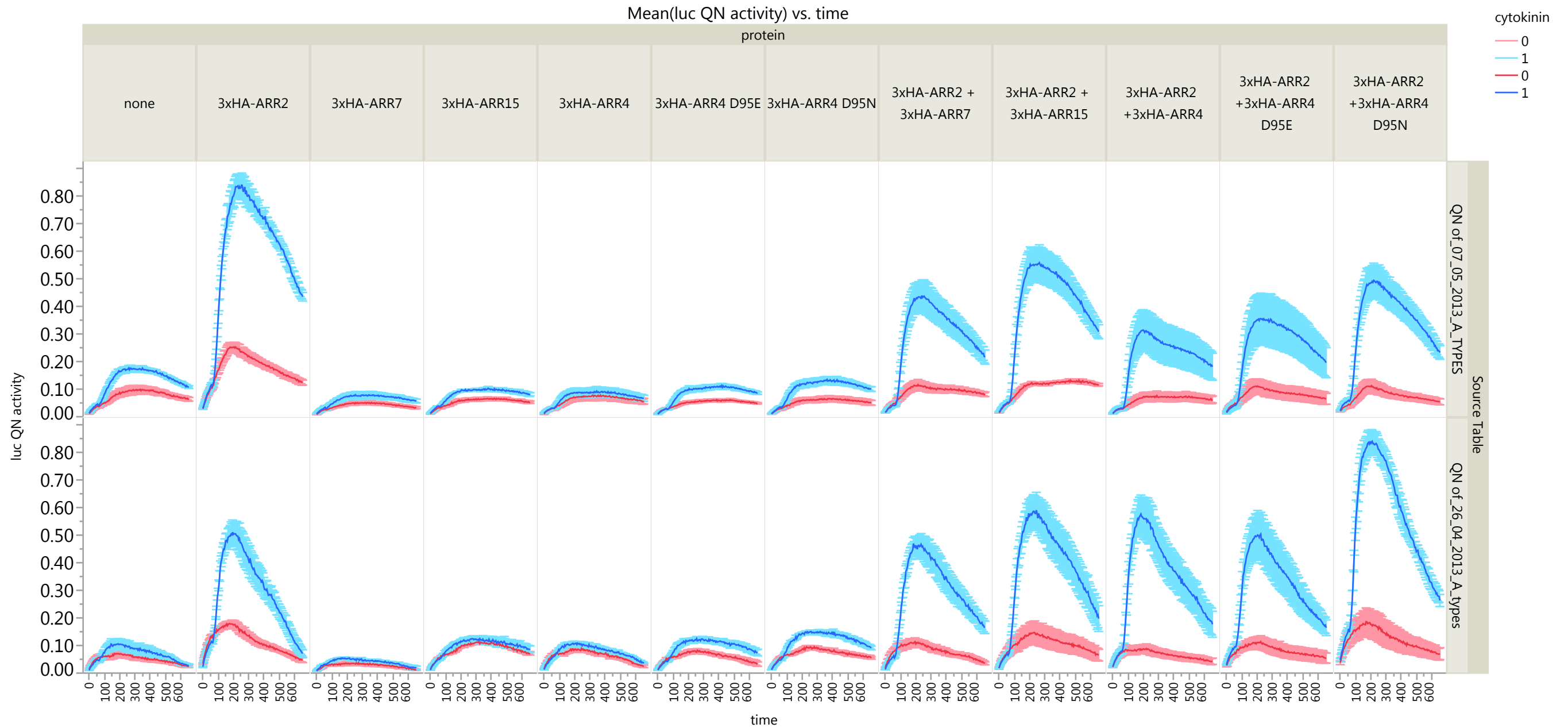

Each error bar is constructed using 1 standard error from the mean.

S12 Fig

Supplement: S12 Fig — Light emission curves are shown from two independent experiments comparing three A-types ARR4, ARR7, and ARR15, with ARR2. The active TCS-Asp of ARR4 was mutated to Asn (ARR4D95N) or Glu (ARR4D95E). As explained in the main body text, all A-types were able to block the cytokinin induction when singularly expressed, but were not able to block ARR2 overexpression effects as observed with ARR22. (PDF) [file pone.0212056.s012.pdf]

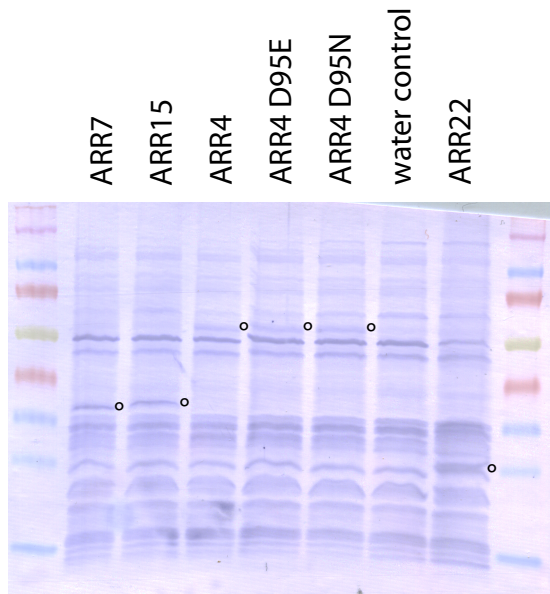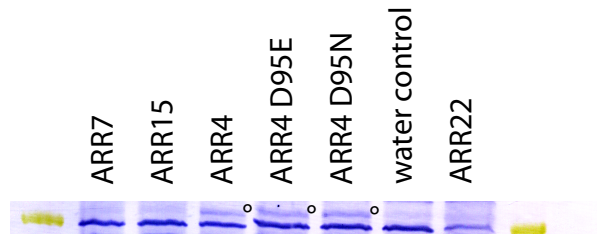

Contrast enhanced for ARR4

Primary antibody α-HA (ab-24779). ° indicates the specific protein band.

Supplement: S13 Fig — (PDF) [file pone.0212056.s013.pdf]

# Basal Expression Level

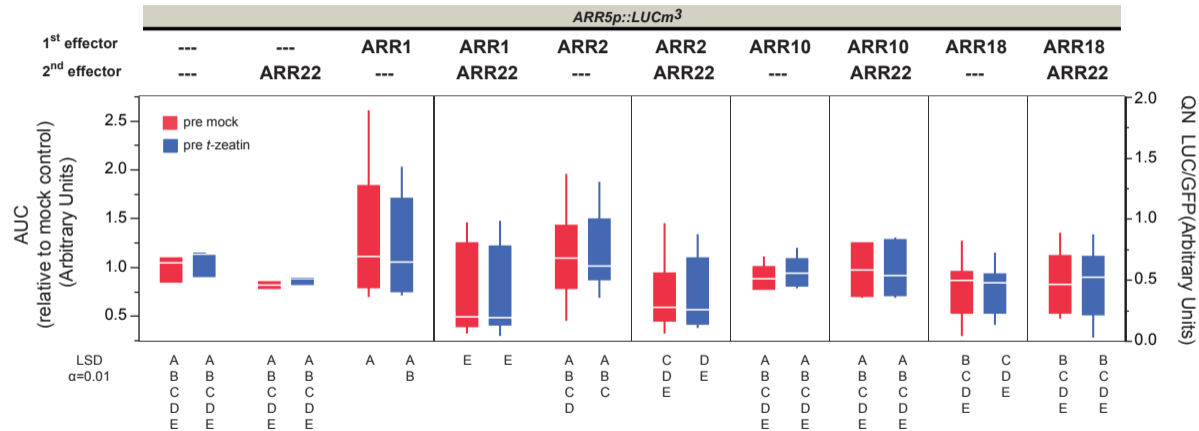

Supplement: S15 Fig — (PDF) [file pone.0212056.s015.pdf]

LUC/GFP & Mean(LUC/GFP) vs. Time[min]

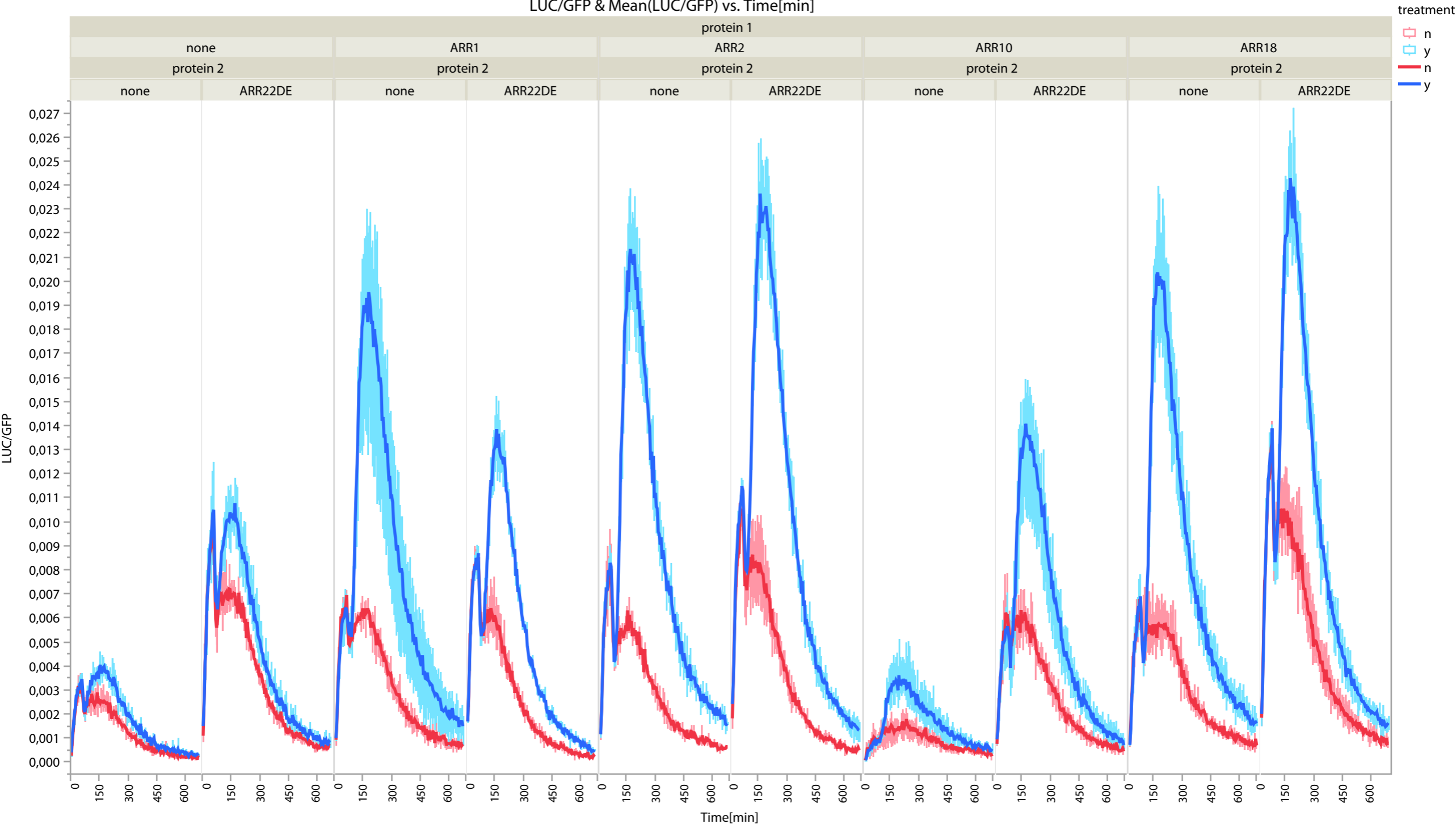

S17 Fig

Supplement: S17 Fig — Light emission curves are shown from co-transfection of the promoter-reporter along with ARR1, ARR2, ARR10 or ARR18 and ARR22D74E. Although some variance was seen, overexpression of ARR22D74E in any form did not block the constitutive-like effect of B-type overexpression. (PDF) [file pone.0212056.s017.pdf]

# Basal expression effect

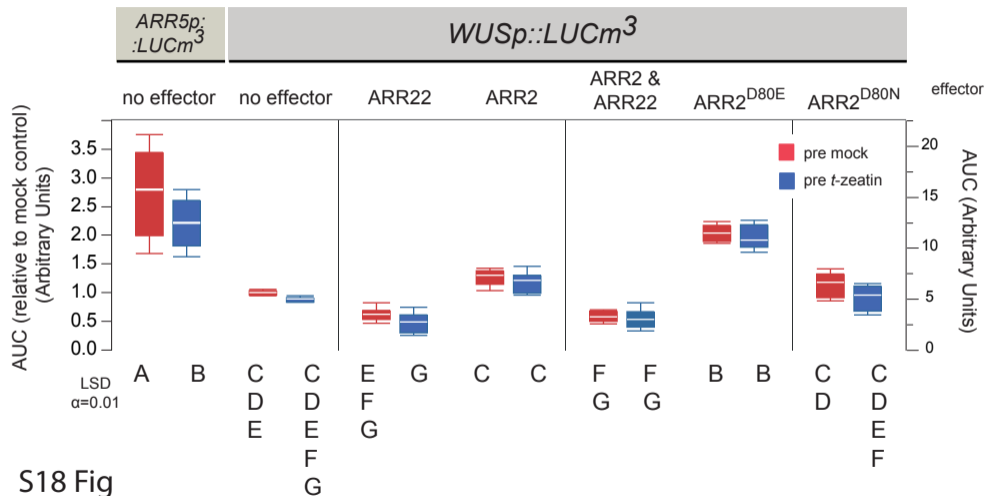

S18 Fig

Supplement: S18 Fig — (PDF) [file pone.0212056.s018.pdf]

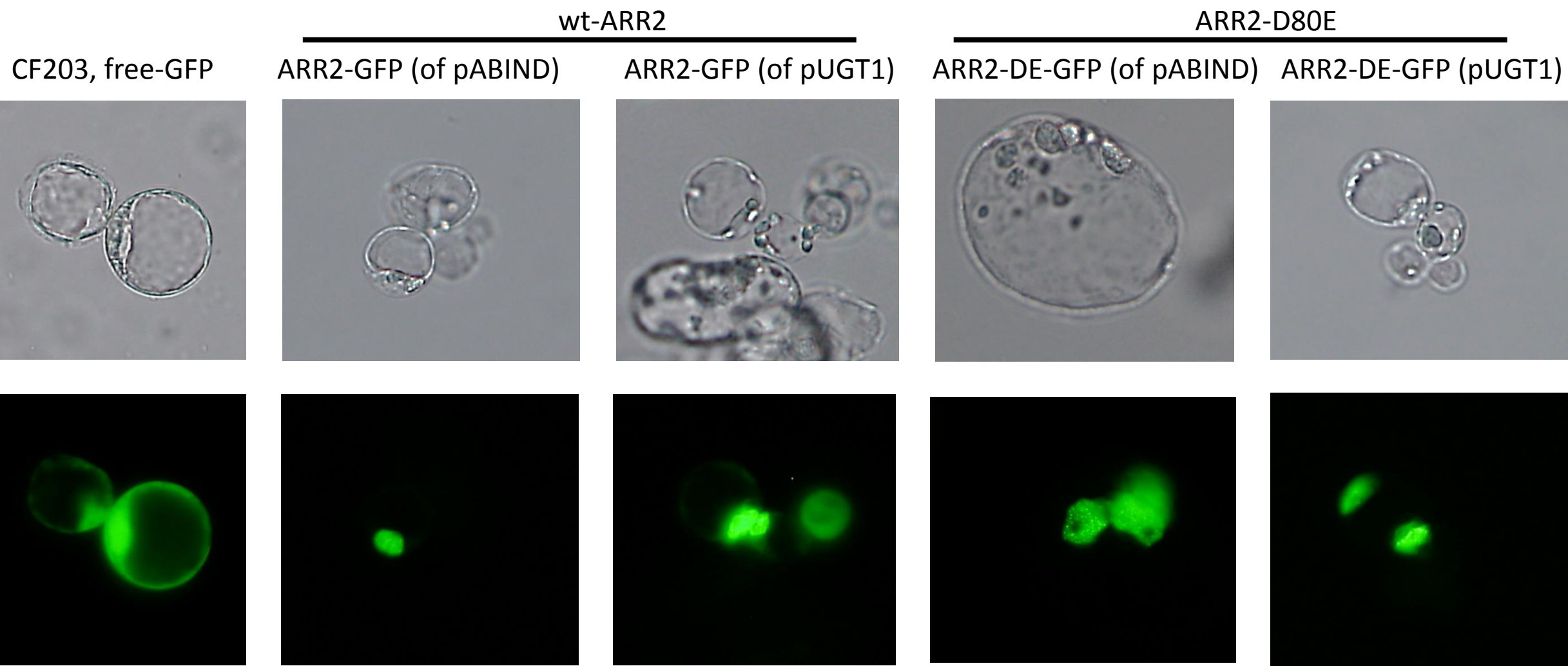

S20 Fig

Supplement: S20 Fig — (PDF) [file pone.0212056.s020.pdf]

A

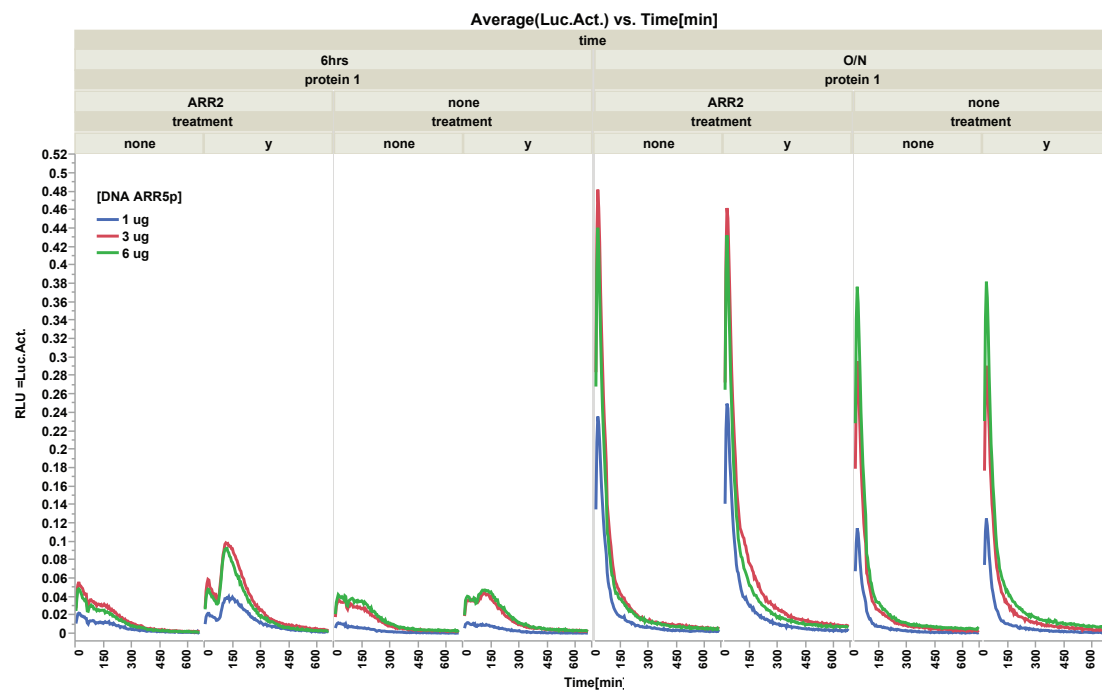

B

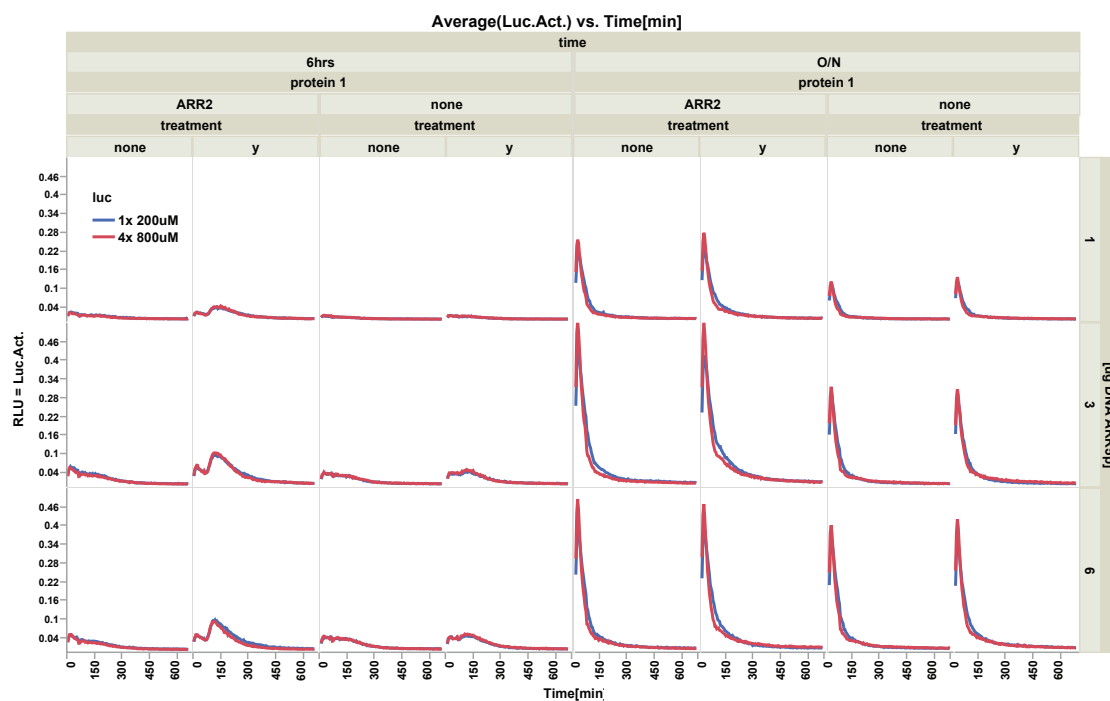

C

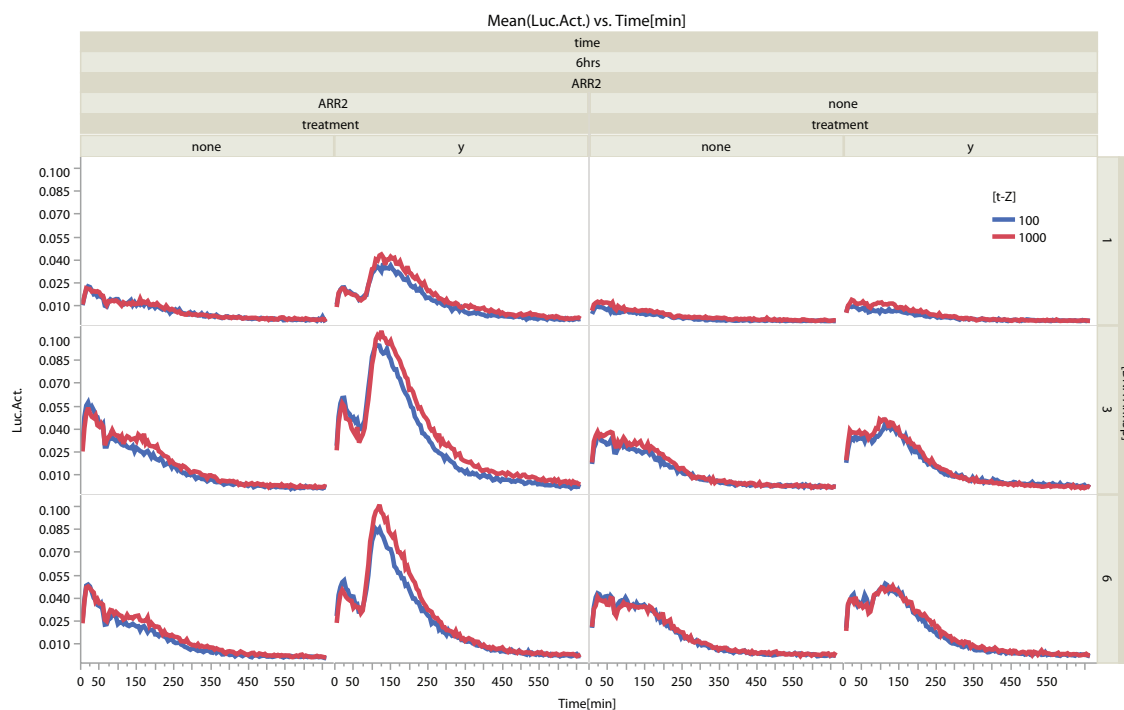

Supplement: S21 Fig — (A) Comparison of total light emission values with respect to the amount of reporter DNA. (B) Comparison of two different D-luciferin concentrations (C) Comparison of two different cytokinin concentrations (nM) and amount of reporter DNA. The assay conditions show that 3 μg reporter is saturating and that the substrate D-luciferin is not limiting. In addition, although overnight incubations led to higher levels of emission presumably due to accumulation of LUC protein the induction dynamism was lost. Shorter incubation times therefore resulted in better signal-to-noise ratios with respect to cytokinin treatments. (PDF) [file pone.0212056.s021.pdf]

# Sum(Luc.Act.) vs. cells uL

biorep

1  
2

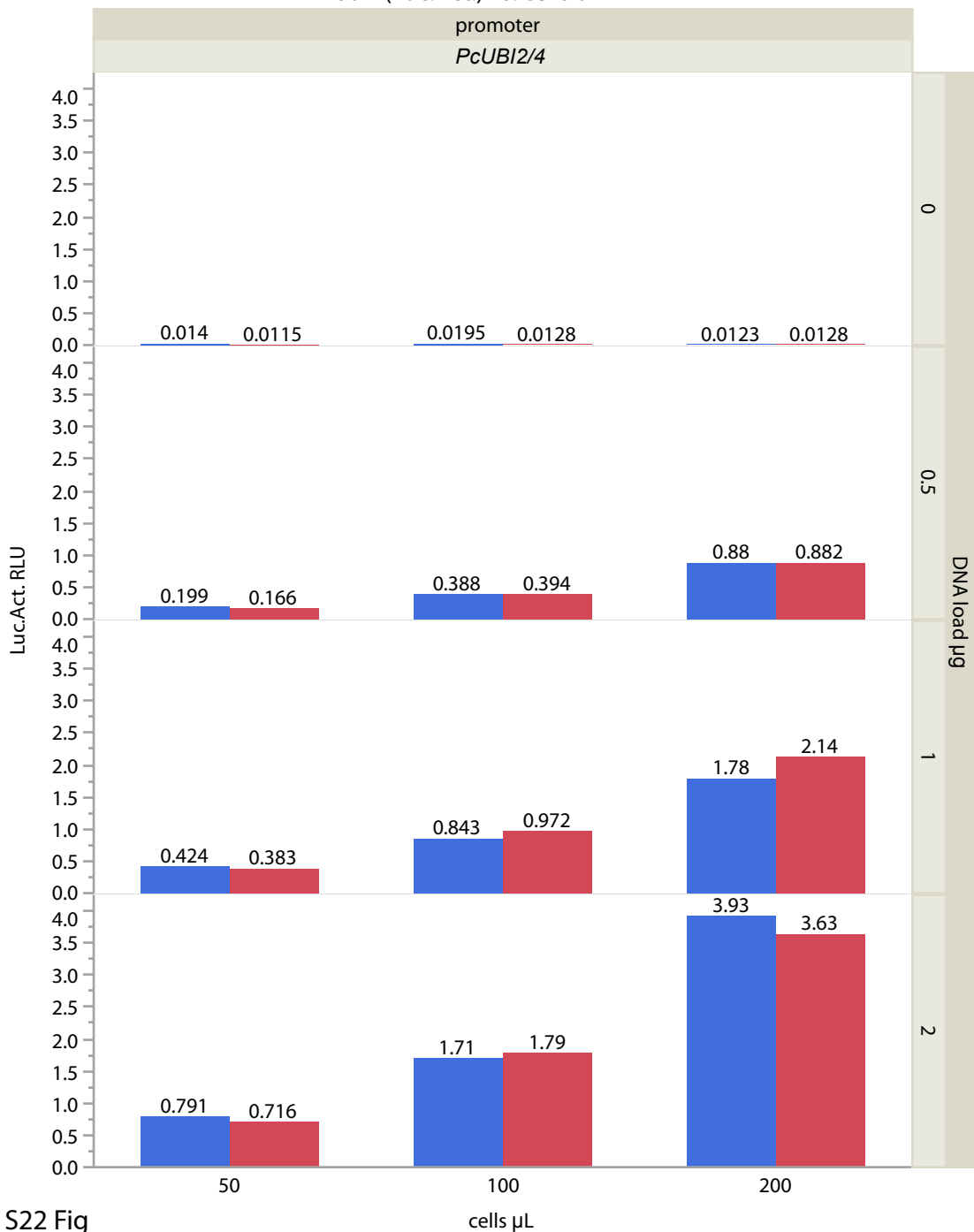

Supplement: S22 Fig — Cells were transfected with doubling amounts of plasmid DNA and assayed as described in the methods. Afterwards different amounts of cells from those transfections were distributed to microtiter wells and the in vivo emission was measured for one hour; two bio-replicates are shown. The sum of the emission is shown. The emission response doubled both in response to the number cells provided to the wells and also to the amount of DNA. This demonstrated that under these conditions and using a constitutive promoter we get a linear response in the number of cells emitting light due to the LUC reaction and is also linearly proportional to the DNA load. (PDF) [file pone.0212056.s022.pdf]

A

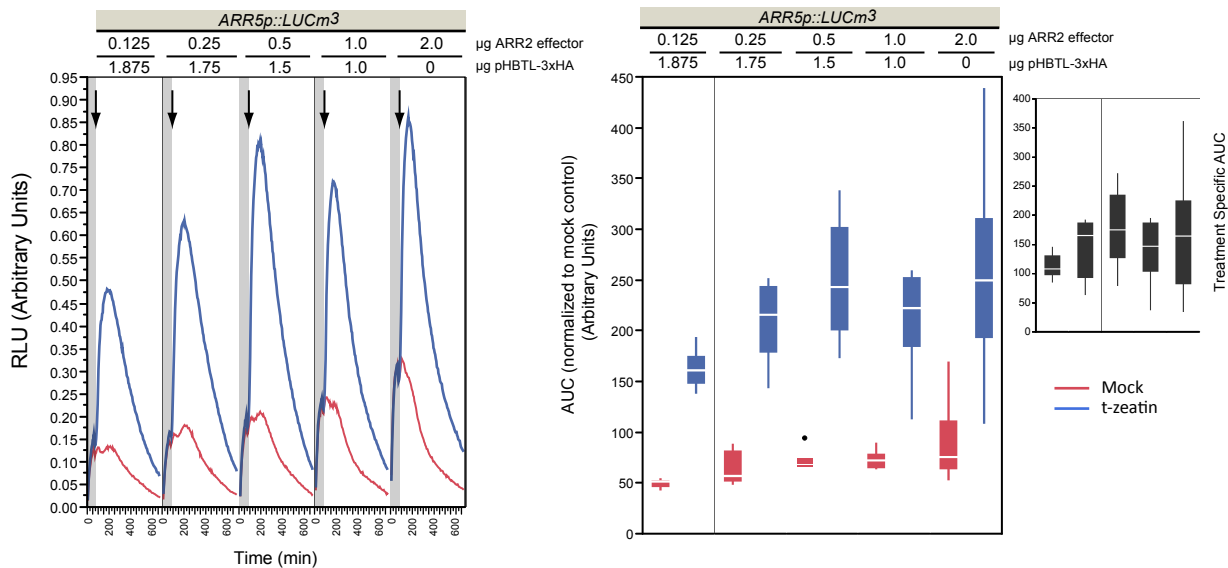

B

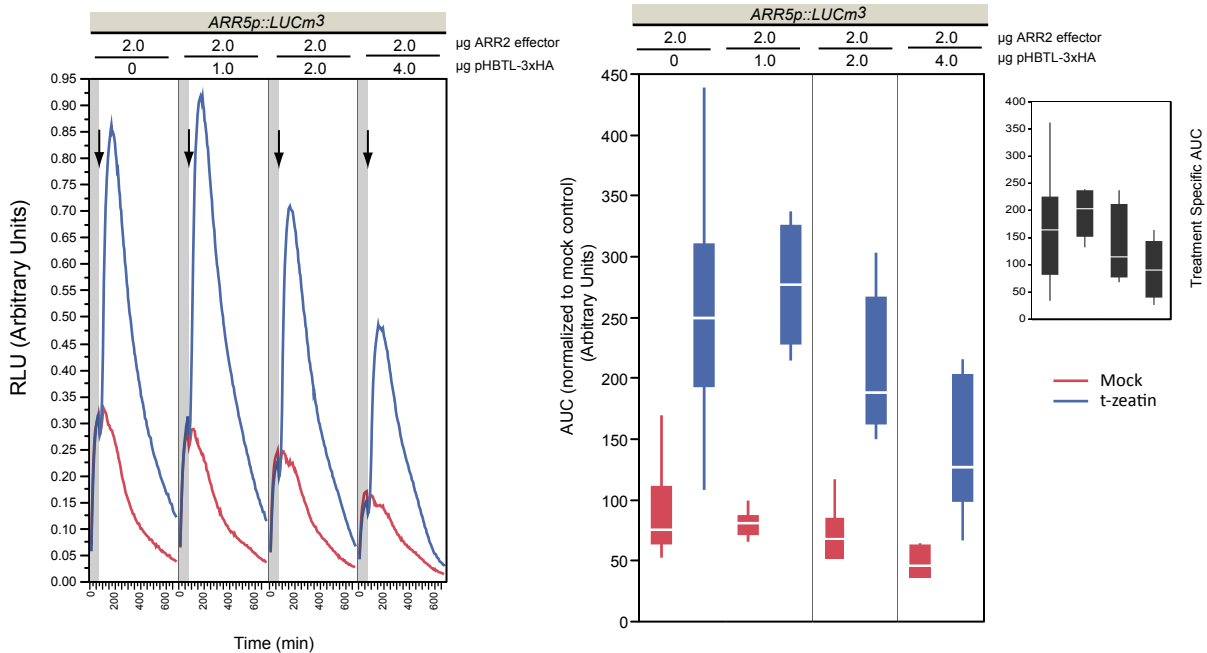

Supplement: S23 Fig — One aspect that is a concern for all protoplasts transfection experiments is the issue of unequal DNA loading. We are able to show that inter-plate variance is uniform (see S25 Fig). However, we were still concerned that DNA loading effects, which are reported occasionally in the literature, are a concern. Therefore two experiments were set up: (A) a test of the dynamic range of the reporter readout as a function of varying amounts of effector yet still under constant DNA load (here ARR2); and (B) the overall effect of adding more and more plasmid, as many of these experiments demand several DNA species. (A) In the experiments the reporter plasmids were set at a fixed 3 μg, the effectors at 2 μg. Here the total “effector” DNA load was kept at 2 μg by using the same backbone vector (“empty = pBHTL-3xHA”) used for all of the effectors (see Methods) and as in all other experiments the reporter was 3 μg, yet the effector ARR2 was varied from 2 μg down to 125ng of DNA. In principle similar response curves were obtained when 3xHA-ARR2 was co-transfected at 0.5 to 2 μg, see in the emission curves, the total AUC and also in the specific activity. Below this level (250 ng, 125 ng) a loss in the dynamic response was observed. In conclusion, the data support the hypothesis that the overexpression of ARR2 is likely maxed out and that differences we observed were not due to insufficient loading. (B) Here, the effect of adding more and more DNA to a fixed amount of effector (ARR2, 2 μg) and reporter (3 μg) was explored. Basically, the tolerance was only 1 μg extra DNA, after which the total response range went down. Therefore, in any experiment where multiple plasmids were used, the largest combination set the total DNA load and this load was met with the empty effector backbone vector. All of the effectors were cloned into the same vector, pBHTL-3xHA, therefore differences in three-dimensional typology of the vector should not play a role in our experiments here. (PDF) [file pone.0212056.s023.pdf]

RLU RAW

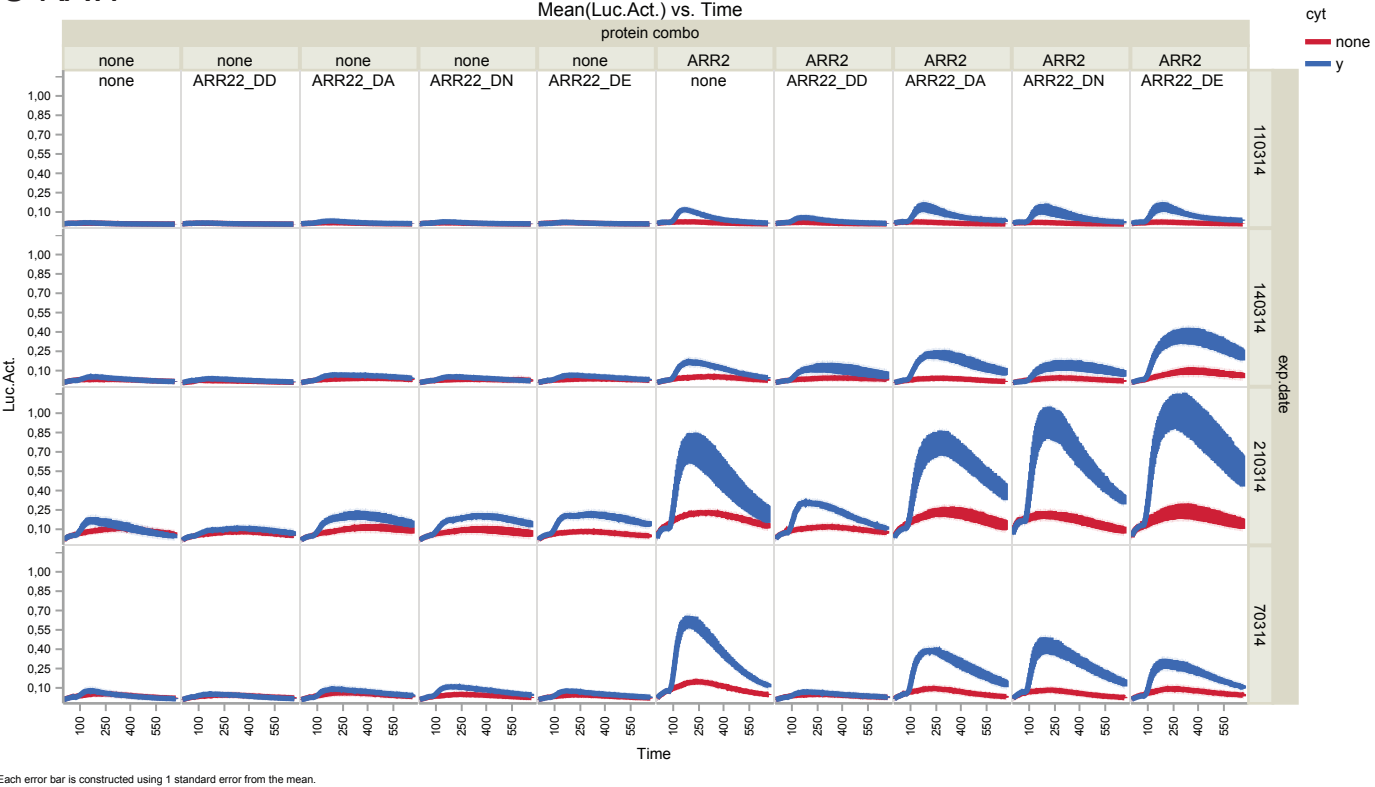

RLU Quantile Normalized

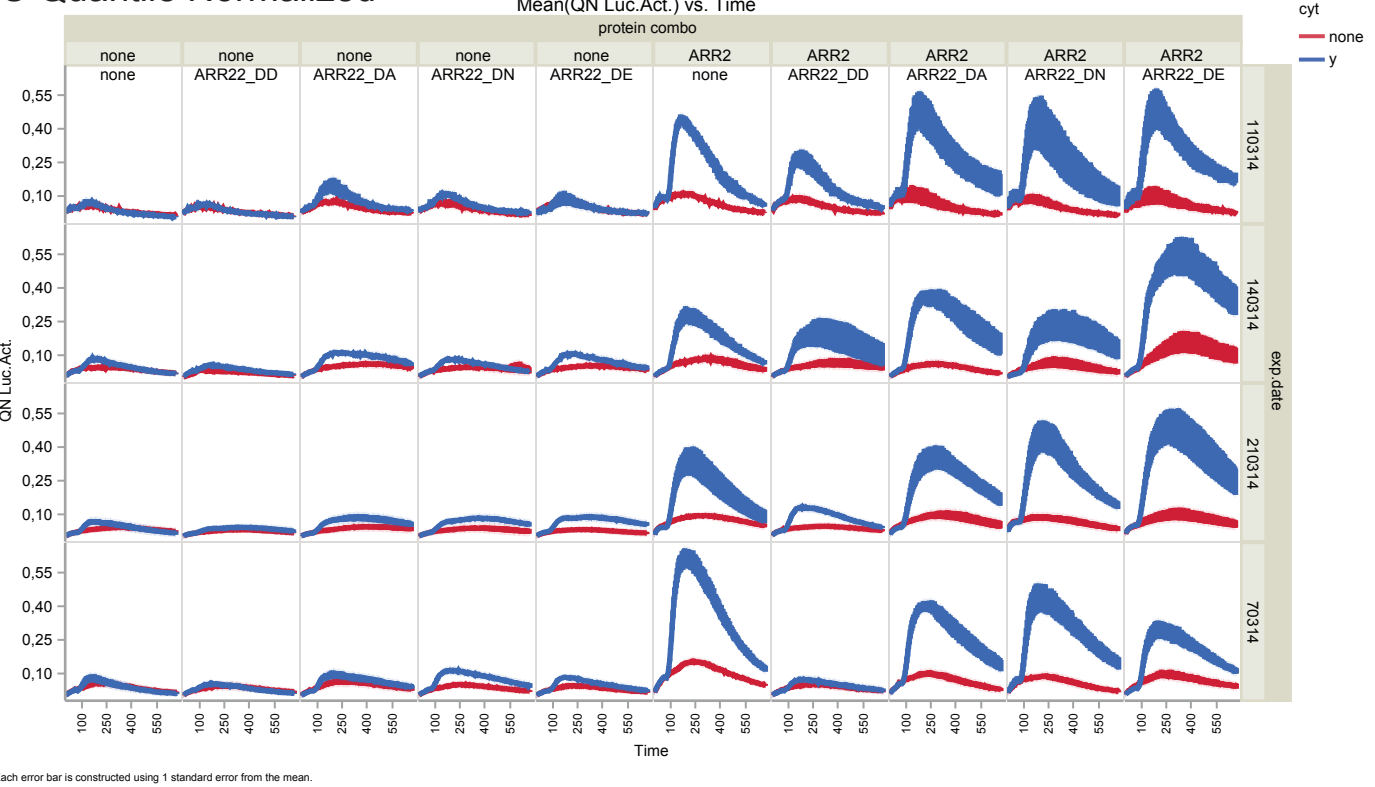

RLU variance  
before and after  
Quantile Normalization

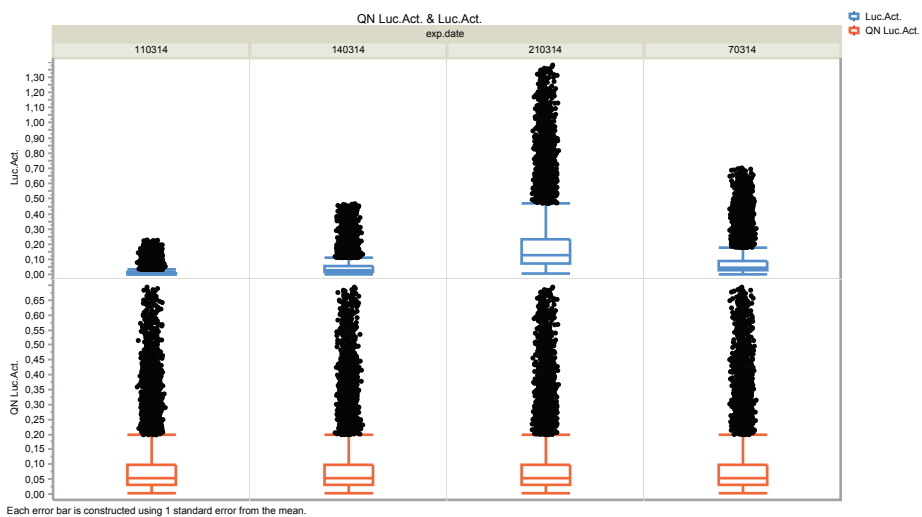

Supplement: S26 Fig — The raw data light emission curves (top) are followed by the quantile normalized datasets (middle). Notice that that the inter-plate variance was removed (bottom) without destroying the intra-plate variance (compare before and after quantile normalization). In this way, the total experimental variance was captured instead of using the “representative experiment” approach. However, we see that even when the total transfection levels varied, the representative approach would yield very similar results. Therefore, representative experiments were individually evaluated and are included in the S1 Dataset; all other data sets are available on request. (PDF) [file pone.0212056.s026.pdf]

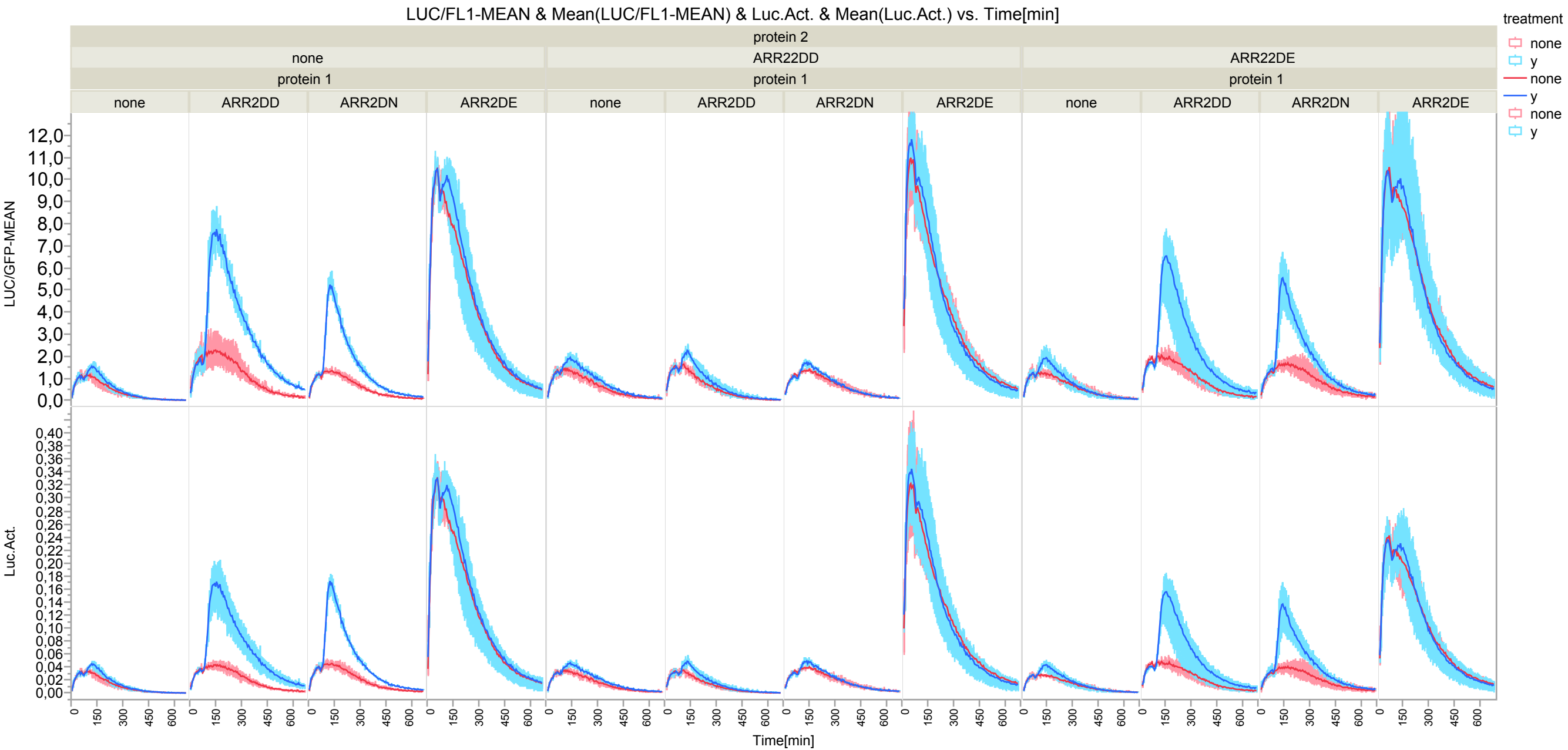

Supplement: S27 Fig — Variance is shown as boxplots and the mean overlaid. (PDF) [file pone.0212056.s027.pdf]

GFP emission TECAN

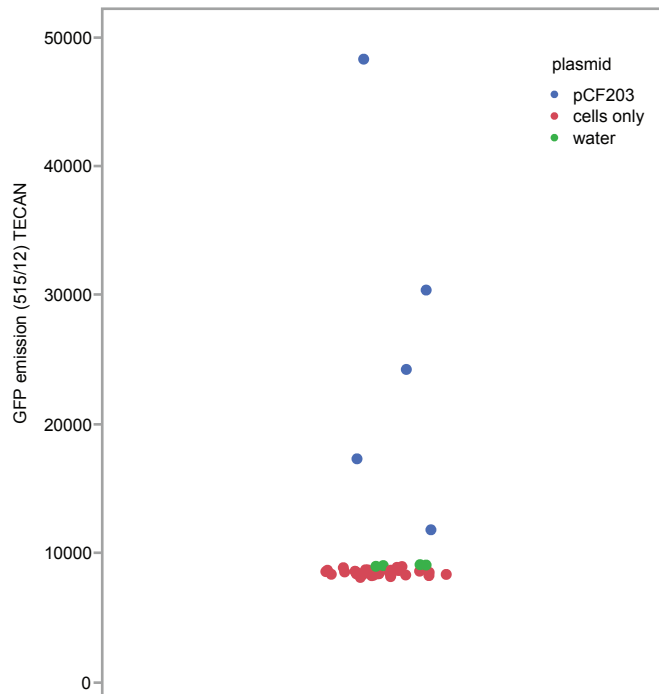

### Comparison between GFP intensity in TECAN and CytoFLEX

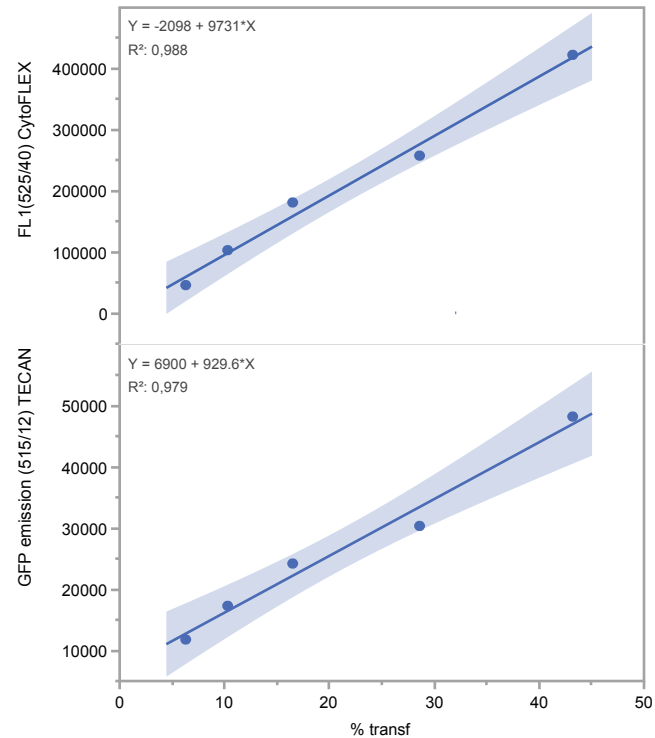

GFP emission TECAN

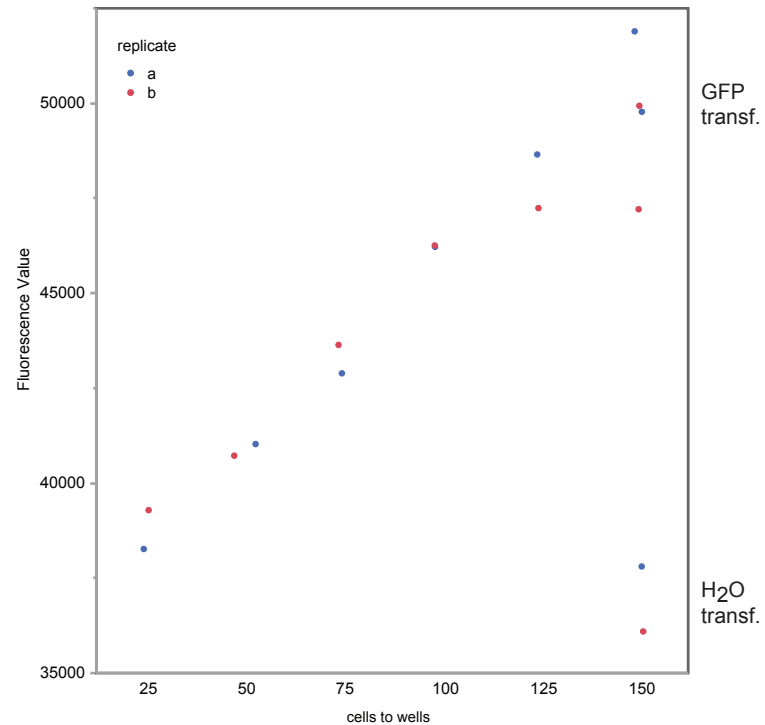

Supplement: S28 Fig — (A) GFP emission obtained from empty cells, cells transfected with water only and cells transfected with varying amounts of plasmid encoding GFP (pCF203). Only cells transfected with GFP have emission above that of the plate autofluorescence indicating that the cellular autofluorescence does not contribute the emission signal. (B) Comparison of the GFP emission from those cells transfected with GFP in (A) between the microplate reader and a cytometer graphed against the number of cells expressing GFP quantified using the cytometer. A direct and linear correlation with the number of transfected with the emission intensity is obtained by both methods. C) Cells were transfected with 2μg pCF203 and the diluted in K3 and distributed to various wells in a microtiter plate. We obtained a decent linear relationship with the number of cells and the GFP emission with a minimal of 75 μL of cells needed to have a signal securely above the plate autofluorescence. This indicates that under these conditions one can use this set up to monitor the transfection rate variance and the cell distribution variance by monitoring in vivo GFP emission. See Methods to obtain the settings for the microplate reader and the cytometer. (PDF) [file pone.0212056.s028.pdf]
