# Supplementary material for: ARR22 overexpression can suppress plant Two-Component Regulatory Systems
Source: PLoS One. 2019 Feb 11;14(2):e0212056. doi: 10.1371/journal.pone.0212056 (PMC6370222; doi:10.1371/journal.pone.0212056)
Supplement: S3 Fig — A structural alignment was performed using PROMALS3D (PROfile Multiple Alignment with predicted Local Structures and 3D constraints) http://prodata.swmed.edu/promals3d/promals3d.php. ARR2 protein sequence was obtained from TAIR (www.arabidopsis.org) and aligned to the E. coli structures of PhoB (PDB: 1B00, https://www.rcsb.org/structure/1b00) and CheY (PDB: 2CHF, https://www.rcsb.org/structure/2chf). The conserved acidic pocket residues of the REC domain require a divalent cation (e.g. Mg2+, shown as a pink circle), are required to enable the phosphotransfer reaction (Bourret, 2010). The conserved Asp that is nominally phosphorylated is indicated with a ~P blue circle (Bourret, 2010). The structural alignment shows that the ARR2 REC domain is predicted to have the same structure as that of CheY, that is following the same five (αβ)5 structure hallmark of response regulators (Bourret, 2010). Bourret RB. Receiver domain structure and function in response regulator proteins. Curr Opin Microbiol. 2010;13(2):142–9. Epub 2010/03/10. (PDF) [file pone.0212056.s003.pdf]

PROMALS3D alignment (sequences in aligned order)
http://prodata.swmed.edu/promals3d/promals3d.php

```
Conservation:                                           999 9           9
1b00_chainA_PhoB      1   --------------------------ARRILVVEDEAPIREMVCFVLEQNGF-QPVEAED    33
2chf_chainA_CheY      1   ------------------------ADKELKFLVVDDFSTMRRIVNLLKELGFNNVEEAED    37
ARR2_REC              1   MVNPGHGRGPDSGTAAGGSNSDPFPANLRVLVVDDDPTCLMILERMLMTCLY-RVTKCNR    59
Consensus_aa:             ............................+hLVV-D.ssh..hl..hLbp..@.ps.ctpc
Consensus_ss:                                         eeeee   hhhhhhhhhhhhh   eeeee

Conservation:                9   9            9   9   9                99 9  9
1b00_chainA_PhoB     34   YDSAVNQLNEPW--PDLILLDWMLPGGSGIQFIKHLKRESMTRDIPVVMLTARGEEEDRV    91
2chf_chainA_CheY     38   GVDALNKLQAGG--FGFIISDWNMPNMDGLELLKTIRADSAMSALPVLMVTAEAKKENII    95
ARR2_REC             60   AESALSLLRKNKNGFDIVISDVHMPDMDGFKLLEHVGLEM---DLPVIMMSADDSKSVVL   116
Consensus_aa:             ..sAlsbLp.s....shll.Dh.hPs.sGhphlchl..-....slPVlMhoAcspcps.l
Consensus_ss:             hhhhhhhhhh      eeeee       hhhhhhhhhh        eeeee    hhhhh

Conservation:                  99  9  99     9
1b00_chainA_PhoB     92   RGLETGADDYITKPFSPKELVARIKAVMRRI----------------------   122
2chf_chainA_CheY     96   AAAQAGASGYVVKPFTAATLEEKLNKIFEKLGM--------------------   128
ARR2_REC            117   KGVTHGAVDYLIKPVRIEALKNIWQHVVRKKRNEWNVSEHSGGSIEDTGGDRD   169
Consensus_aa:             .thphGAssYlhKPhp...L...hp.lhc+b......................
Consensus_ss:             hhhh     eee    hhhhhhhhhhhhhh
```

important residues conserved in response regulators

conserved residues identified in this alignment

Mg2+ The conserved acidic pokect residues that chelate a divalent metal ion essential for all phosphoryal group chemistry.

~P Typical position of the mapped phosphorylated Asp of the acidic pocket.

Receiver domain structure and function in response regulator proteins. Bourret RB. Curr Opin Microbiol. 2010 Apr;13(2):142-9. doi: 10.1016/j.mib.2010.01.015.

S3 Fig
