# Supplementary material for: ARR22 overexpression can suppress plant Two-Component Regulatory Systems
Source: PLoS One. 2019 Feb 11;14(2):e0212056. doi: 10.1371/journal.pone.0212056 (PMC6370222; doi:10.1371/journal.pone.0212056)
Supplement: S14 Fig — Published protein-protein interactions between the proteins used in this study were obtained from the bioBIND database and two graphs constructed with Cystoscope show the interaction method (A) and their corresponding studies (B). References with their Pubmed ID (PMID) are given in the Figure. (PDF) [file pone.0212056.s014.pdf]

A

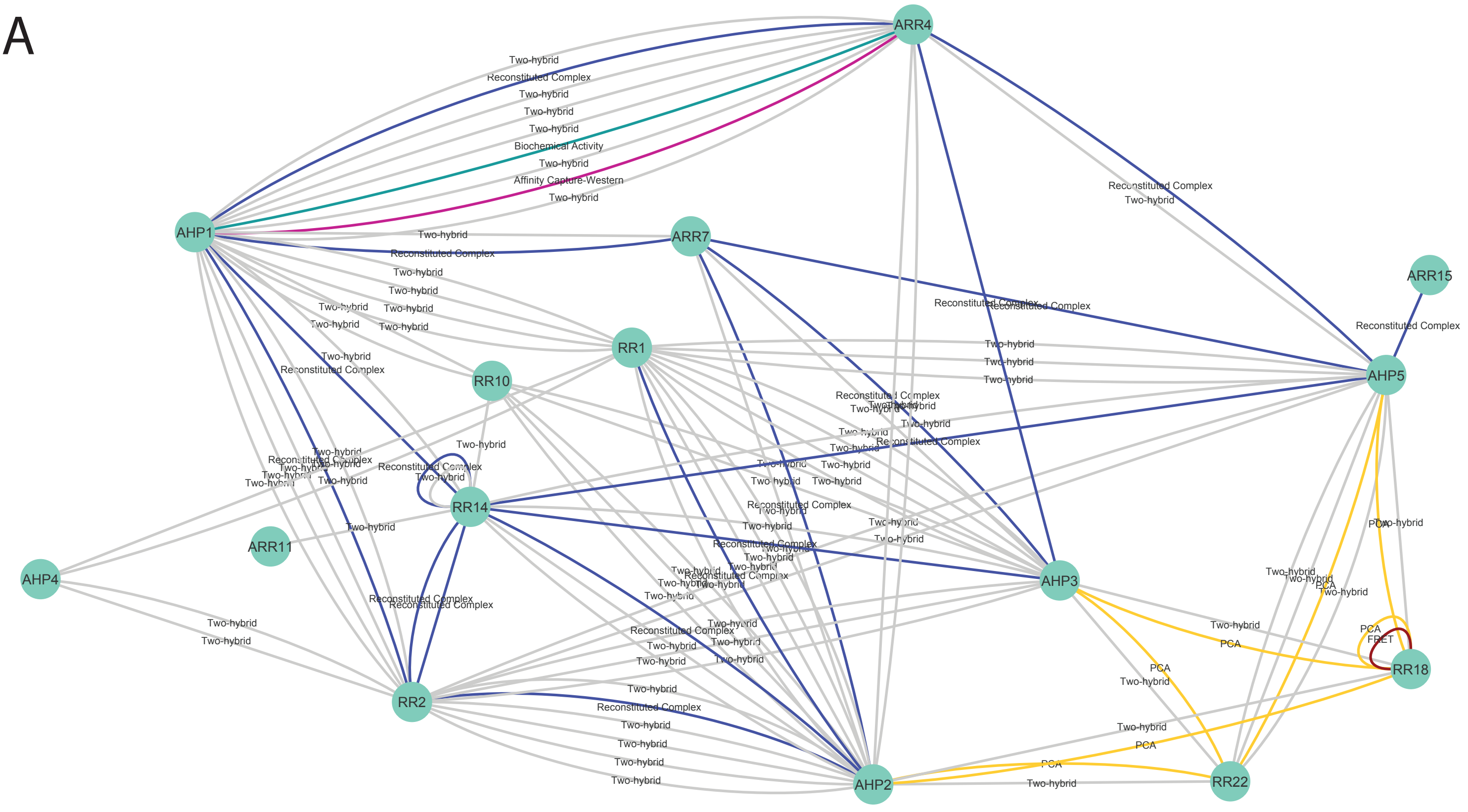

B

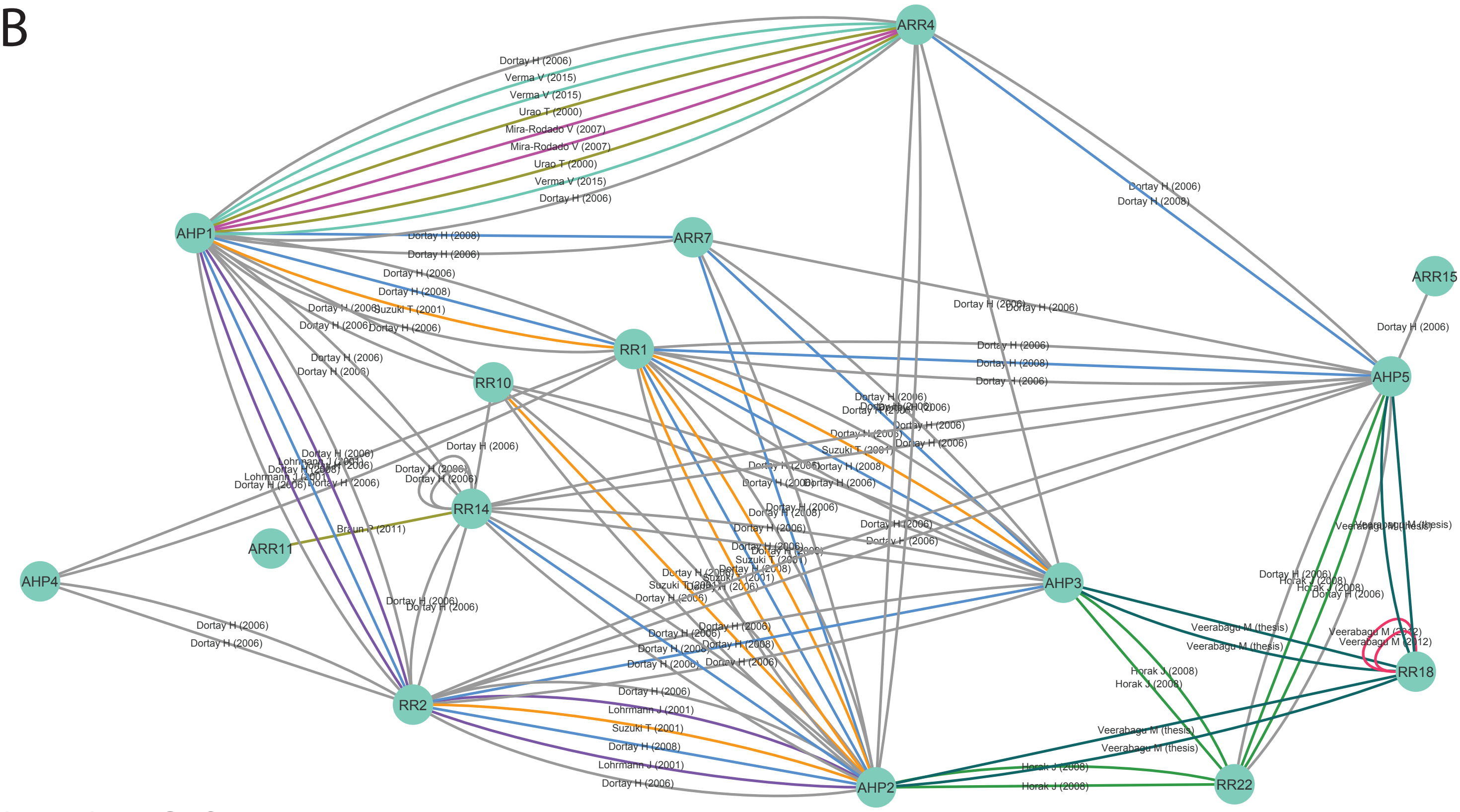

REFERENCES

(Braun P (2011), PMID 21798944; Cutcliffe JW (2011), PMID 21705390; Dortay H (2006), PMID 16965536; Dortay H (2008), PMID 18642946; Efroni I (2013), PMID 23449474; Horak J (2008), PMID 18625081; Kim HJ (2013), PMID 23720308; Klopffleisch K (2011), PMID 21952135; Lohrmann J (2001), PMID 11370868; Manzano C (2008), PMID 18535787; Marin-de la Rosa N (2014), PMID 25118255; Mira-Rodado V (2007), PMID 17545225; Suzuki T (2001), PMID 11158442; Sweere U (2001), PMID 11691995; Urao T (2000), PMID 10930573; Veerabagu M (2012), PMID 22775331; Veerabagu M (2014), PMID 24948556; Verma V (2015), PMID 25643735; Wang Y (2011), PMID 21699589; Yamada H (1998), PMID 9771897). PMID = unique PubMed record Identifier number.

Graph made in Cytoscape (PMID:14597658) with data supplemented from BioGRID: https://thebiogrid.org/
Edges with methods (A) directly correspond to their references (B) based on their position.

S14 Fig
