# Supplementary material for: ARR22 overexpression can suppress plant Two-Component Regulatory Systems
Source: PLoS One. 2019 Feb 11;14(2):e0212056. doi: 10.1371/journal.pone.0212056 (PMC6370222; doi:10.1371/journal.pone.0212056)
Supplement: S16 Fig — (PDF) [file pone.0212056.s016.pdf]

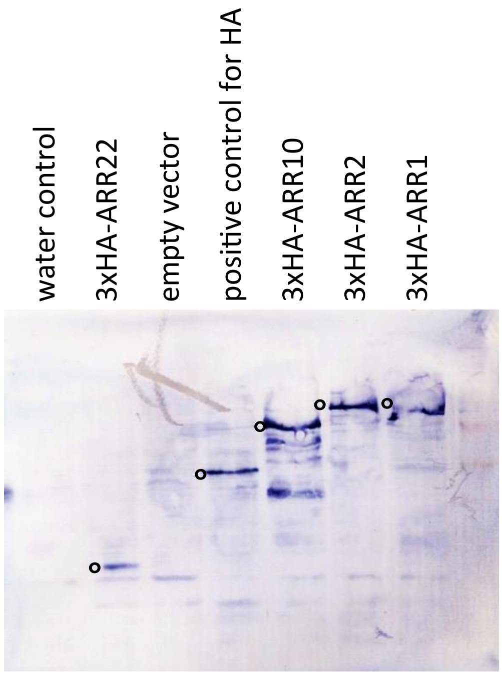

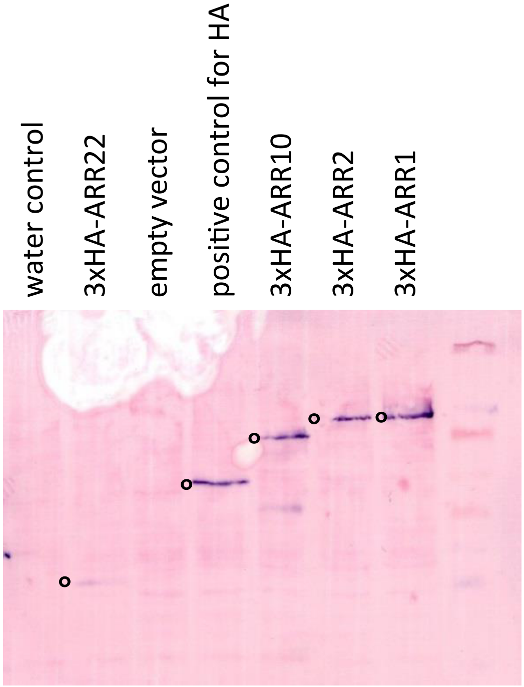

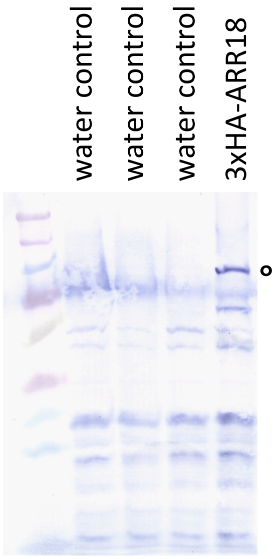

Specific Detection with α-HA-rat/α-rat-AP/NBT-BCIP detection.
° denotes specific protein band.

S16 Fig
