# Supplementary material for: ARR22 overexpression can suppress plant Two-Component Regulatory Systems
Source: PLoS One. 2019 Feb 11;14(2):e0212056. doi: 10.1371/journal.pone.0212056 (PMC6370222; doi:10.1371/journal.pone.0212056)
Supplement: S19 Fig — Immunoblot analysis of ARR2 variants co-transfectred along with the WUS promoter. This immunoblot was run with other samples which are not relevant for the current manuscript and were removed from the image; all samples are from the same blot and experimental run. (PDF) [file pone.0212056.s019.pdf]

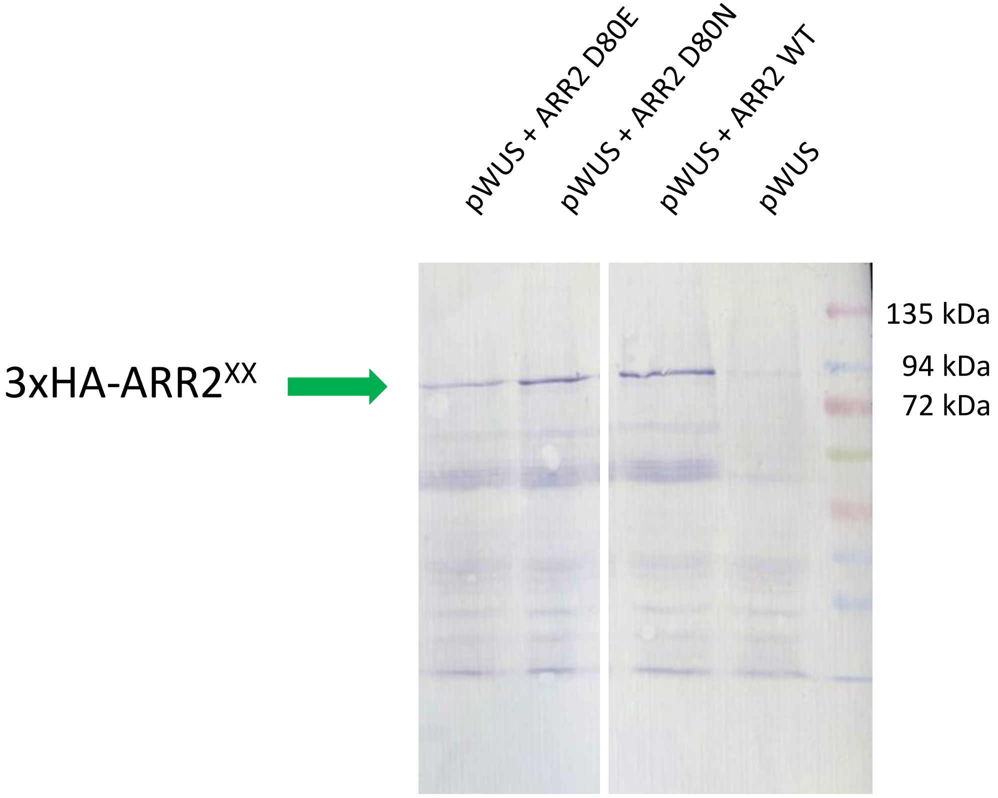

Specific Detection with α-HA-rat/α-rat-AP/NBT-BCIP

S19 Fig
