# Supplementary material for: ARR22 overexpression can suppress plant Two-Component Regulatory Systems
Source: PLoS One. 2019 Feb 11;14(2):e0212056. doi: 10.1371/journal.pone.0212056 (PMC6370222; doi:10.1371/journal.pone.0212056)
Supplement: S24 Fig — Each biological replicate is split into two technical replicates to catch subsampling errors. The biological replicates captures variance between transfections. Grey dots: where transfections occur in 96-deep well plates. Arrows show splitting into LUC microtiter plate. Green dot shows the position of transfection control (in this case using GFP and flow cytometry, see S25 Fig). Promoter-effector, bio-technical replicates, and mock or cytokinin treatments are shown as color blocks. 30 μL transfections make one mock and one treatment sample and 60 μL transfections yield two mock and two treatment samples. (PDF) [file pone.0212056.s024.pdf]

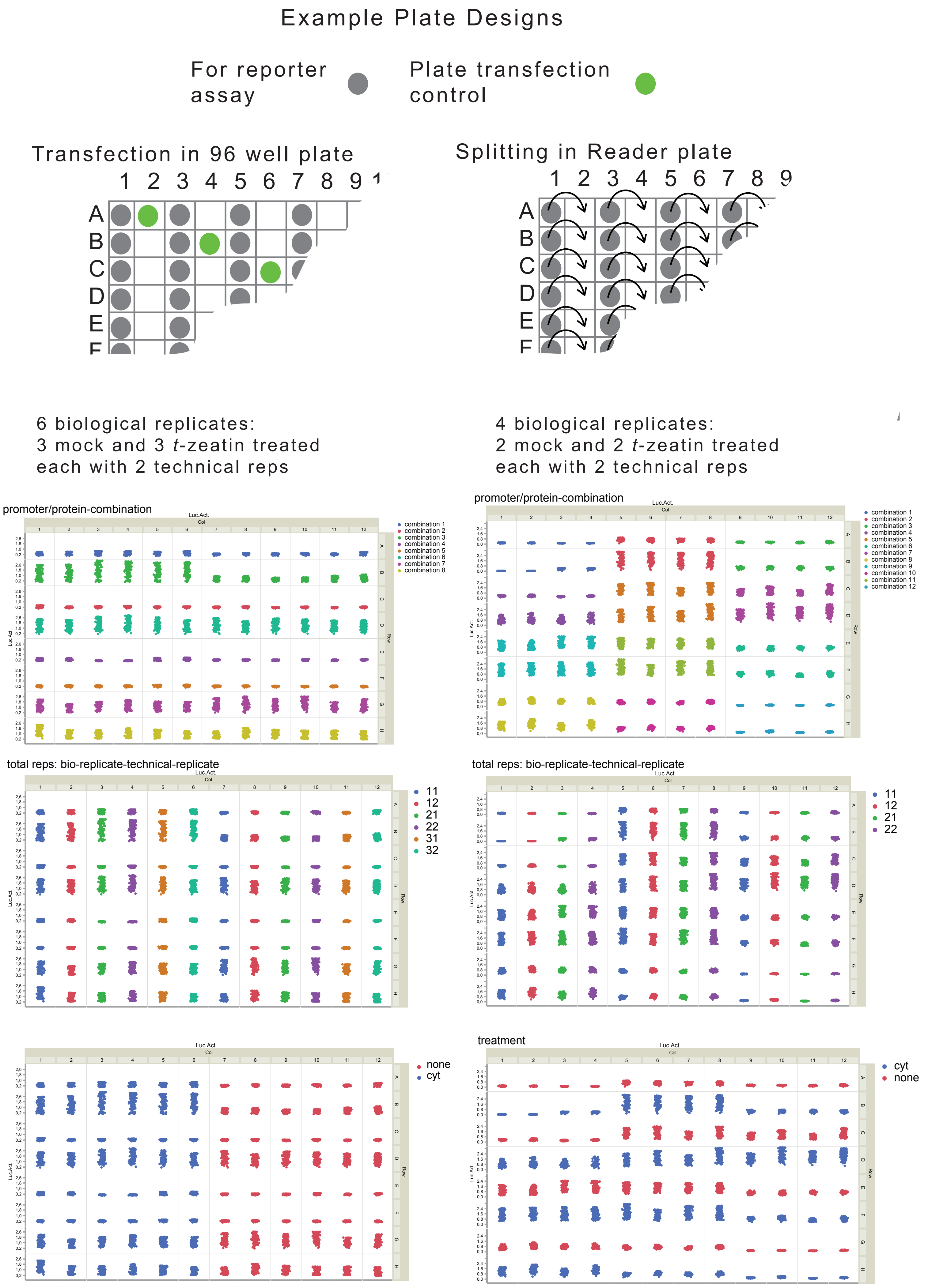

Example Plate Designs
For reporter assay
Plate transfection control
Transfection in 96 well plate
Splitting in Reader plate
6 biological replicates:
3 mock and 3 t-zeatin treated
each with 2 technical reps
4 biological replicates:
2 mock and 2 t-zeatin treated
each with 2 technical reps
promoter/protein-combination
total reps: bio-replicate-technical-replicate
treatment
Luc.Act.
Col
Row
none
cyt

S24 Fig
