# Supplementary material for: ARR22 overexpression can suppress plant Two-Component Regulatory Systems
Source: PLoS One. 2019 Feb 11;14(2):e0212056. doi: 10.1371/journal.pone.0212056 (PMC6370222; doi:10.1371/journal.pone.0212056)
Supplement: S25 Fig — Since the biological replicates are split, the remaining space on the plates could be used to monitor the transfection efficiency across the plate (green dots). An example sampling is shown here including the flow cytometric output. Here were see that on this particular day the GFP reference gave about 8% at any position across the plate. We conclude that the entire plate had an even transfection as expected. Of course the total transfection rates can vary depending on batch variation (see S26 Fig for an example); but we can be sure that per plate the total transfection rate will be similar using this method. (PDF) [file pone.0212056.s025.pdf]

For reporter assay

Plate transfection control

Transfection in 96 well plate

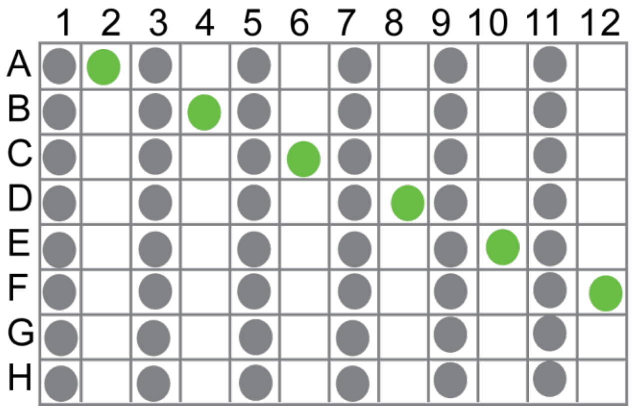

Splitting in Reader plate

Well	% cells with GFP
A2	8.1 ± 0.2
B4	7.0 ± 0.3
C6	8.0 ± 0.0
D8	7.6 ± 0.1
E10	8.3 ± 0.1
F12	7.8 ± 0.5

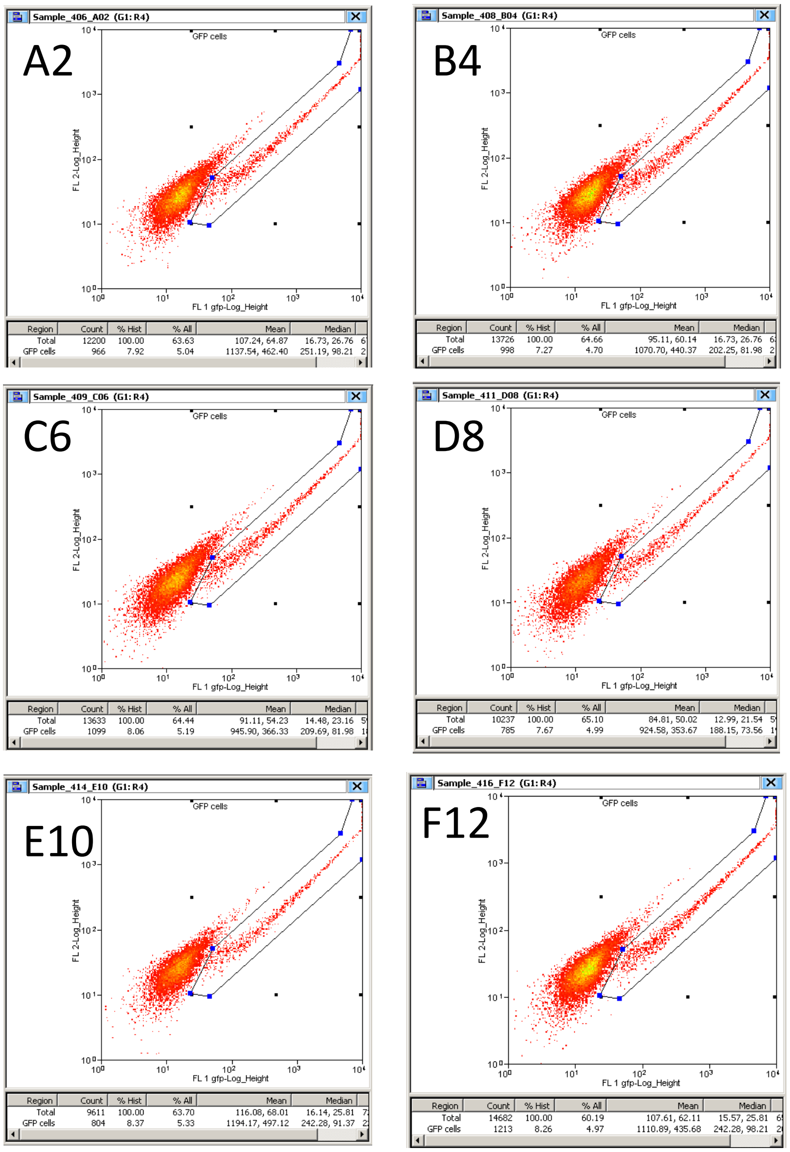

S25 Fig
