# Supplementary material for: ARR22 overexpression can suppress plant Two-Component Regulatory Systems
Source: PLoS One. 2019 Feb 11;14(2):e0212056. doi: 10.1371/journal.pone.0212056 (PMC6370222; doi:10.1371/journal.pone.0212056)
Supplement: S29 Fig — A FACS-RT-qPCR experiment was conducted 6 hours after transfection following the same procedure as for Fig 8 for genes ARR1, ARR2, ARR5, ARR7 and EF2. As in Fig 8, effector plasmids encoding ARR2-eGFP, ARR2D80E-eGFP or free-eGFP were transfected into protoplasts, either mock (-) or treated with 1 μM t-zeatin (+). Each effector treated sample was normalized to the median of EF2 with differences shown in Ct(Cq) with respect to EF2 (log2). Smaller values indicate a higher transcript rate. (PDF) [file pone.0212056.s029.pdf]

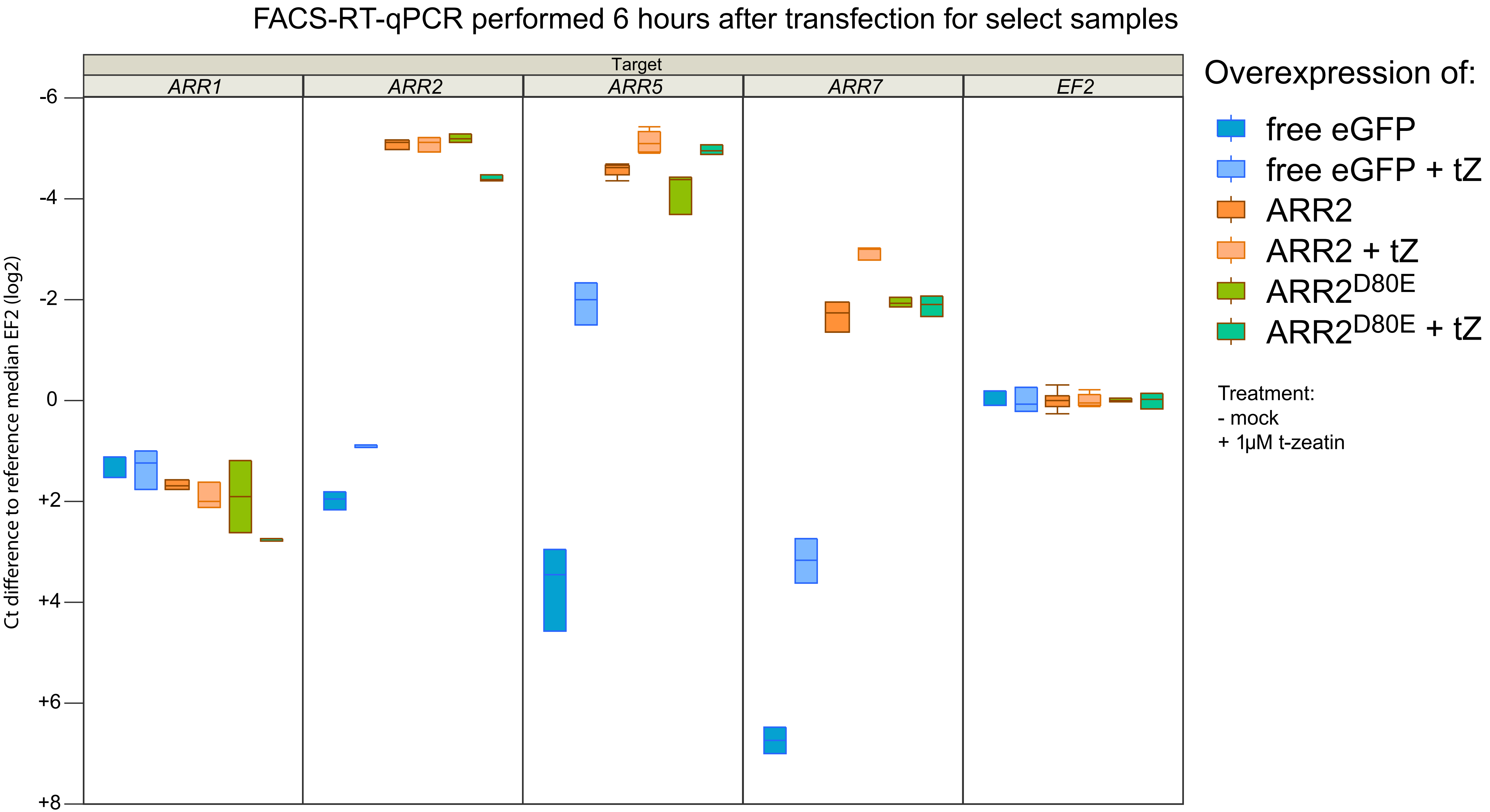
FACS-RT-qPCR performed 6 hours after transfection for select samples
Target
ARR1
ARR2
ARR5
ARR7
EF2
Ct difference to reference median EF2 (log2)
-6
-4
-2
0
+2
+4
+6
+8
Overexpression of:
free eGFP
free eGFP + tZ
ARR2
ARR2 + tZ
ARR2D80E
ARR2D80E + tZ
Treatment:
- mock
+ 1µM t-zeatin

S29 Fig
