# Supplementary material for: ARR22 overexpression can suppress plant Two-Component Regulatory Systems
Source: PLoS One. 2019 Feb 11;14(2):e0212056. doi: 10.1371/journal.pone.0212056 (PMC6370222; doi:10.1371/journal.pone.0212056)
Supplement: S1 Table — A total of three biological replicates with each three technical replicates yield nine replicates. Observe that the positive qPCR calls for WUS was on average 4/9 with free eGFP or overexpression of ARR2, but was 8/9 when ARR2D80E was overexpressed. (PDF) [file pone.0212056.s030.pdf]

	Overexpression of					
	ARR2 - tZ	ARR2 + tZ	ARR2^{D80E} tZ-	ARR2^{D80E} + tZ	free eGFP - tZ	free eGFP + tZ
	qPCR Call present	qPCR Call present	qPCR Call present	qPCR Call present	qPCR Call present	qPCR Call present
Target	Sum	Sum	Sum	Sum	Sum	Sum
ACT2	9	9	9	9	9	9
ARR1	9	9	9	9	9	9
ARR2	9	9	9	9	9	9
ARR5	9	9	9	9	9	9
ARR7	9	9	9	9	8	9
EF2	9	9	9	9	9	9
WUS 3'	5	4	7	8	3	5
WUS 5'	3	4	8	7	3	6

S1 Table
