# Supplementary material for: ARR22 overexpression can suppress plant Two-Component Regulatory Systems
Source: PLoS One. 2019 Feb 11;14(2):e0212056. doi: 10.1371/journal.pone.0212056 (PMC6370222; doi:10.1371/journal.pone.0212056)
Supplement: S1 Text — (PDF) [file pone.0212056.s032.pdf]

S1 Text: GARP sites in *ARR5p* and *WUSp*

GARP binding sites

core 5'-GAT-3' (reverse strand: 5'-ATC-3') and

degenerate extended: 5'-RGATY-3' which is (A/G)GAT(T/C) as described in (Hosoda et al., 2002; Imamura et al., 2003; Ramireddy et al., 2013; Sakai et al., 2001; Taniguchi et al., 2007; Veerabagu et al., 2012).

ARR1 extended motif (ECRM) 5'-AAGATYTT-3' which is AAGAT(T/C)TT (Sakai et al., 2001; Taniguchi et al., 2007) is very similar to the 5'-AAGATTT-3' one half extended CRM (hECRM) motif of (Ramireddy et al., 2013).

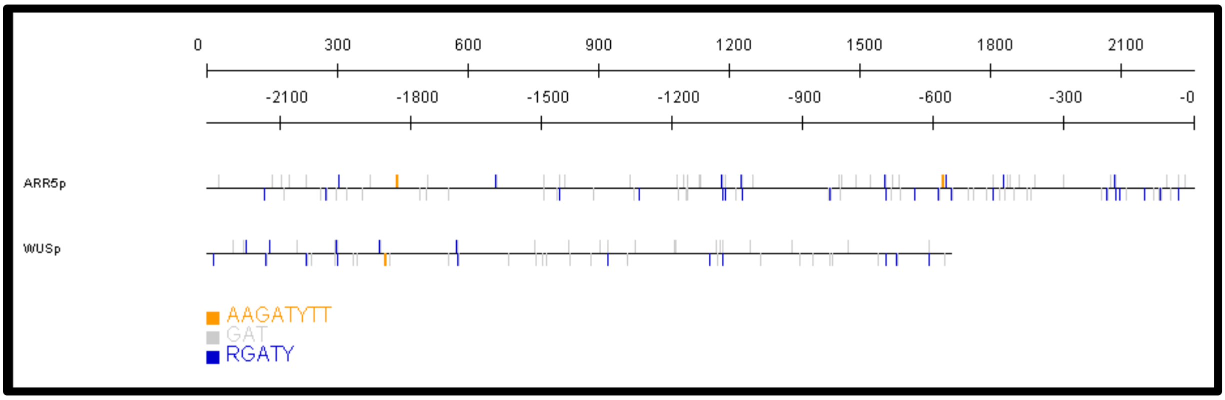

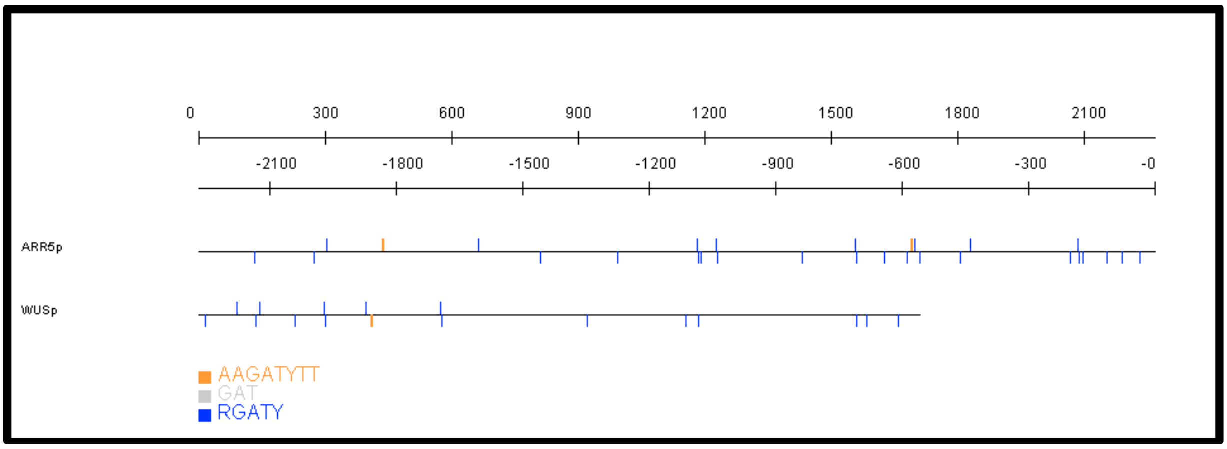

Maps made with : Stein Aerts, Peter Van Loo, Gert Thijs, Herbert Mayer, Rainer de Martin, Yves Moreau and Bart De Moor (2005) "TOUCAN 2: the all-inclusive open source workbench for regulatory sequence analysis". Nucl Acids Res, vol. 33 (Web Server issue), W393-6.

GARP sites in *ARR5p*:

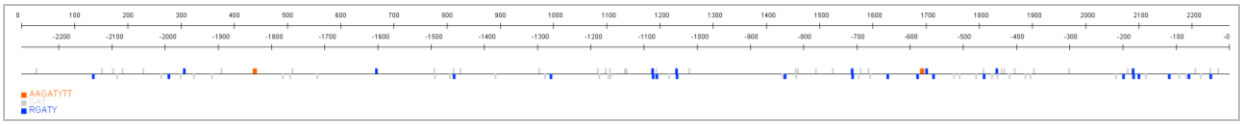

Motif counts and statistic Generated by MotifMapperVBA-WsH PackageVersion
Orientation as: F = Forward, R = Reverse, B = both Fwd and Rev combinations, U = undetermined, P = palindrome, D = dyad, D(all) = dyad with all forward combinations

Sequence Identifier	Sequence Length	Motif	Type	Total Counts
ARR5p	2270	GAT	F	44
ARR5p	2270	ATC	R	52
ARR5p	2270	GAT(ATC)	B	96
ARR5p	2270	RGATY	F	10
ARR5p	2270	RATCY	R	19
ARR5p	2270	RGATY(RATCY)	B	29
ARR5p	2270	AAGATYTT	F	2
ARR5p	2270	AAGATYTT(AARATCTT)	B	2

Output excerpt from Motif Mapper (5.2.4.01). http://www.uni-tuebingen.de/fakultaeten/mathematisch-naturwissenschaftliche-fakultaet/fachbereiche/zentren/zmbp/plant-physiology/research-groups/harter/berendzen/scripts.html

Cloning related/vector

Borders of Intergenic non-coding sequence

UTR

Amino acid encoding

>ARR5p

GGATCCgagttcgcggttcgacctaaactatatgatttttcaaaagaaaacaccatttagtctaaaatgtatttgacaaaaaaaaaagtctaaaatgtaaatt
cactaagcaacaaaaacaaaattgtaagctgtaataatcttgtacatttatagataattattaaataatttcagatatataatcaatgaatgatgaataacaattg
aatataagtaattcagttactcacgattatttatagaataggagaattcataaaataaatcgagtatttgtaatccataacttaaaatttctatatcaaaagattac
gtacgtagagaaatcaaaatttggaccccgaatataattttgaataatcatatattagcttattgattctcattttataacattttcctttgtcatatagtgaaattga
gacacacacaaagaaaagattttttttttaagtataatacaagaacatttttatttagaccatgctaacatcttcccaattttatcagatgtcccaaaagaaccta
atacaacaacatgcatgtaccaattcatatcacatagacagttaacacgaaagttcgttggtttttgtaaggaaaccaataaagcatatttgtcttcagcctctcat
actatttgagaggtaaaaaccgagaccattaggattattctttatagaatgttttggtgcctaacaaattttactggcatttctttttttgccataaactgttaagag
gtttatttatgaattgttaacttaatttctttgatcaaacaatagtaagttctataaatgtatcttgtgatctttgactatgatagacactaagctcggactcatgtac
atagccaaaacactcatatatattaaaacaataatttcatcggtaaactcatgttgtttccaaatatacaagtagagaaaacgtgtagacttactctgaagggac
acgcatatacatactgataaacaaccatcatggagaaatccgagccgtttctctgcaagccaaaccaaatttgacatatatagtaaggtcatgtgctctatacac
ctagtacctcacattcatttgatatctcctctagagatacatcgatcatagtcatattttcagctttcaatgatgatttagtcattaccattcttgaaatatattggtcg
agtttagtccatagatcttcgatcctgtcgtgacaaagccttccatcaattgtcaaaagatctctcacgtgtggaaatactcgatgcgtgttgctaaatatgtgaat
aaaagaacgccaaatttatagaaaaagcgaattaaaattcaagtaaacatatgaatatttttcaagaaaagtgaattgaaattttattttcatgaaaacttactat
tttttttttttttgaacactcatgaaaacttactattaactcatcaatcttatgccattcgaccacgatcgatagtagaggcaactattatggtacagttacgaga
tggacaaacaacaataacttgaaaaataatgatttttgttttgtttctagaaatatacacatacggatcttgaatttatctgattgcgcaactagagatatcaaca

ttgaaattcattcgcccaaatagtcaatctggcggtcacatgtcagctaacttttccaacaatgttcattaattaattaacaatctcaaagattttgtagattgaaat
acaaatcttctctctgtggtacatttcttgaaaaatgggaaaatcaagaaagtatcgaaaatgtacaaaaataaaaagaaatgaatcaaagtagccatgatctt
gacaacaataatcgagagagatcgtcatgatacgatttccctcatccaaaattgattttatttcccttcccaaatcaaacatatcatatgatttcaccactcaccatt
acttgactattctcaacaaaaaaaatattaaaaaactttatgactttgattttatttttatttgaagtttagccaaaatttgaaaatatgacttttgagaagaaaaca
gaataaacaaataattagccacgcgctatcagacagacaaaatcccacagatatgcaaagatctctcagaatcctctccccatatcatatttttctcttttccctct
ccttctttcttcctttataaatccatttattctcctctcatctctcagcaaaatcaaatcctcatagttgattctctctatctctctcacgagtcacgatcctactcttctt
gatATGGCTGAGGTTTTGCGTgCCatgG

GARP sites in *WUSp*:

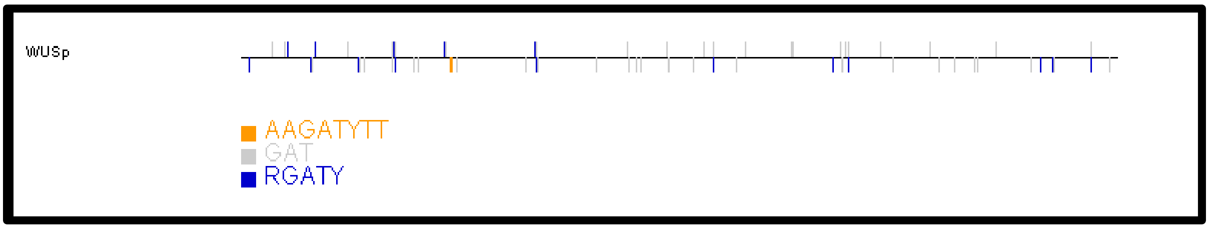

Motif counts and statistic Generated by MotifMapperVBA-WsH PackageVersion
Orientation as: F = Forward, R = Reverse, B = both Fwd and Rev combinations, U = undetermined, P = palindrome, D = dyad, D(all) = dyad with all forward combinations

Sequence Identifier	Sequence Length	Motif	Type	Total Counts
WUSp	1712	GAT	F	23
WUSp	1712	ATC	R	34
WUSp	1712	GAT(ATC)	B	57
WUSp	1712	RGATY	F	5
WUSp	1712	RATCY	R	12
WUSp	1712	RGATY(RATCY)	B	17
WUSp	1712	AARATCTT	R	1
WUSp	1712	AAGATYTT(AARATCTT)	B	1

Cloning related/vector

Borders of Intergenic non-coding sequence

UTR

Amino acid encoding

>WUSp

GGATCCATCGATGAATTCTTATCCAATATAATCGACTAAagttaaaaaatgagtaatccaccagtctaactaaatactttctttaccaacaaaa
atttgggatacaactgagtttatttaggtgataaaagattataagacttaaccttttaacaaaaaaaattttttaagacttcaatccttagattgttttccaaatatt
aatagttacaatagttagtttgtaaaacctatttttaattttccgatactctttgcacgttgtgttgaatccgagaaatcatgggggctatataaacaaaactatact
atgaaatagccaagcaacttcagtgatcggatctttggtttttttattttaaaaaaaataaatattttatcagccgtatcttcagggccattccccataaaaactata
atagcttctatgaaatttgtagattcctaaaaaaatctttatatatcttttatatacaatatgtggctgttacgtagaacaataagactatacaaaagttgggtattg
ttccttttcaattacaaaagtcatattcactaaaacaaagcccttcaaactttttaaaaaaaatgaataaatcacgcatttatacaaaaagatccatccatgctcct

ataaattcgagcatataattaactaaagaatactcaacatgttcataagtacacctgtcttcacactcgtttcacacattgtaattgacataaattttaatatctttt
aaagcaaagcataagtaaaattatatagagtatttcttcaacacatttccggacgatatcaccacgaaaaatatcctatatcacctatgtgtcatgactcatgacc
ataaggaaggaaaaatgtatttttatgatcaaacggttttcactttttttattattcattctagaaaaaaattataatcgttttctttcattcctacgatacaataattc
ataacgatcttttagaatttttgttttgttttctgtgtgtatgggtaaatatctttcatacgtagacagatacataacaattaaagctggtgaagtaataattgtgaag
agacagagaaattgaaaaaagagagtttgtttttcgtctaagtaaataccgatgatgccctcacctttctaattccctcctttgtccctttccattttcatgtttatttc
taacttctctgtccctttcaatccttctccaccagatatcctctgattcgatcccccacaaatttttttcataaaaattattattattctcactaaaactctcaagagtag
tttcgatttttcctctgaataaaaaaaaaatcaaataatatatttgacaagagaatttccttcctaaacataacattttatttgaatgttaattcaattgattatttttct
ttcttatatcctttttctttggcaaaatttatttcttatatccaagcacatttttcaatagggttttgtatgtatgaaatcatatctacatgcataaaagaataatatagt
gaataactgattttctagaatattgtaaaaccttctttttgggtccccatttatatatataactcaataccttactatctctctatatatatataatcttctctctcacac
aaaacctaaaatctctttactaccagcaagttgttttcttgctaacttcaaacttctctttctcttgttcctctctaagtcttgatcttatttaccgttaactttgtgaaca
aaagtcgaatcaaacacacATGaCCatgG

REFS

Hosoda, K., et al. (2002). Molecular structure of the GARP family of plant Myb-related DNA binding motifs of the Arabidopsis response regulators. The Plant cell 14:2015-2029.

Imamura, A., Kiba, T., Tajima, Y., Yamashino, T., and Mizuno, T. (2003). In vivo and in vitro characterization of the ARR11 response regulator implicated in the His-to-Asp phosphorelay signal transduction in Arabidopsis thaliana. Plant & cell physiology 44:122-131.

Ramireddy, E., Brenner, W.G., Pfeifer, A., Heyl, A., and Schmulling, T. (2013). In planta analysis of a cis-regulatory cytokinin response motif in Arabidopsis and identification of a novel enhancer sequence. Plant & cell physiology 54:1079-1092.

Sakai, H., et al. (2001). ARR1, a transcription factor for genes immediately responsive to cytokinins. Science 294:1519-1521.

Taniguchi, M., Sasaki, N., Tsuge, T., Aoyama, T., and Oka, A. (2007). ARR1 directly activates cytokinin response genes that encode proteins with diverse regulatory functions. Plant & cell physiology 48:263-277.

Veerabagu, M., et al. (2012). The Arabidopsis B-type response regulator 18 homomerizes and positively regulates cytokinin responses. The Plant journal : for cell and molecular biology 72:721-731.
